# Association of BCG, DTP, and measles containing vaccines with childhood mortality: systematic review

**DOI:** 10.1136/bmj.i5170

**Published:** 2016-10-13

**Authors:** Julian P T Higgins, Karla Soares-Weiser, José A López-López, Artemisia Kakourou, Katherine Chaplin, Hannah Christensen, Natasha K Martin, Jonathan A C Sterne, Arthur L Reingold

**Affiliations:** 1 School of Social and Community Medicine, University of Bristol, Bristol BS8 2PS, UK; 2 Cochrane, St Albans House, London SW1Y 4QX, UK; 3 Department of Hygiene and Epidemiology, University of Ioannina School of Medicine, Ioannina, Greece; 4 Department of Global Health and Development, London School of Hygiene and Tropical Medicine, London WC1E 7HT, UK; 5 Division of Epidemiology, School of Public Health, University of California, Berkeley, CA 94720-7358, USA

## Abstract

**Objectives** To evaluate the effects on non-specific and all cause mortality, in children under 5, of Bacillus Calmette-Guérin (BCG), diphtheria-tetanus-pertussis (DTP), and standard titre measles containing vaccines (MCV); to examine internal validity of the studies; and to examine any modifying effects of sex, age, vaccine sequence, and co-administration of vitamin A.

**Design** Systematic review, including assessment of risk of bias, and meta-analyses of similar studies.

**Study eligibility criteria** Clinical trials, cohort studies, and case-control studies of the effects on mortality of BCG, whole cell DTP, and standard titre MCV in children under 5.

**Data sources** Searches of Medline, Embase, Global Index Medicus, and the WHO International Clinical Trials Registry Platform, supplemented by contact with experts in the field. To avoid overlap in children studied across the included articles, findings from non-overlapping birth cohorts were identified.

**Results** Results from 34 birth cohorts were identified. Most evidence was from observational studies, with some from short term clinical trials. Most studies reported on all cause (rather than non-specific) mortality. Receipt of BCG vaccine was associated with a reduction in all cause mortality: the average relative risks were 0.70 (95% confidence interval 0.49 to 1.01) from five clinical trials and 0.47 (0.32 to 0.69) from nine observational studies at high risk of bias. Receipt of DTP (almost always with oral polio vaccine) was associated with a possible increase in all cause mortality on average (relative risk 1.38, 0.92 to 2.08) from 10 studies at high risk of bias; this effect seemed stronger in girls than in boys. Receipt of standard titre MCV was associated with a reduction in all cause mortality (relative risks 0.74 (0.51 to 1.07) from four clinical trials and 0.51 (0.42 to 0.63) from 18 observational studies at high risk of bias); this effect seemed stronger in girls than in boys. Seven observational studies, assessed as being at high risk of bias, have compared sequences of vaccines; results of a subset of these suggest that administering DTP with or after MCV may be associated with higher mortality than administering it before MCV.

**Conclusions** Evidence suggests that receipt of BCG and MCV reduce overall mortality by more than would be expected through their effects on the diseases they prevent, and receipt of DTP may be associated with an increase in all cause mortality. Although efforts should be made to ensure that all children are immunised on schedule with BCG, DTP, and MCV, randomised trials are needed to compare the effects of different sequences.

## Introduction

An increasing number of vaccines targeting some of the leading causes of morbidity and mortality are reaching the world’s children. Although vaccines such as those against measles, diphtheria-tetanus-pertussis (DTP), and polio are widely understood to have reduced the burden of the diseases they target, studies have suggested that some of the vaccines routinely administered to infants and children also affect the risk of illness and death from conditions other than the specific infectious diseases they are designed to prevent.^1^ ^2^ Among hypotheses concerning these “non-specific effects” of vaccines are that, under some circumstances, some vaccines (for example, measles and Bacillus Calmette-Guérin (BCG)) lower subsequent risk, whereas others (such as DTP) increase subsequent risk of illness and death from other causes. It is further postulated that the magnitude of these effects depends on other factors, including sex and vitamin A supplementation status. The potential for non-specific vaccine effects has led some authors to question whether the vaccination schedules currently recommended by the World Health Organization should be modified.^3^ ^4^

WHO recommends that BCG should be administered as soon as possible after birth, that whole cell DTP should be administered after six weeks, with two further doses at intervals of four to eight weeks, and that measles containing vaccines (MCV) be administered at nine to 12 months, with a further dose given at least four weeks later.^3^ We aimed to quantify the effects of these three vaccines on mortality from causes other than those the vaccine is designed to prevent and on all cause mortality. Randomised trials testing these effects have been difficult or impossible to conduct. As a result, many of the studies testing these hypotheses have been observational in nature. We therefore included both randomised trials and observational studies and aimed also to evaluate the potential for bias in the available evidence.

## Methods

### Study eligibility and selection

We followed a protocol that was published online in advance^5^; further details of study methods have subsequently been published.^6^ We sought clinical trials (randomised or quasi-randomised), cohort studies, and case-control studies comparing children who were and were not given one of the three vaccines. Studies in which there was simultaneous administration of another vaccine were eligible. Studies had to report mortality data for children up to 5 years of age. We excluded children who had received medium or high titre MCV, as these are not currently used. We restricted eligibility to primary research articles (published or unpublished), reanalyses of primary studies reported in methodological articles, and follow-up commentaries and letters written by the authors of the original studies. We excluded results available only in reviews and meta-analyses, as well as commentaries or letters not written by study authors.

We searched Medline and Embase (to November 2012 with no restriction on start date), Global Index Medicus (to March 2013), and the WHO International Clinical Trials Registry Platform (to March 2013). The search strategy is available in an online supplement (appendix 1). Searches were supplemented by contact with experts in the field. Search results were uploaded to a web based system (DistillerSR, www.systematic-review.ca). Titles and abstracts were inspected independently by two reviewers, and the full text of potentially relevant articles was obtained. Articles underwent two phases of inspection, in each case by two reviewers working independently. Discrepancies were resolved by a principal investigator.

### Data collection and management

Two reviewers collected data independently, using a data extraction form within the web based system. Further data collection was done by a statistician, focusing on extraction of mortality outcome data. In addition to studies’ characteristics, we collected adjusted and unadjusted relative risk estimates and all available effect measures stratified by sex (or computed them where the required information was reported) and by receipt or not of vitamin A supplementation.

Considerable overlap existed in children studied across the included articles, so multiple results were available for some groups of children. To avoid double counting, we grouped children into birth cohorts by geographical location and time period, and we grouped all articles relating to the same birth cohort. We devised an algorithm to select one primary result for each vaccine from each birth cohort (appendix 2). This favoured results relating to vaccination received according to the sequence implied by WHO recommendations (BCG at birth, then DTP, then MCV), comparisons of administration versus no administration of the vaccine, randomised comparisons, general population cohorts, adjusted estimates, and larger sample sizes. We applied the same principles to extract data for examining interactions and making comparisons of sequences; there was substantially less multiplicity for these results, although effect estimates had to be computed from available results to make the desired comparisons of vaccine sequences.

### Risk of bias assessment

We used the Cochrane tool for assessing risk of bias in clinical trials.^7^ For observational studies, we used a new tool that is motivated by considerations of causal inference in epidemiology,^8^ additionally informed by methodological considerations specific to this research area.^9^ ^10^ We pre-specified potential confounders as age and sex of the child, child’s health (including nutritional status and birth weight), and socioeconomic status (including poverty, education, and hygiene conditions) and potentially important co-interventions as malaria interventions, de-worming, micronutrient supplements, breast feeding, hygiene programmes, and other vaccinations. We assessed risks of bias in seven domains, facilitated by consideration of pertinent “signalling” questions, including definition of vaccination status, likelihood of subsequent vaccinations, and use of landmark or retrospective approaches to analysis.^9^ Within each domain, we rated risk of bias as “low” (comparable to a well performed randomised trial), “moderate” (sound for an observational study), “high” (there are important problems), or “very high” (the study is too problematic to provide useful evidence). We excluded results of studies at very high a risk of bias from syntheses, and they do not contribute to our conclusions. We used the same categories for risk of bias in clinical trials.

### Statistical methods

We estimated a relative risk for each independent birth cohort (measured using hazard ratio, rate ratio, risk ratio, or odds ratio, in order of preference), computed from summary statistics and subgroups where necessary. When combining information across subgroups within a birth cohort, we used fixed effect meta-analysis. When combining information across overlapping analyses, we averaged the effect size and its variance (on the log scale). We used methods described by Greenland and Longnecker when we used a different reference group from that originally reported.^11^ Results in forest plots are relative risk estimates and 95% confidence intervals. Meta-analyses used standard fixed effect and random effects inverse variance weighted averages with a moment estimate of between studies variance,^12^ separately for clinical trials and observational studies, with the extent of inconsistency measured using I^2^ statistics and between study heterogeneity represented in prediction intervals.^13^ We present mean effects from random effects analyses in the text. Sensitivity analyses using alternative meta-analysis approaches are presented in online supplementary material. Each meta-analysis included too few studies for funnel plots and associated tests to be informative.^14^

To examine differences in vaccine effect by sex, we computed within study interactions as the difference between the vaccine effects for boys and girls or as the difference between sex effects for vaccinated and unvaccinated children. We took a similar approach to examine differential vaccine effects by vitamin A supplementation status. In further analyses, we considered various sequences of vaccines, to examine outcomes when the usual sequence of vaccines had been compared with alternatives (simultaneous administration of BCG and DTP, BCG after DTP, BCG with or after DTP, simultaneous administration of DTP and MCV, DTP after MCV, DTP with or after MCV).

### Patient involvement

No patients were involved in setting the research question or the outcome measures, nor were they involved in developing plans for design or implementation of the study. No patients were asked to advise on interpretation or writing up of results. There are no plans to disseminate the results of the research to study participants or the relevant patient community.

## Results

### Included studies

Detailed results of the review are available elsewhere.^6^ We included 68 articles reporting results for the effects of the three vaccines on overall mortality, originating from 34 birth cohorts (fig 1). Twenty one cohorts were from Africa (including eight cohorts (described in 37 articles) from Guinea Bissau and four (in four articles) from Senegal), three were from North America, eight from south or southeast Asia, one from Papua New Guinea, and one from Haiti. For effects on overall mortality, we identified 18 results (17 birth cohorts) for BCG vaccine, 17 results (17 birth cohorts) for DTP vaccine, and 29 results (27 birth cohorts) for MCV. Five results for BCG vaccine were from clinical trials, as were four results for MCV. Three, zero, and seven articles reported results for non-specific mortality for BCG, DTP, and MCV, respectively. Characteristics of the birth cohorts contributing data for each vaccine are available in an online supplement (appendix 3), along with a brief summary of excluded studies (appendix 4).

**Figure f1:**
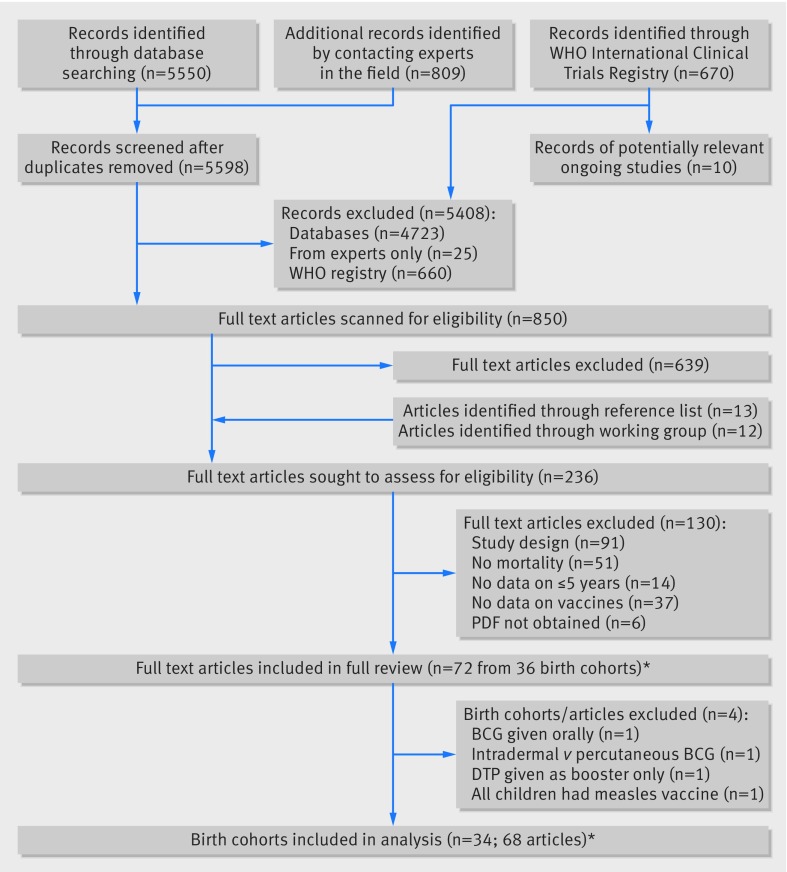
**Fig 1** Flow diagram summarising study selection process. *34 further full text articles contained additional relevant information

### Risk of bias in included studies

Methodological features and assessments of risk of bias are presented in an online supplement (appendix 5). Of the nine clinical trial results, we judged two for BCG to be at low risk of bias and the other seven (three for BCG and four for MCV) to be at moderate risk of bias. All of the results from observational studies were judged to be at high risk of bias (that is, there are important problems) or at very high risk of bias (that is, too problematic to provide useful evidence). The main potential sources of risk of bias were confounding (no studies were considered to have overcome this, particularly as sicker children are less likely to be vaccinated); misclassification bias relating to determination of non-vaccination status; bias arising from selection of participants after vaccines were received (therefore, after they could have affected mortality); co-interventions, including administration of other vaccines covered by the review; and misclassification bias relating to lack of information about vaccinations administered (including “survival bias” arising from a retrospective approach to the analysis being taken). We regard the estimates of interaction (for example, for differences by sex) to be much less affected by bias, because we expect that the biases affecting direct estimates of vaccine effects are likely to be similar across subgroups within a study (for example, similar in boys and girls).

### Effect of BCG vaccine on overall mortality

Five clinical trials, 12 cohort studies, and one case-control study compared mortality rates among BCG vaccinated and BCG non-vaccinated children (fig 2). We considered four results from cohort studies to be at very high risk of bias and excluded them from meta-analyses. The clinical trial results, including two at low risk of bias in low birthweight infants and two in Native American children in the 1930s and 40s, suggested a beneficial effect of BCG on mortality (average relative risk 0.70, 95% confidence interval 0.49 to 1.01). The clinical trials in low birthweight infants, which were the two studies judged to be at low risk of bias, gave a combined relative risk of 0.52 (0.33 to 0.82). The average relative risk for the nine observational studies (follow-up mostly within the first year of life) was 0.47 (0.32 to 0.69; inconsistency (I^2^)=63%), although all these studies were considered to be at high risk of bias. Results did not change materially when we used different statistical methods (appendix 6).

**Figure f2:**
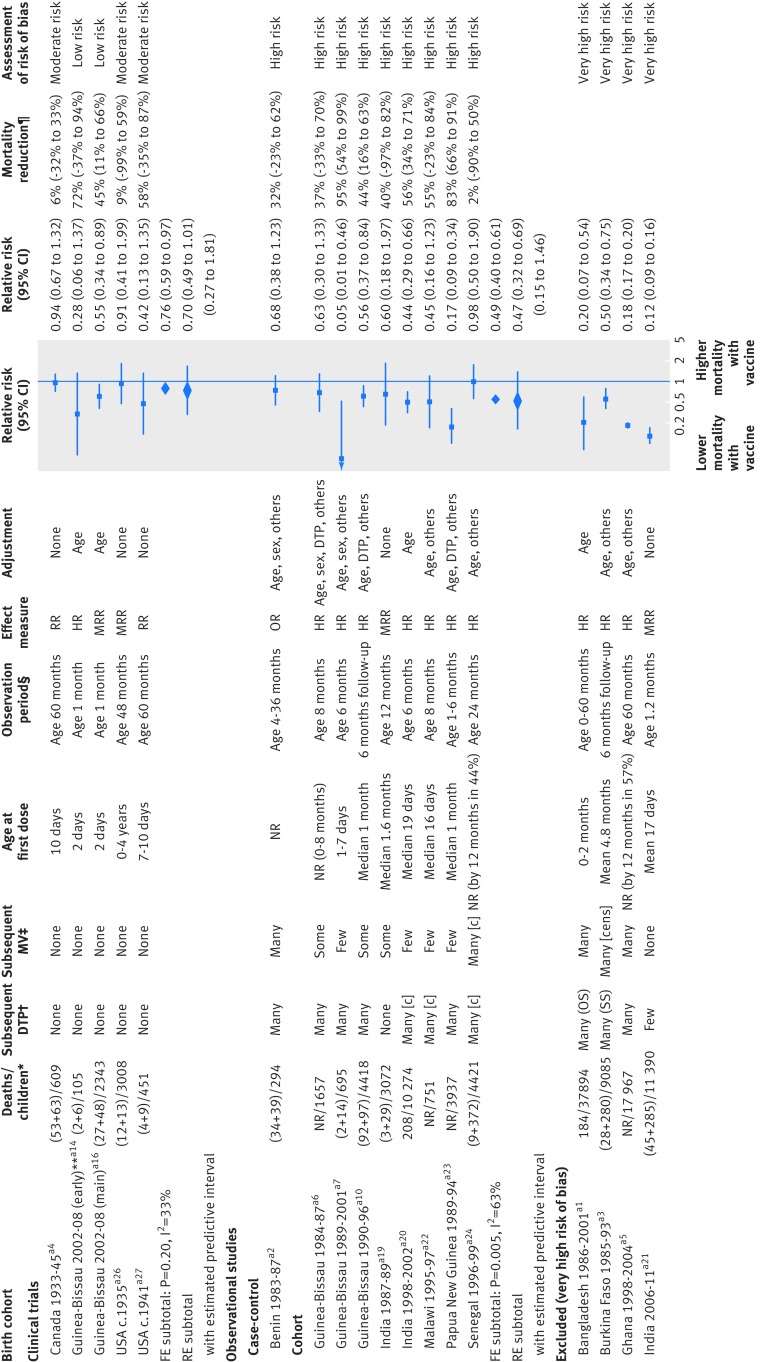
**Fig 2** Forest plot for BCG and all cause mortality. c=event was censored in analysis; FE=fixed effect meta-analysis method; HR=hazard ratio; MRR=mortality rate ratio; OR=odds ratio; OS=often received simultaneously with DTP; RE=random effects meta-analysis method; RR=relative risk; SS=sometimes received simultaneously with DTP. *(BCG deaths+non-BCG deaths)/total children or total deaths/total children. †Proportion of children likely to receive DTP during period of observation. ‡Proportion of children likely to receive MCV during period of observation. §Period of observation applicable to result presented in forest plot, aiming to capture effect of BCG with minimal impact of subsequent vaccinations; full study may have had longer follow-up. ¶Computed as (1−RR)×100%; non-negative number describes proportion of deaths prevented by vaccine; negative number reflects higher death rate among vaccinated children (for example, if vaccine efficacy is −100%, then an additional 100% of deaths that would have occurred without vaccine would occur with vaccine). **Early phase of trial stopped prematurely because of faulty randomisation procedure in one centre. ††(Subsequent) main trial phase with larger sample size (both phases in low birthweight infants only). In two cohort studies with “none” as adjustment for confounding, unadjusted rate ratios were computed from rates presented in article. Reference numbers correspond to those in appendix 3

### Effect of DTP vaccine on overall mortality

Sixteen cohort studies and one case-control study compared receipt of DTP with no DTP (fig 3). Oral polio vaccine was known to be administered concomitantly with DTP in most studies; three studies did not report co-administration of oral polio vaccone.^15^ ^16^ ^17^ No clinical trials were identified. We considered seven results from cohort studies to be at very high risk of bias and excluded them from meta-analyses. The remaining 10 studies produced diverse results (I^2^=71%), ranging from halving of to fourfold increase in mortality risk. Most studies indicated that receipt of DTP was associated with higher mortality, and three individual results had 95% confidence intervals that excluded no effect (one lower mortality, two higher mortality). The average relative risk was 1.38 (0.92 to 2.08) among these 10 studies, all assessed as being at high risk of bias. Results did not change materially when we used different statistical methods (appendix 6). The mortality rate was very high among unvaccinated children in the Papua New Guinea study,^18^ and two referees had notable concerns about this study. Excluding it from the meta-analysis gave a relative risk of 1.36 (1.09 to 1.68).

**Figure f3:**
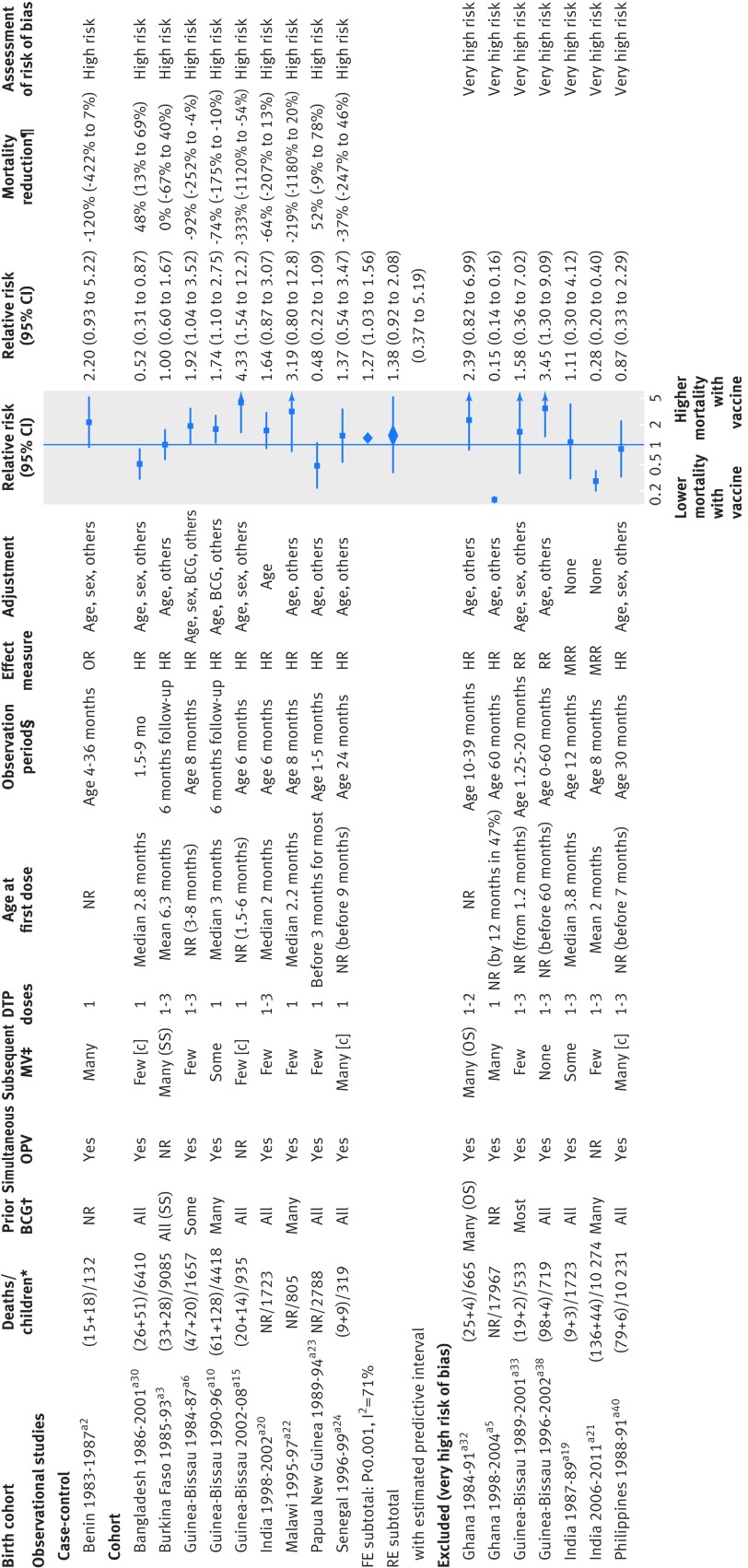
**Fig 3** Forest plot for DTP and all cause mortality. c=event was censored in analysis; FE=fixed effect meta-analysis method; HR=hazard ratio; MRR=mortality rate ratio; OR=odds ratio; OS=often received simultaneously with DTP; RE=random effects meta-analysis method; RR=relative risk; SS=sometimes received simultaneously with DTP. *(DTP deaths+non-DTP deaths)/total children or total deaths/total children. †Whether children studied had received BCG. ‡Proportion of children likely to receive MCV during period of observation; §Period of observation applicable to result presented in forest plot, aiming to capture effect of DTP with minimal impact of subsequent vaccinations; full study may have had longer follow-up. ¶Computed as (1−RR)×100%; non-negative number describes proportion of deaths prevented by vaccine; negative number reflects higher death rate among vaccinated children (for example, if vaccine efficacy is −100%, then an additional 100% of deaths that would have occurred without vaccine would occur with vaccine). In two cohort studies with “none” as adjustment for confounding, unadjusted rate ratios were computed from rates presented in article. Reference numbers correspond to those in appendix 3

### Effect of MCV on overall mortality

Four (randomised) clinical trials, 23 cohort studies, and two case-control studies compared children who had or had not received MCV (fig 4). We considered seven results from cohort studies to be at very high risk of bias and excluded them from meta-analyses. In three clinical trials in Guinea-Bissau, we limited follow-up to nine months, at which point children in the control group received MCV. Owing to the short follow-up, numbers of deaths were low and the findings inconclusive. Directions of effect in these trials, as well as in a fourth clinical trial in Nigeria, pointed towards a beneficial effect of receipt of MCV (relative risk 0.74, 0.51 to 1.07; I^2^=0%). The 18 observational studies that were not excluded consistently provided estimates indicating that MCV was associated with lower mortality within the first two to five years of life, with average halving of mortality risk (relative risk 0.51, 0.42 to 0.63; I^2^=64%). We considered all of these studies to be at high risk of bias. Results did not change materially when we used different statistical methods (appendix 6). Results after deaths from measles were removed or censored (not shown here; see full report for details^6^) suggested that these effects, if real, were not fully explained by deaths due to measles.

**Figure f4:**
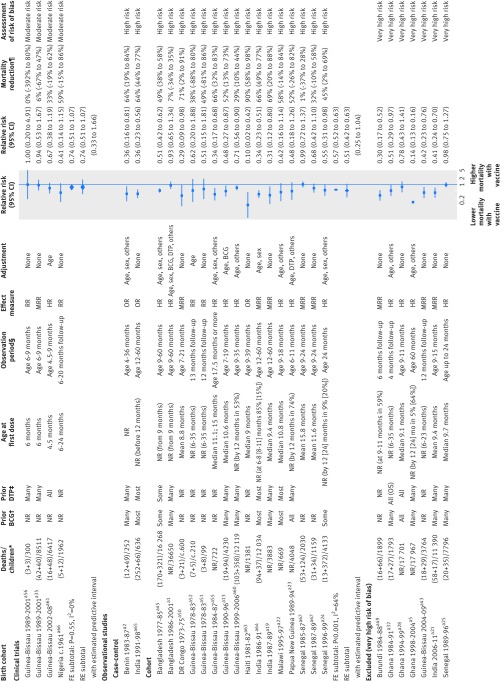
**Fig 4** Forest plot for measles containing vaccine (MCV) and all cause mortality. c=censored in analysis; FE=fixed effect meta-analysis method; HR=hazard ratio; MRR=mortality rate ratio; OR=odds ratio; OS=often received simultaneously with DTP; RE=random effects meta-analysis method; RR=relative risk. *(MCV deaths+non-MCV deaths)/total children or total deaths/total children. †Whether children studied had received BCG. ‡Whether children studied had received DTP. §Period of observation applicable to result presented in forest plot, aiming to capture effect of MCV with minimal impact of subsequent vaccinations; study may have had longer follow-up. ¶Computed as (1−effect size)×100%; non-negative number describes proportion of deaths prevented by vaccine; negative number reflects higher death rate among vaccinated children (for example, if vaccine efficacy is −100%, then an additional 100% of deaths that would have occurred without vaccine would occur with vaccine). (a) and (b) for Guinea-Bissau 1989-99 and Guinea-Bissau 1978-83 each reflect two results from same birth cohort in non-overlapping groups of children. In most observational studies with “none” as adjustment for confounding, unadjusted rate ratios were computed from rates presented in article. Reference numbers correspond to those in appendix 3

### Effects of different vaccine sequences on overall mortality

We compared the standard vaccination sequence (BCG followed by DTP followed by MCV) with variants in which BCG was received either with or after DTP or DTP was received either with or after MCV (fig 5). Three cohort studies compared DTP received simultaneously with BCG against DTP after BCG (P Aaby, unpublished manuscript).^19^ ^20^ They suggested that simultaneous administration may be associated with lower mortality (relative risk 0.52, 0.34 to 0.80; I^2^=0%). Three studies compared BCG received after DTP against DTP after BCG (P Aaby, unpublished manuscript).^19^ ^20^ No clear differences were apparent. These three studies, plus one other reporting on two different age groups,^18^ compared receipt of BCG vaccine with or after DTP against DTP after BCG. The summary effect was a relative risk of 0.60 (0.42 to 0.86). We considered all these 11 results to be at high risk of bias, and five of them were not adjusted for age.

**Figure f5:**
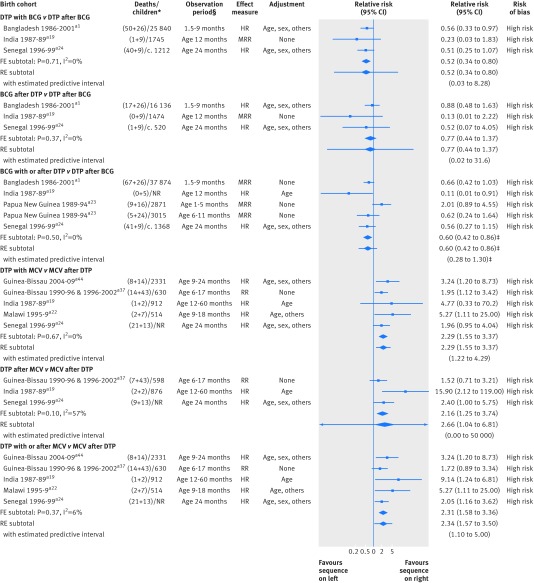
**Fig 5** Forest plot for comparisons of different sequences of vaccines and all cause mortality. FE=fixed effect meta-analysis method; HR=hazard ratio; OR=odds ratio; RE=random effects meta-analysis method; RR=relative risk. *(MCV deaths+non-MCV deaths)/total children or total deaths/total children. †Period of observation applicable to result presented in forest plot, aiming to capture effect with minimal impact of subsequent vaccinations; full study may have had longer period of follow-up. ‡Meta-analysis excludes one Papua New Guinea result (1-5 months) to avoid double counting. In most observational studies with “none” as adjustment for confounding, unadjusted rate ratios were computed from rates presented in article. Results from Senegal 1996-99 were computed from full sample, rather than sample aged 9-24 months also reported. Reference numbers correspond to those in appendix 3

Five cohort studies compared DTP received simultaneously with MCV against MCV received after DTP.^19^ ^20^ ^21^ ^22^ ^23^ Their results suggested that simultaneous administration may be associated with higher mortality (relative risk 2.29, 1.55 to 3.37; I^2^=0%). Results of three studies that compared DTP after MCV against the standard sequence^19^ ^20^ ^22^ suggested that receiving DTP after MCV may be associated with higher mortality (relative risk 2.66, 1.04 to 6.81; I^2^=57%). Five studies provided results for a comparison of DTP with or after MCV against MCV after DTP (relative risk 2.34, 1.57 to 3.50**]**; I^2^=6%). Again, we judged these 13 results to be at high risk of bias, and three of them were not adjusted for age of the children.

### Effect modification

#### BCG vaccine

Nine studies (one clinical trial and eight cohort studies) compared BCG with no BCG separately for boys and girls. We found no apparent difference in effect between boys and girls (ratio of relative risks 1.02, 0.73 to 1.41; I^2^=0%; fig 6(a)). The average age at which BCG vaccination was administered varied across studies, from soon after birth to 4.8 months. Two cohort studies reported effects for children vaccinated at different ages: the beneficial effect of BCG seemed to decrease as age at vaccination increased (fig 7(a)). The two clinical trials comparing BCG at birth with delayed BCG (recommended at six weeks) among low birthweight infants suggested a possible benefit of early over delayed BCG.^24^ ^25^ We found insufficient evidence to determine whether the effect of BCG varies with vitamin A supplementation status.

**Figure f6:**
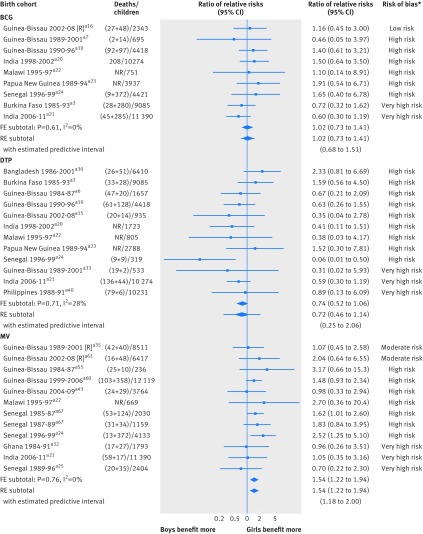
**Fig 6** Differential vaccine effects by sex: meta-analyses of within study estimates of interaction. FE=fixed effect meta-analysis method; R=randomised trial; RE=random effects meta-analysis method; RR=relative risk. *Risk of bias assessments for main effects of the vaccine (from fig 2 to fig 4). Reference numbers correspond to those in appendix 3

**Figure f7:**
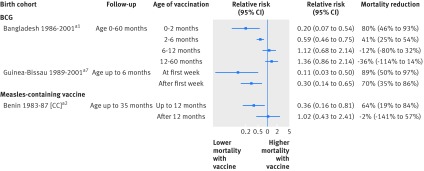
**Fig 7** Differential vaccine effects by age at vaccination. CC=case-control study; RR=relative risk. Reference numbers correspond to those in appendix 3

#### DTP vaccine

Twelve of the 16 cohort studies compared DTP with no DTP separately for boys and girls (fig 6(b)). Only one study found evidence of a difference,^20^ with 95% confidence intervals indicating that boys benefit more (or are harmed less) than girls. None of the other studies found similarly strong evidence of a difference in either direction; eight of these found a tendency for receipt of DTP to be associated with a more harmful effect in girls than boys. The overall ratio of relative risks was 0.72 (0.46 to 1.14; I^2^=28%). No studies reported results for different ages at DTP vaccination. We found insufficient evidence to determine whether any difference exists in effect of DTP according to vitamin A supplementation status.

#### Measles containing vaccine

Two clinical trials and 10 cohort studies compared MCV with no MCV separately for boys and girls (fig 6(c)). Effects in girls seemed to be more beneficial than those in boys (ratio of relative risks 1.54, 1.22 to 1.94; I^2^=0%). Where ages at vaccination were available, they were typically around 9 months, ranging from 4.5 months in a clinical trial to median 15.8 months in a cohort study. One case-control study reported larger effects in children vaccinated before rather than after 12 months (fig 7(b)). On the basis of three studies, there was no consistent difference in the effect of MCV according to (previous or concurrent) administration of vitamin A.

## Discussion

BCG, DTP, and MCV have prevented countless illnesses and deaths among infants and children worldwide, especially those living in the world’s poorest countries. We systematically reviewed evidence on associations between receipt of these vaccines and childhood mortality, with a focus on effects beyond those attributable to the targeted diseases. With few exceptions, the studies identified were observational in nature and thus prone to many well recognised forms of bias. Receipt of BCG and standard titre MCV was associated with a lower risk of all cause mortality, but receipt of DTP was associated with a higher risk of mortality in seven studies and a lower risk in two studies. In comparisons within studies, receipt of DTP was associated with a higher risk of mortality than receipt of BCG or MCV. The clinical trials of BCG included two in low birthweight infants, and together these indicated a reduction in mortality. The beneficial effect of receipt of MCV seemed to be greater among girls than boys. Evidence on modification of the effects of any of the three vaccines on the risk of all cause mortality by vitamin A supplementation status or age at vaccination was generally insufficient to allow conclusions to be drawn.

### Strengths and limitations of study

Our review provides a comprehensive evaluation of the evidence to date. We did an extensive search for studies and carefully addressed the overlap of children across multiple analysis reports. The assessment of potential bias is a difficult and subjective judgment, but we attempted to do this systematically with a detailed assessment tool; we quantified the evidence within strata defined by study design and potential for bias.

Although limited clinical trial evidence was available for BCG and MCV, it was broadly consistent with the larger body of evidence from observational studies. We excluded the randomised trials of high titre measles vaccine because it is not currently in use. The main limitation of our review relates to the risk of bias in the results of the included studies. Our review was also based only on our evaluation of written reports of the studies, and we did not contact authors for missing information.

Many types of bias may have influenced the results of the observational studies included here. Uncontrolled or poorly controlled confounding was a potential problem in all of them, including confounding at baseline (for example, because frail children may be less likely to be vaccinated), post-vaccination confounding (for example, due to co-interventions), and adjustment for different selections of potential confounders. Baseline confounding, if ignored, would tend to lead to bias towards a beneficial effect of the vaccine, because children with a worse prognosis generally tend to be vaccinated later or not vaccinated at all (sometimes described as “frailty bias”). We therefore prioritised effect estimates adjusted for baseline confounders.

Selection biases and information bias arising from misclassification of vaccination status were also causes of concern. Selection biases might be expected to operate in the opposite direction to baseline confounding. For instance, if children are recruited some time after vaccination, then early deaths among unvaccinated children—deaths that might have been prevented had the children been vaccinated—are not observed. Furthermore, censoring follow-up of children on receipt of a subsequent vaccination, as was done in some studies of DTP vaccination, may selectively remove observation time from children who have received the vaccine of interest and are well enough to receive the next one, introducing bias towards an adverse effect of the vaccine. Misclassification of vaccinated children as unvaccinated would typically lead to bias towards the null (no effect), as occurs when a “landmark” approach is taken to the analysis.^10^ However, systematic misclassification of dead children as unvaccinated would lead to a bias in favour of the vaccine, and this would not provide an explanation for the observed potentially harmful effect of DTP. Potential biases due to previous receipt, co-administration, and subsequent administration of other vaccines (for example, DTP or MCV when examining BCG) also exist. The direction of these biases depends on whether the other vaccines have beneficial or harmful effects, and we are not able to make assumptions about these effects in the context of this review. In summary, predicting the direction of bias for individual studies or the accumulated body of evidence is very difficult, as is estimation of its magnitude.

A further potential source of bias, which is particularly difficult to assess, is the selective reporting (and non-reporting) of results, both through mechanisms that lead articles to be written and published and through decisions about which results to present. This is known to be a major problem in randomised trials,^26^ and it is, in general, likely to be even more serious in observational studies. The similarity of meta-analysis estimates from fixed effect and random effects approaches provides some reassurance against an important relation between study size and magnitude of effect, but we do not consider this to be evidence against the presence of reporting biases.

Although most of the studies of DTP vaccine suggested that receipt of this vaccine was associated with an increased risk of all cause mortality in the period shortly after vaccination, it is not clear that this can be attributed to DTP vaccine because the available studies were observational and judged to be at high risk of bias. Furthermore, unlike for BCG and MCV, no randomised trials were available for DTP. We are also unable to separate the effects of DTP from those of oral polio vaccine because they were almost always co-administered.

Six of the studies examined all three of the vaccines, and their findings are shown in figure 8. We would expect many of the same types of bias to be present across the three comparisons within each study. In four of the studies, there is an apparent beneficial effect of BCG and MCV and an apparent harmful effect of DTP on mortality. We are unable to explain these patterns using information relating to potential risks of bias available in the study reports, and regard the findings to be a cause for concern.

**Figure f8:**
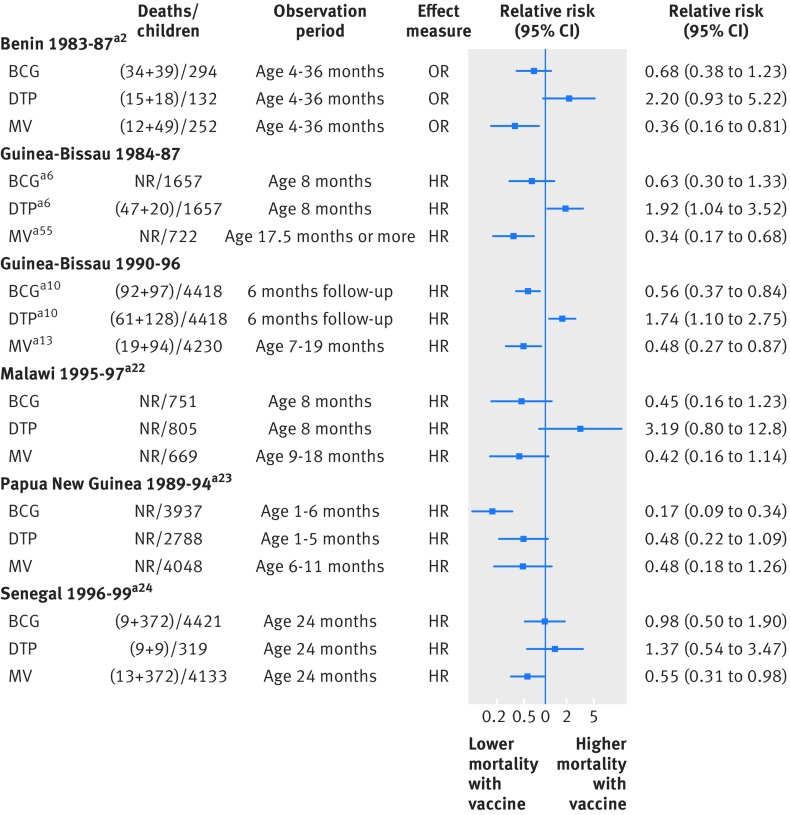
**Fig 8** Summary of results from studies examining all three vaccines. CC=case-control study; HR=hazard ratio; OR=odds ratio; RR=relative risk. Reference numbers correspond to those in appendix 3

### Interpretation and implications of findings

The findings should be interpreted in the context of the absolute risks of mortality reported by the included studies. Among the cohort studies of BCG vaccine, the mortality risk ranged from 1% over 12 months to 9% over 24 months. Assuming a 2% mortality risk over six months, vaccine relative risks of 0.5 and 0.75 would imply that there were 10 and five fewer deaths, respectively, per 1000 children during this period of time. Similarly, among the cohort studies of DTP, the mortality risk ranged from 0.7% over six months to 6% over 24 months. Assuming a 2% mortality risk over 12 months, vaccine relative risks of 1.2 and 1.4 would imply that there were four and eight extra deaths, respectively, per 1000 children during the subsequent year,

Findings from the studies included in this review are not necessarily applicable to infants and children globally. Follow-up periods were often of necessity short, mostly to less than 12 months of age for BCG and to less than 9 months of age for DTP. Many of the studies took place in communities with many years of use of these vaccines. In these studies, a combination of direct vaccine effects and herd immunity gave rise to low incidences of the diseases targeted by the vaccines, so that net benefits of routine use of these vaccines may not have been apparent. One large study, however, observed an increase in mortality on first introduction of the DTP vaccine.^27^ Several studies of MCV also provide results for mortality with censoring for deaths caused by, or as a consequence of, measles infection.^28^ ^29^ ^30^ ^31^ ^32^ ^33^ They reported similar reductions in mortality for these “non-specific” effects to those that we have presented for overall mortality. This suggests that if the effects we observed are real then they are not fully explained by deaths that were established as due to measles.

It is more than 30 years since early observational studies in west Africa suggested that some routine infant immunisations might have effects on risk of mortality and morbidity unrelated to the specific diseases they are intended to prevent.^34^ Our review shows that many studies examining these non-specific effects of various vaccines have now been conducted and provides support for the hypothesis. For example, tuberculosis is now an infrequent cause of death in infants and young children, so if BCG has an effect on all cause mortality it is unlikely to be entirely due to fewer deaths from that disease. On the basis of the few studies that attempted to remove measles deaths from the calculations, any effect of MCV on all cause mortality seems unlikely to be fully accounted for by measles deaths. Any increase in all cause mortality following DTP is also likely to be a non-specific effect

Our review was conducted at the request of WHO following a recommendation of the Strategic Advisory Group of Experts (SAGE) on the need to assess whether the evidence concerning non-specific effects is sufficient to warrant adjusting the routine immunisation schedule or pursuing further research designed to support future evidence based adjustments in immunisation policies. We do not believe that the available evidence supports a change in either the choice of vaccines or the timing or sequence of immunisations routinely administered to infants and children. These views concur with the SAGE recommendations in April 2014.^6^ At the same time, the data raise sufficient concerns for us to strongly recommend further studies on the possible effects of immunisations on the immune system and on the risk of morbidity and mortality, particularly in relation to DTP. Randomised trials are needed to overcome the difficulties of interpretation of observational studies, and they should be sufficiently powered to examine possibly differential effects between boys and girls. Until the results of such trials are available, detrimental non-specific effects of DTP, if any, can probably be minimised by ensuring that infants receive their routine immunisations according to the currently recommended WHO schedule.

**What is already known on this topic**

Vaccines such as those against measles, diphtheria-tetanus-pertussis (DTP), and polio have produced extraordinary reductions in the diseases they target
Some routinely administered vaccines are proposed to have non-specific effects on mortality from conditions other than the infectious diseases they are designed to prevent

**What this study adds**

A comprehensive systematic review and risk of bias assessment found few randomised trials and determined that many types of bias may have influenced the results of the many observational studies
Receipt of BCG and measles containing vaccines may reduce overall mortality by more than expected through their effects on the diseases they prevent, and receipt of DTP may be associated with higher all cause mortality
The evidence does not support a change to existing vaccination recommendations but does indicate a need for randomised trials to examine the positioning of DTP in the vaccine schedule
Until the results of such studies are available, every effort should be made to ensure that infants receive routine immunisations on schedule and in the sequence recommended by WHO.

## Web Extra

Extra material supplied by the author

## Notes

We are indebted to Ana Maria Henao-Restrepo and Ximena Riveros de Laurie (Department of Immunization Vaccines and Biologicals, WHO) for their support throughout the project and to all members of the SAGE Working Group on non-specific effects of vaccines for their input. Nicola Low, Pippa Scott, and Matthias Egger wrote the initial draft of the protocol but did not contribute further to the review. We are grateful to Jelena Savović for help with designing data extraction forms. We thank Michelle Beam, Emi Han, Emma Smith, and Paul Zhang for screening of title and abstracts; Maria Christou for screening of full text articles; and Molly Grimes for copy editing. Contributions of NKM and HC are under the terms of postdoctoral research training fellowships issued by the UK National Institute for Health Research (NIHR); the views expressed in this publication are those of the authors and not necessarily those of the NHS, NIHR, or Department of Health.

Contributors: ALR, KSW, and JPTH were invited to lead the review by WHO staff, on the basis of an existing draft protocol. ALR, KSW, and JPTH revised the protocol. KSW, AK, and ALR selected articles for inclusion. AK, KSW, and JLL extracted data. JLL and JPTH selected results for inclusion. JPTH, KC, HC, and NKM assessed risk of bias. JPTH and JLL did the analyses. JPTH, KSW, and ALR contributed substantially to interpretation of results. KSW and JPTH prepared tables and figures. JPTH wrote the first draft of the paper, and all authors revised it critically for important intellectual content. All authors have approved this version for publication. JPTH is the guarantor.

Ethical approval: Not needed.

Transparency declaration: The lead author affirms that the manuscript is an honest, accurate, and transparent account of the study being reported; that no important aspects of the study have been omitted; and that any discrepancies from the study as planned (and, if relevant, registered) have been explained.

Data sharing: No additional data available.
