# Supplementary material for: Association of BCG, DTP, and measles containing vaccines with childhood mortality: systematic review
Source: BMJ. 2016 Oct 13;355:i5170. doi: 10.1136/bmj.i5170 (PMC5063034; doi:10.1136/bmj.i5170)
Supplement: Supplementary file 1 — Appendix 1-6 [file higj028142.ww1_default.pdf]

Appendix 1: Search strategy

Searches were performed by the World Health Organization information specialists, in collaboration with researchers with expertise in the field from the WHO Initiative for Vaccine Research department. No language restrictions were imposed.

Embase and Medline (Elsevier www.embase.com interface)

Searched from 1966 to 27 November 2012
No age, human or year limits were applied
Details of the search strategy that was used are provided in Table 1

Table 1: Search strategy to identify studies in Embase

#	Searches	Results
1	'child'/exp OR 'child' OR 'children'/exp OR 'children' OR 'youth'/exp OR 'youth' OR youth* OR newborn* OR 'newborn'/exp OR 'newborn' OR 'new born' OR 'childhood disease'/exp OR 'childhood disease' OR 'baby'/exp OR 'baby' OR babies OR 'infant'/exp OR 'infant' OR infant* OR childhood* OR toddler* OR kid OR kids OR 'young patient' OR boy* OR girl* OR 'young age' OR pediatr* OR paediatr* OR 'child death'/exp OR 'child death' OR 'child health'/exp OR 'child health' OR 'child care'/exp OR 'child care' OR 'childhood mortality'/exp OR 'childhood mortality' OR 'child hospitalization'/exp OR 'child hospitalization' OR 'pediatric hospital'/exp OR 'pediatric hospital' OR child*	3,751,199
2	'measles vaccine'/exp OR 'anti measles vaccin' OR ' attenuated live measles vaccine' OR ' attenuated live rubeola virus vaccine' OR ' attenuated measles vaccine' OR ' attenuvax' OR ' cam-kovac' OR ' diplovax' OR ' edmonston zagreb vaccine' OR ' fibroblast grown measles vaccine' OR ' formalin killed measles vaccine' OR ' hyperimmune measles serum' OR ' killed measles vaccine' OR ' killed measles virus vaccine' OR ' lirugen' OR ' lirugen measles' OR ' live attenuated measles vaccine' OR ' live attenuated measles vaccine moraten strain' OR ' live attenuated measles vaccine schwarz strain' OR ' live attenuated measles virus vaccine' OR ' live distemper vaccine' OR ' live distemper virus vaccine' OR ' live measles vaccine' OR ' live rubeola virus vaccine' OR ' m-vac' OR ' measle vaccine' OR ' measles killed vaccine' OR ' measles killed virus vaccine' OR ' measles live vaccine' OR ' measles vaccine, chick embryo fibroblast grown' OR ' measles vaccine, human diploid cell grown' OR ' measles vaccine, tween ether' OR ' measles virus vaccine' OR ' measles virus vaccine live' OR ' mevilin-l' OR ' morbilli vaccine' OR ' morbilvax' OR ' rimevax' OR ' rouvax' OR ' rubeola vaccine' OR ' rubeovax' OR ' tween ether measles vaccine' OR ' vaccine, measles' OR ' vaccine, rubeola' OR ' jeryl lynn moraten vaccine' OR ' mm vax' OR ' mmvax' OR ' mumps measles vaccine' OR ' rimparix' OR ' urabe am 9 rimparix' OR ' urabe am 9 schwarz vaccine' OR 'chickenpox measles mumps rubella vaccine'/exp OR 'measles mumps rubella varicella vaccine' OR ' measles plus mumps plus rubella plus varicella vaccine live ' OR ' proquad'	9,439
3	'BCG vaccine'/exp OR 'antituberculosis vaccine' OR ' b.c.g.' OR ' b.c.g. vaccine' OR ' bacillus Calmette Guerin' OR ' bacillus calmette guerin vaccine' OR ' BCG' OR ' bcg cell wall vaccine' OR ' bcg copenhagen 1331' OR ' BCG live' OR ' bcg test' OR ' calgevax' OR ' calmette guerin bacillus' OR ' calmette vaccine' OR ' calmette s vaccine' OR 'calmettes vaccine' OR ' immucyst' OR ' immun bcg pasteur' OR ' monovax' OR ' mva 85a' OR ' mva85a' OR ' mycobacterium bcg' OR ' mycobax' OR ' onco tice' OR ' oncotice' OR ' pacis' OR ' pastimmun' OR ' theracys intravesical' OR ' tice bcg' OR ' tice bcg vaccine' OR ' ticebcg' OR ' tubercle bacilli vaccine' OR ' tuberculosis vaccine' OR ' tuberculosis vaccines'	39,365
4	'diphtheria pertussis tetanus vaccine'/exp OR 'absorbed pertussis diphtheria tetanus vaccine' OR ' acel immune' OR ' acel imune' OR ' acelluvax dpt' OR ' acelluvax DTP' OR ' adacel' OR ' adsorbed dt coq' OR ' anatoxal di te per berna' OR ' boostrix' OR ' certiva' OR ' covaxis' OR ' d.t. coq' OR ' daptacel' OR ' dif per tet all' OR ' diphtheria-tetanus-acellular pertussis vaccines' OR ' diphtheria-tetanus-pertussis vaccine' OR ' diphtheria plus pertussis plus tetanus' OR ' diphtheria tetanus acellular pertussis vaccines' OR ' diphtheria tetanus pertussis trivaccine' OR ' diphtheria tetanus pertussis vaccine' OR ' diphtheria tetanus whooping cough vaccine' OR ' diteper anatoxal berna vaccine' OR ' dpt' OR ' DPT vaccine' OR ' DTAP vaccine' OR ' DTP vaccine' OR ' infanrix' OR ' neodiftepertus' OR ' p.d.t. vax purified' OR ' pertugen' OR ' pertussis diphtheria tetanus vaccine' OR ' tetanus diphtheria pertussis vaccine' OR ' tri immunol' OR ' triacelluvax' OR ' triaxis' OR ' tripacel' OR ' tripedia' OR ' triplo vaccine' OR ' tripvac' OR ' tritanrix' OR ' trivax' OR ' vaccine, pertussis diphtheria tetanus' OR *'DTP vaccine' OR 'Infanrix'*	11,728
5	'sex difference'/exp OR 'sex differences' OR ' dimorphism, sex' OR ' factor, sex' OR ' gender difference' OR ' gender differences' ' sex dimorphism' OR ' sex factor' OR ' sex factors' OR ' sexual difference' OR ' sexual dimorphism' OR ' sexual size dimorphism' OR 'evolution, sex' OR ' sex characteristics' OR ' sex development' OR ' sex evolution' OR 'advanced puberty' OR ' pseudopuberty' OR ' pubescence' OR 'sex differentiation'/exp OR 'sex related factors' OR 'sex related factor' OR 'sexual development'/exp OR 'male'/exp AND 'female'/exp AND 'gender'/exp OR 'boy'/exp AND 'girl'/exp AND 'gender'/exp	3,668
6	Activins OR 'cytokine'/exp OR activin OR 'adipocytokines' OR ' adipokine' OR ' adipokines' OR ' adipose tissue derived cytokine' OR 'Acrp 30' OR ' Acrp30' OR ' adipocyte complement related protein 30' OR ' adipocyte most abundant protein 1' OR ' adipoq' OR ' APM 1' OR ' APM1' OR ' GBP 28' OR ' GBP28' OR ' gelatin binding protein 28' OR 'AIF 1' OR ' AIF1' OR ' cytokine AIF 1' OR ' cytokine AIF1' OR ' daintain' OR 'a proliferation inducing ligand' OR ' a proliferation inducing ligand protein' OR ' antigen CD256' OR ' CD256 antigen' OR ' protein APRIL' OR ' protein TALL2' OR ' protein TNFSF 13' OR ' protein TNFSF13' OR ' TALL 2 protein' OR ' TALL2 protein' OR ' TNF and ApoL related leukocyte expressed ligand 2' OR ' TNF related death ligand 1' OR ' TNFSF 13 protein' OR ' TNFSF13 protein' OR ' tumor necrosis factor and apolipoprotein related leukocyte expressed ligand 2' OR ' tumor necrosis factor ligand superfamily member 13' OR ' tumor necrosis factor related death ligand 1' OR ' tumor necrosis factor SF13' OR ' tumor necrosis factor superfamily member 13' OR 'B lymphocyte activating factor [134-285]' OR ' B lymphocyte stimulator [134-285]' OR 'ATX protein' OR ' ectonucleotide pyrophosphatase phosphodiesterase 2' OR ' ENPP2 protein' OR ' PDNP2 protein' OR ' protein ATX' OR ' protein ENPP2' OR ' protein PDNP2' OR 'antigen CD257' OR ' B-cell activating factor' OR ' B cell activation factor' OR ' B lymphocyte activating factor' OR ' B lymphocyte stimulator' OR ' B lymphocyte stimulator protein' OR ' BAFF' OR ' BLyS protein' OR '	1,387,342

#	Searches	Results
	CD257 antigen’ OR ‘ protein BLyS’ OR ‘ protein TALL 1’ OR ‘ protein TALL1’ OR ‘ protein TNFSF13B’ OR ‘ TALL 1 protein’ OR ‘ TALL1 protein’ OR ‘ TNF and ApoL related leukocyte expressed ligand 1’ OR ‘ TNFSF13B protein’ OR ‘ tumor necrosis factor and apolipoprotein related leukocyte expressed ligand 1’ OR ‘ tumor necrosis factor ligand superfamily member 13B’ OR ‘B cell differentiation factor’ OR ‘ bcdf’ OR ‘B cell growth factor’ OR ‘ bcgf’ OR ‘ growth factor, b cell’ OR ‘bone morphogenetic proteins’ OR ‘ bone morphogenic protein’ OR ‘BMP 12’ OR ‘ BMP12’ OR ‘ cartilage derived morphogenetic protein 3’ OR ‘ CDMP 3’ OR ‘ CDMP3’ OR ‘ GDF 7’ OR ‘ GDF7’ OR ‘ growth and differentiation factor 7’ OR ‘ growth differentiation factor 7’ OR ‘BMP 15’ OR ‘ BMP15’ OR ‘ GDF 9B’ OR ‘ GDF9B’ OR ‘ growth and differentiation factor 9B’ OR ‘ growth differentiation factor 9B’ OR ‘BMP 2’ OR ‘ BMP2’ OR ‘BMP 4’ OR ‘ BMP4’ OR ‘BMP 5’ OR ‘ BMP5’ OR ‘BMP 6’ OR ‘ BMP6’ OR ‘BMP 9’ OR ‘ BMP9’ OR ‘ GDF 2’ OR ‘ GDF2’ OR ‘ growth and differentiation factor 2’ OR ‘ growth differentiation factor 2’ OR ‘4-1BB ligand’ OR ‘ 4 1BB ligand’ OR ‘ 4 1BBL protein’ OR ‘ CD137L’ OR ‘ ligand 4 1BB’ OR ‘ protein 4 1BBL’ OR ‘antigen CD153’ OR ‘ CD153 antigen’ OR ‘ CD153 antigens’ OR ‘ CD30L’ OR ‘ protein TNFSF 8’ OR ‘ protein TNFSF8’ OR ‘ TNFSF 8 protein’ OR ‘ TNFSF8 protein’ OR ‘ tumor necrosis factor ligand superfamily member 8’ OR ‘ tumor necrosis factor superfamily member 8’ OR ‘antigen CD154’ OR ‘ CD154 antigen’ OR ‘ CD40L’ OR ‘ CD40L antigen’ OR ‘ protein TNFSF 5’ OR ‘ protein TNFSF5’ OR ‘ TNFSF 5 protein’ OR ‘ TNFSF5 protein’ OR ‘ tumor necrosis factor ligand superfamily member 5’ OR ‘ tumor necrosis factor superfamily member 5’ OR ‘antigen cd70’ OR ‘ antigens, CD70’ OR ‘ CD27 ligand’ OR ‘ CD27L’ OR ‘ CD70 antigens’ OR ‘colony-stimulating factors’ OR ‘ colony stimulating activity’ OR ‘ colony stimulating factors’ OR ‘fibroblast derived differentiation inducing factor’ OR ‘ectodermal dysplasia protein’ OR ‘ ectodysplasin 1’ OR ‘ ectodysplasins’ OR ‘ EDA A protein’ OR ‘ EDA protein’ OR ‘ protein EDA’ OR ‘ protein EDA A’ OR ‘am 424’ OR ‘ am424’ OR ‘ recombinant human leukemia inhibitory factor’ OR ‘ recombinant leukemia inhibitory factor’ OR ‘EMAP II’ OR ‘ endothelial monocyte activating polypeptide 2’ OR ‘antigen CD178’ OR ‘ CD178 antigen’ OR ‘ CD95 ligand’ OR ‘ CD95L’ OR ‘ CD95L protein’ OR ‘ Fas antigen ligand’ OR ‘ Fas ligand protein’ OR ‘ FasL protein’ OR ‘ protein CD95L’ OR ‘ protein FasL’ OR ‘ protein TNFSF 6’ OR ‘ protein TNFSF6’ OR ‘ TNF superfamily member 6’ OR ‘ TNFSF 6 protein’ OR ‘ TNFSF6 protein’ OR ‘ tumor necrosis factor ligand superfamily member 6’ OR ‘fibroblast growth factors’ OR ‘ fibroblast stimulating factor’ OR ‘ heparin binding growth factor’ OR ‘fgf 1’ OR ‘ FGF1’ OR ‘fgf 10’ OR ‘ FGF10’ OR ‘ fgf 14’ OR ‘ FGF14’ OR ‘ fgf 16’ OR ‘ FGF16’ OR ‘ fgf 18’ OR ‘ FGF18’ OR ‘ fgf 19’ OR ‘ FGF19’ OR ‘ fgf2’ OR ‘ FGF 2’ OR ‘ fgf21’ OR ‘ FGF 21’ OR ‘ fgf23’ OR ‘ FGF 23’ OR ‘ fgf3’ OR ‘ FGF 3’ OR ‘ fgf4’ OR ‘ FGF 4’ OR ‘ fgf5’ OR ‘ FGF 5’ OR ‘ fgf6’ OR ‘ FGF 6’ OR ‘ fgf8’ OR ‘ FGF 8’ OR ‘ fgf9’ OR ‘ FGF 9’ OR ‘interleukin’ OR ‘interleukins’ OR ‘il 1’ OR ‘il 2’ OR ‘il 4’ OR ‘il 5’ OR ‘il 6’ OR ‘il 9’ OR ‘il 10’ OR ‘il 12’ OR ‘il 13’ OR ‘il 17’ OR ‘il 23’ OR ‘interferon’ OR ‘helper cell type 1’ OR ‘ T helper 1’ OR ‘ T helper type 1’ OR ‘ Th1 cells’ OR ‘helper cell type 2’ OR ‘ T helper 2’ OR ‘ T helper type 2’ OR ‘ Th2 cells’ OR 'helper cell'/exp OR 't helper' OR 'B lymphocyte'/exp OR ‘B-lymphocyte subsets’ OR ‘ B-lymphocytes’ OR ‘ b-lymphocytes, regulatory’ OR ‘ B cell’ OR ‘ bone marrow derived lymphocyte’ OR ‘ bone marrow lymphocyte’ OR ‘ bursa derived lymphocyte’ OR ‘ lymphocyte, b’ OR ‘ lymphocyte, bone marrow derived’ OR ‘ lymphocyte, bursa derived’ OR ‘ regulatory B lymphocyte’ OR ‘antibody-producing cells’ OR ‘ antibody forming cell’ OR ‘ antibody producing cell’ OR ‘ immunoglobulin forming cell’ OR ‘B memory cell’ OR ‘ B memory cells’ OR ‘ B memory lymphocyte’ OR ‘ B memory lymphocytes’ OR ‘ memory B cell’ OR ‘ memory B cells’ OR ‘ memory B lymphocyte’ OR ‘ memory B lymphocytes’ OR ‘cell, plasma’ OR ‘ flamed plasma cell’ OR ‘ flamed plasmacell’ OR ‘ plasma cells’ OR ‘ plasmacyte’ OR ‘ plasmatocyte’ OR ‘ plasmocyte’ OR ‘ plasmocyte, flamed’ OR ‘B cell precursor’ OR ‘ B cell precursors’ OR ‘ B cell progenitor’ OR ‘ B cell progenitors’ OR ‘ B lineage precursor’ OR ‘ B lineage precursors’ OR ‘ B lineage progenitor’ OR ‘ B lineage progenitors’ OR ‘ B lymphocyte precursor’ OR ‘ B lymphocyte progenitor’ OR ‘ B lymphocyte progenitors’ OR ‘ B lymphoid precursor cell’ OR ‘ B lymphoid precursor cells’ OR ‘ B lymphoid progenitor’ OR ‘ B lymphoid progenitors’ OR ‘ B precursor’ OR ‘ B precursors’ OR ‘ B progenitor’ OR ‘ B progenitors’ OR ‘ cell, pre B’ OR ‘ immature B cell’ OR ‘ immature B cells’ OR ‘ pre B cell’ OR ‘ pre B cells’ OR ‘ precursor B cell’ OR ‘ precursor B cells’ OR ‘ precursor B lymphocyte’ OR ‘ precursor B lymphocytes’ OR ‘ precursor cells, B-lymphoid’ OR ‘ precursor cells, B lymphoid’ OR ‘ pro-B cell’ OR ‘ pro-B cells’ OR ‘ progenitor B cell’ OR ‘ progenitor B cells’ OR ‘ transitional B cell’ OR ‘ transitional B cells’ OR " tumor necrosis factor receptor 1"/exp OR " tumor necrosis factor receptor 1 " OR " CD120a antigen" OR " receptors, tumor necrosis factor, type I" OR " tumor necrosis factor receptor type 1" OR " tumor necrosis factor receptor type I" OR 'dendritic cell'/exp OR ‘dendritic cells’ OR ‘dendritic cell’ OR 'langerhans cell' OR 'langerhans cells' OR 'T lymphocyte'/exp OR ‘amplifier t lymphocyte’ OR ‘ lymphocyte, thymus’ OR ‘ T-lymphocytes’ OR ‘ t-lymphocytes, suppressor-inducer’ OR ‘ T cell’ OR ‘ T cells’ OR ‘ thymic lymphocyte’ OR ‘ thymus dependant lymphocyte’ OR ‘ thymus dependent cell’ OR ‘ thymus dependent lymphocyte’ OR ‘ thymus derived cell’ OR ‘ thymus derived lymphocyte’ OR ‘ thymus lymphocyte’	
7	'retinol'/exp OR ‘3, 7 dimethyl 9 (2, 6, 6 trimethyl 1 cyclohexen 1 yl) 2, 4, 6, 8 nonatetraen 1 ol’ OR ‘ 3, 7 dimethyl 9 (2, 6, 6 trimethyl 1 cyclohexenyl) 2, 4, 6, 8 nonatetraen 1 ol’ OR ‘ a 313’ OR ‘ a fil’ OR ‘ a mulsal’ OR ‘ a mulsin’ OR ‘ a mulsine’ OR ‘ a sol’ OR ‘ a vi pel’ OR ‘ a vit’ OR ‘ a vitadit’ OR ‘ a vitamin’ OR ‘ a vitan’ OR ‘ a313’ OR ‘ acon’ OR ‘ acrisina’ OR ‘ acrisine’ OR ‘ actifral a’ OR ‘ adatone’ OR ‘ afaxin’ OR ‘ afaxine’ OR ‘ afilina’ OR ‘ afiline’ OR ‘ agiolan’ OR ‘ alcovit a’ OR ‘ alfa monovite’ OR ‘ alfa sir’ OR ‘ alfaergin’ OR ‘ alfaergine’ OR ‘ alfamin’ OR ‘ alfamine’ OR ‘ alfamonovit’ OR ‘ alfasir’ OR ‘ alfasole’ OR ‘ alfasterolo’ OR ‘ alfatar’ OR ‘ alfavena’ OR ‘ alfavene’ OR ‘ alfavitina’ OR ‘ alfavitine’ OR ‘ alfene’ OR ‘ all trans retinol’ OR ‘ alphalin’ OR ‘ alphaline’ OR ‘ alphasterol’ OR ‘ amulsal’ OR ‘ amulsin’ OR ‘ amulsine’ OR ‘ amulvit’ OR ‘ anatola’ OR ‘ anatole’ OR ‘ anavit’ OR ‘ anti infective vitamin’ OR ‘ antixerophthalmic vitamin’ OR ‘ aoral’ OR ‘ apexol’ OR ‘ apostavit’ OR ‘ aquasol a’ OR ‘ arcavit A’ OR ‘ asol’ OR ‘ asteril’ OR ‘ atav’ OR ‘ aterapion’ OR ‘ avibon’ OR ‘ avibon theraplix’ OR ‘ avimin’ OR ‘ avimine’ OR ‘ avipel’ OR ‘ avipur’ OR ‘ avit’ OR ‘ avitabiol’ OR ‘ avitadit’ OR ‘ avital’ OR ‘ avitaminum kolin’ OR ‘ avitan’ OR ‘ avitana’ OR ‘ avitane’ OR ‘ avite’ OR ‘ avitil’ OR ‘ avitina’ OR ‘ avitol’ OR ‘ avogina’ OR ‘ avogine’ OR ‘ avoleum’ OR ‘ axerodina’ OR ‘ axerodine’ OR ‘ axerol’ OR ‘ axerophthol’ OR ‘ axerophthylium’ OR ‘ bentavit a’ OR ‘ bentavite a’ OR ‘ bio tan’ OR ‘ biosterol’ OR ‘ biotan’ OR ‘ chivibit a’ OR ‘ cytobiase’ OR ‘ dagravit a’ OR ‘ davitamon a’ OR ‘ difvitamin a’ OR ‘ dohyfral a’ OR ‘ elageno a’ OR ‘ endo a’ OR ‘ envit a’ OR ‘ epiteliol’ OR ‘ evitol zambeletti’ OR ‘ fletase’ OR ‘ gadeol’ OR ‘ gadol’ OR ‘ halivitan’ OR ‘ halivitane’ OR ‘ homagenets aoral’ OR ‘ hydrosol’ OR ‘ ido a’ OR ‘ ido a 50’ OR ‘ idratene’ OR ‘ inovitan a’ OR ‘ lord factor’ OR ‘ meditalfa’ OR ‘ mulsal a’ OR ‘ multamine’ OR ‘ nio a let’ OR ‘ oleovit a’ OR ‘ oleovitamin a’ OR ‘ ophthalamin’ OR ‘ panvita’ OR ‘ plivit a’ OR ‘ prepalin’ OR ‘ prepaline’ OR ‘ preparato a’ OR ‘ primavit’ OR ‘ quotivit’ OR ‘ retinol alcohol’ OR ‘ retinyl alcohol’ OR ‘ ro-a-vit’ OR ‘ ro a vit’ OR ‘ super a’ OR ‘ testavol’ OR ‘ ucemine a’ OR ‘ vaconex’ OR ‘ vaflol’ OR ‘ veroftal’ OR ‘ vi alpha’ OR ‘ vi dom a’ OR ‘ viadenin’ OR ‘ vialpha’ OR ‘ viatate’ OR ‘ vidoma’ OR ‘ vitadone’ OR ‘ vitadral’ OR ‘ vitalen a’ OR ‘ vitalfa’ OR ‘ vitama’ OR ‘ vitamin A’ OR ‘ vitamin a alcohol’ OR ‘ vitamin a1’ OR ‘ vitaplex a’ OR ‘ vitapur a’ OR ‘ vitasan a’ OR ‘ vitavel a’ OR ‘ vitpex’ OR ‘ vogan’ OR ‘ vogan neu’ OR ‘ wandervit a’ OR ‘ xerophthol’ OR ‘vitamin A’	56,112
8	Death OR deaths OR died OR mortality OR 'mortality'/exp OR mortalities OR fatal OR dying OR deceased OR “life threatening” OR “severe reaction” OR ICU OR “intensive care” OR emergency OR urgent OR fatalities OR casualty OR casualties OR lethalness OR necrosis OR heterologous OR ‘non specific’ OR nonspecific OR ‘non-specific’	3,369,761
	'tetanus toxoid'/exp OR ‘anatetall’ OR ‘ anatoxal tetanica berna’ OR ‘ anti tetanus toxoid’ OR ‘ antitetanus toxoid’ OR ‘ antitetanus vaccine’ OR ‘ clostet’ OR ‘ plain tetanus vaccine’ OR ‘ tanrix’ OR ‘ te anatoxal’ OR ‘ te anatoxal berna’ OR ‘ tet-tox’ OR ‘ tetanol’ OR ‘ tetanus anatoxin’ OR ‘ tetanus toxoid fluid’ OR ‘ tetanus toxoid vaccine’ OR ‘ tetanus vaccine’ OR ‘ tetatox’ OR ‘ tetatoxoid’ OR ‘ tetavax’ OR ‘tetanus toxoid’ OR ‘57425-69-1’ OR ‘ 93384-51-1’	18,380
10	'pertussis vaccine'/exp OR ‘acellular pertussis vaccine’ OR ‘ acelluvax’ OR ‘ bordetella pertussis vaccin’ OR ‘ Bordetella	7,746

#	Searches	Results
	pertussis vaccine' OR ' jnih 6' OR ' pertussis acellular vaccine' OR ' pertussis vaccin' OR ' pertuvac' OR ' whooping cough vaccine'	
11	Step 1 AND Step 3 AND Step 5	225
12	Step 1 AND Step 3 AND Step 8	
13	Step 1 AND Step 2 AND Step 7	100
14	Step 1 AND Step 4 AND Step 5	167
15	Step 1 AND Step 4 AND Step 8	896
16	Step 1 AND Step 4 AND Step 7	137
17	Step 1 AND Step 4 AND Step 6	2536
18	Step 1 AND Step 2 AND Step 5	210
19	Step 1 AND Step 2 AND Step 8	1751
20	Step 1 AND Step 4 AND Step 8	1823
21	Step 1 AND Step 4 AND Step 7	206
22	Step 4 AND Step 6	1268
23	Step 10 AND Step 6	95
24	Step 9 AND Step 6 NOT (Animal/exp NOT Human/exp)	2,811
25	Step 3 AND Step 6 NOT ((Animal/exp NOT Human/exp)) NOT neoplasm/exp	4,855

Global Index Medicus (www.globalhealthlibrary.net)

Searched on 11 March 2013

No age, human or year limits were applied

The Global Index Medicus provides worldwide access to biomedical and public health literature produced by and within low- and middle- income countries. The databases searched through the Global Index Medicus were: African Index Medicus (AIM, 1964-2013), Latin American and Caribbean Health Sciences (LILACs, 1980-2013), African Index Medicus, Eastern Mediterranean Index Medicus (IMEAR, 1966-2013), South-East Asia (IMSEAR 1947-2013), Western Pacific (WPRIM, 1964-2013). Details of the search strategy that was used are provided in Table 2.

Table 2: Search strategy for the Global Index Medicus

#	Searches	Results
**1**	((child) OR (child) OR (children) OR (children) OR (youth) OR (youth) OR youth* OR newborn* OR (newborn) OR (newborn) OR (new born) OR (childhood disease) OR (childhood disease) OR (baby) OR (baby) OR babies OR (infant) OR (infant) OR infant* OR childhood* OR toddler* OR kid OR kids OR (young patient) OR boy* OR girl* OR (young age) OR pediatr* OR paediatr* OR (child death) OR (child death) OR (child health) OR (child health) OR (child care) OR (child care) OR (childhood mortality) OR (childhood mortality) OR (child hospitalization) OR (child hospitalization) OR (pediatric hospital) OR (pediatric hospital) OR child*)	3,751,199
**2**	((measles vaccine) OR (anti measles vaccin) OR (attenuated live measles vaccine) OR (attenuated live rubeola virus vaccine) OR (attenuated measles vaccine) OR (attenuvax) OR (cam kovac) OR (diplovax) OR (edmonston zagreb vaccine) OR (fibroblast grown measles vaccine) OR (formalin killed measles vaccine) OR (hyperimmune measles serum) OR (killed measles vaccine) OR (killed measles virus vaccine) OR (lirugen) OR (lirugen measles) OR (live attenuated measles vaccine) OR (live attenuated measles vaccine moraten strain) OR (live attenuated measles vaccine schwarz strain) OR (live attenuated measles virus vaccine) OR (live distemper vaccine) OR (live distemper virus vaccine) OR (live measles vaccine) OR (live rubeola virus vaccine) OR (m vac) OR (measle vaccine) OR (measles killed vaccine) OR (measles killed virus vaccine) OR (measles live vaccine) OR (measles virus vaccine) OR (measles virus vaccine live) OR (mevilin l) OR (morbilli vaccine) OR (morbilvax) OR (rimevax) OR (rouvax) OR (rubeola vaccine) OR (rubeovax) OR (tween ether measles vaccine) OR (jeryl lynn moraten vaccine) OR (mm vax) OR (mmvax) OR (rimparix) OR (urabe am 9 rimparix) OR (urabe am 9 schwarz vaccine) OR (chickenpox measles mumps rubella vaccine) OR (measles mumps rubella varicella vaccine) OR (measles plus mumps plus rubella plus varicella vaccine live) OR (proquad))	9,439
**3**	((BCG) OR (antituberculosis vaccine) OR Calmette OR Guerin OR (copenhagen 1331) OR (BCG live) OR calgevax OR calmettes OR immucyst OR monovax OR mva OR 85a OR mva85a OR mycobax OR onco OR tice OR oncotice OR pacis OR pastimmun OR (theracys intravesical) OR (ticebcg) OR (tubercle bacilli vaccine) OR (tuberculosis vaccine) OR (tuberculosis vaccines))	39,365
**4**	((tetanus vaccine) OR (acel immune) OR (acel imune) OR (acelluvax dpt) OR (adacel) OR (adsorbed dt coq) OR (anatoxal di te per berna) OR boostrix OR certiva OR covaxis OR daptacel OR (dif per tet all) OR (pertussis vaccines) OR (diphtheria plus pertussis plus tetanus) OR (diphtheria tetanus acellular pertussis vaccines) OR (diphtheria tetanus pertussis trivaccine) OR (diphtheria tetanus pertussis vaccine) OR (diphtheria tetanus whooping cough vaccine) OR (diteper anatoxal berna vaccine) OR dpt OR (DPT vaccine) OR (DTAP vaccine) OR (DTP vaccine) OR infanrix OR neodiftepertus OR (vax purified) OR pertugen OR (pertussis diphtheria tetanus vaccine) OR (tetanus diphtheria pertussis vaccine) OR (tri immunol) OR triacelluvax OR triaxis OR tripacel OR tripedia OR (triplo vaccine) OR tripvac OR tritanrix OR trivax OR *(DTP vaccine) OR Infanrix)*	11,728
**5**	((sex difference) OR (sex differences) OR (dimorphism) OR (gender difference) OR (gender differences) OR (sex dimorphism) OR (sex factor) OR (sex factors) OR (sexual difference) OR (sexual dimorphism) OR (sexual size dimorphism) OR (sex characteristics) OR (sex development) OR (sex evolution) OR (advanced puberty) OR (pseudopuberty) OR (pubescence) OR (sex differentiation) OR (sex related factors) OR (sex related factor) OR (sexual development) OR (male AND female AND gender) OR (boy AND girl AND gender))	3,668

#	Searches	Results
**6**	(Activins OR (cytokine) OR activin OR (adipocytokines) OR (adipokine) OR (adipokines) OR (adipose tissue derived cytokine) OR (Acrp 30) OR (Acrp30) OR (adipocyte complement related protein 30) OR (adipocyte most abundant protein 1) OR (adipoq) OR (APM 1) OR (APM1) OR (GBP 28) OR (GBP28) OR (gelatin binding protein 28) OR (AIF 1) OR (AIF1) OR (cytokine AIF 1) OR (cytokine AIF1) OR (daintain) OR (antigen CD256) OR (CD256 antigen) OR (protein APRIL) OR (protein TALL2) OR (protein TNFSF 13) OR (protein TNFSF13) OR (TALL 2 protein) OR (TALL2 protein) OR (TNF and ApoL related leukocyte expressed ligand 2) OR (TNF related death ligand 1) OR (TNFSF 13 protein) OR (TNFSF13 protein) OR (tumor necrosis factor and apolipoprotein related leukocyte expressed ligand 2) OR (tumor necrosis factor ligand superfamily member 13) OR (tumor necrosis factor related death ligand 1) OR (tumor necrosis factor SF13) OR (tumor necrosis factor superfamily member 13) OR (B lymphocyte activating factor) OR (ATX protein) OR (ectonucleotide pyrophosphatase phosphodiesterase 2) OR (ENPP2 protein) OR (PDNP2 protein) OR (protein ATX) OR (protein ENPP2) OR (protein PDNP2) OR (antigen CD257) OR (B-cell activating factor) OR (B cell activation factor) OR (B lymphocyte activating factor) OR (B lymphocyte stimulator) OR (B lymphocyte stimulator protein) OR (BAFF) OR (BLyS protein) OR (CD257 antigen) OR (protein BLyS) OR (protein TALL 1) OR (protein TALL1) OR (protein TNFSF13B) OR (TALL 1 protein) OR (TALL1 protein) OR (TNF and ApoL related leukocyte expressed ligand 1) OR (TNFSF13B protein) OR (tumor necrosis factor and apolipoprotein related leukocyte expressed ligand 1) OR (tumor necrosis factor ligand superfamily member 13B) OR (B cell differentiation factor) OR (bcdf) OR (B cell growth factor) OR (bcgf) OR (bone morphogenetic proteins) OR (bone morphogenic protein) OR (BMP 12) OR (BMP12) OR (cartilage derived morphogenetic protein 3) OR (CDMP 3) OR (CDMP3) OR (GDF 7) OR (GDF7) OR (growth and differentiation factor 7) OR (growth differentiation factor 7) OR (BMP 15) OR (BMP15) OR (GDF 9B) OR (GDF9B) OR (growth and differentiation factor 9B) OR (growth differentiation factor 9B) OR (BMP 2) OR (BMP2) OR (BMP 4) OR (BMP4) OR (BMP 5) OR (BMP5) OR (BMP 6) OR (BMP6) OR (BMP 9) OR (BMP9) OR (GDF 2) OR (GDF2) OR (growth and differentiation factor 2) OR (growth differentiation factor 2) OR (4-1BB ligand) OR (4 1BB ligand) OR (4 1BBL protein) OR (CD137L) OR (ligand 4 1BB) OR (protein 4 1BBL) OR (antigen CD153) OR (CD153 antigen) OR (CD153 antigens) OR (CD30L) OR (protein TNFSF 8) OR (protein TNFSF8) OR (TNFSF 8 protein) OR (TNFSF8 protein) OR (tumor necrosis factor ligand superfamily member 8) OR (tumor necrosis factor superfamily member 8) OR (antigen CD154) OR (CD154 antigen) OR (CD40L) OR (CD40L antigen) OR (protein TNFSF 5) OR (protein TNFSF5) OR (TNFSF 5 protein) OR (TNFSF5 protein) OR (tumor necrosis factor ligand superfamily member 5) OR (tumor necrosis factor superfamily member 5) OR (antigen cd70) OR (CD70) OR (CD27 ligand) OR (CD27L) OR (CD70 antigens) OR (colony-stimulating factors) OR (colony stimulating activity) OR (colony stimulating factors) OR (fibroblast derived differentiation inducing factor) OR (ectodermal dysplasia protein) OR (ectodysplasin 1) OR (ectodysplasins) OR (EDA A protein) OR (EDA protein) OR (protein EDA) OR (protein EDA A) OR (am 424) OR (am424) OR (recombinant human leukemia inhibitory factor) OR (recombinant leukemia inhibitory factor) OR (EMAP II) OR (endothelial monocyte activating polypeptide 2) OR (antigen CD178) OR (CD178 antigen) OR (CD95 ligand) OR (CD95L) OR (CD95L protein) OR (Fas antigen ligand) OR (Fas ligand protein) OR (FasL protein) OR (protein CD95L) OR (protein FasL) OR (protein TNFSF 6) OR (protein TNFSF6) OR (TNF superfamily member 6) OR (TNFSF 6 protein) OR (TNFSF6 protein) OR (tumor necrosis factor ligand superfamily member 6) OR (fibroblast growth factors) OR (fibroblast stimulating factor) OR (heparin binding growth factor) OR (fgf 1) OR (FGF1) OR (fgf 10) OR (FGF10) OR (fgf 14) OR (FGF14) OR (fgf 16) OR (FGF16) OR (fgf 18) OR (FGF18) OR (fgf 19) OR (FGF19) OR (fgf2) OR (FGF 2) OR (fgf21) OR (FGF 21) OR (fgf23) OR (FGF 23) OR (fgf3) OR (FGF 3) OR (fgf4) OR (FGF 4) OR (fgf5) OR (FGF 5) OR (fgf6) OR (FGF 6) OR (fgf8) OR (FGF 8) OR (fgf9) OR (FGF 9) OR (interleukin) OR (interleukins) OR (il 1) OR (il 2) OR (il 4) OR (il 5) OR (il 6) OR (il 9) OR (il 10) OR (il 12) OR (il 13) OR (il 17) OR (il 23) OR (interferon) OR (helper cell type 1) OR (T helper 1) OR (T helper type 1) OR (Th1 cells) OR (helper cell type 2) OR (T helper 2) OR (T helper type 2) OR (Th2 cells) OR (helper cell) OR (t helper) OR (B lymphocyte) OR (B-lymphocyte subsets) OR (B-lymphocytes) OR (lymphocyte) OR (antibody-producing cells) OR (antibody forming cell) OR (antibody producing cell) OR (immunoglobulin forming cell) OR (B memory cell) OR (B memory cells) OR (B memory lymphocyte) OR (B memory lymphocytes) OR (memory B cell) OR (memory B cells) OR (memory B lymphocyte) OR (memory B lymphocytes) OR (flamed plasma cell) OR (flamed plasmacell) OR (plasma cells) OR (plasmacyte) OR (plasmatocyte) OR (plasmocyte) OR (B cell precursor) OR (B cell precursors) OR (B cell progenitor) OR (B cell progenitors) OR (B lineage precursor) OR (B lineage precursors) OR (B lineage progenitor) OR (B lineage progenitors) OR (B lymphocyte precursor) OR (B lymphocyte progenitor) OR (B lymphocyte progenitors) OR (B lymphoid precursor cell) OR (B lymphoid precursor cells) OR (B lymphoid progenitor) OR (B lymphoid progenitors) OR (B precursor) OR (B precursors) OR (B progenitor) OR (B progenitors) OR OR (immature B cell) OR (immature B cells) OR (pre B cell) OR (pre B cells) OR (precursor B cell) OR (precursor B cells) OR (precursor B lymphocyte) OR (precursor B lymphocytes) OR (pro-B cell) OR (pro-B cells) OR (progenitor B cell) OR (progenitor B cells) OR (transitional B cell) OR (transitional B cells) OR (tumor necrosis factor receptor 1) OR (tumor necrosis factor receptor 1) OR (CD120a antigen) OR (dendritic cells) OR (dendritic cell) OR (langerhans cell) OR (langerhans cells) OR (T lymphocyte) OR (amplifier t lymphocyte) OR (T-lymphocytes) OR (T cell) OR (T cells) OR (thymic lymphocyte) OR (thymus dependant lymphocyte) OR (thymus dependent cell) OR (thymus dependent lymphocyte) OR (thymus derived cell) OR (thymus derived lymphocyte) OR (thymus lymphocyte))	1,387,342
**7**	(retinol OR dimethyl OR trimethyl OR cyclohexen OR nonatetraen OR cyclohexenyl OR (nonatetraen) OR (a 313) OR (a fil) OR (a mulsal) OR (a mulsin) OR (a mulsine) OR (a sol) OR (a vi pel) OR (a vit) OR (a vitadit) OR (a vitamin) OR (a vitan) OR a313 OR acon OR acrisina OR (acrisine) OR (actifral a) OR adatone OR afaxin OR afaxine OR afilina OR afiline OR agiolan OR (alcovit a) OR (alfa monovite) OR (alfa sir) OR alfaergin OR alfaergine OR alfamin OR alfamine OR alfamonovit OR alfasir OR alfasole OR alfasterolo OR alfatar OR alfavena OR alfavene OR alfavitina OR alfavitine OR alfene OR alphalin OR alphaline OR alphasterol OR amulsal OR amuls OR amulsine OR amulvit OR anatola OR anatole OR anavit OR (anti infective vitamin) OR (antixerophthalmic vitamin) OR aoral OR apexol OR apostavit OR (aquasol a) OR (arcavit A) OR asol OR asteril OR atav OR aterapion OR avibon OR avibon theraplix OR avimin OR (avimine) OR avipel OR avipur OR avit OR avitabiol OR (avitadit) OR avital OR (avitaminum kolin) OR avitan OR avitana OR avitane OR avite OR avitil OR avitina OR avitol OR avogina OR avogine OR avoleum OR axerodina OR axerodine OR axerol OR (axerophthol) OR axerophthylium OR (bentavit a) OR (bentavite a) OR (bio tan) OR biosterol OR biotan OR (chivibit) OR (cytobiase) OR (dagravit a) OR (davitamon a) OR (difvitamin a) OR (dohyfral a) OR (elageno a) OR (endo a) OR (envit a) OR epiteliol OR (evitol zambeletti) OR fletase OR gadeol OR gadol OR halivitan OR (halivitane) OR (homagenets aoral) OR hydrosol OR (ido a) OR (ido a 50) OR idratene OR (inovitan a) OR (lord factor) OR meditalfa OR (mulsal a) OR multamine OR (nio a	56,112

#	Searches	Results
	let) OR (oleovit a) OR (oleovitamin a) OR ophthalamin OR panvita OR (plivit a) OR prepalin OR prepaline OR (preparato a) OR primavit OR quotivit OR (retinyl alcohol) OR (ro a vit) OR (super a) OR testavol OR (uce mine a) OR vaconex OR vaflol OR veroftal OR (vi alpha) OR (vi dom a) OR viadenin OR vialpha OR viatate OR vidoma OR vitadone OR vitadral OR (vitalen a) OR vitalfa OR vitama OR (vitamin A) OR (vitaplex a) OR (vitapur a) OR (vitasan a) OR (vitavel a) OR vitpex OR vogan OR (vogan neu) OR (wandervit a) OR xerophth ol)	
**8**	(Death OR deaths OR died OR mortality OR mortality OR mortalities OR fatal OR dying OR deceased OR (life threatening) OR (severe reaction) OR ICU OR (intensive care) OR emergency OR urgent OR fatalities OR casualty OR casualties OR lethalness OR necrosis OR heterologous OR (non specific) OR nonspecific)	3,369,761
**9**	((tetanus toxoid) OR anatetall OR (anatoxal tetanica berna) OR (anti tetanus toxoid) OR (antitetanus toxoid) OR (antitetanus vaccine) OR clostet OR (plain tetanus vaccine) OR (tanrix) OR (te anatoxal) OR (tet-tox) OR tetanol OR (tetanus anatoxin) OR (tetanus toxoid fluid) OR (tetanus toxoid vaccine) OR (tetanus vaccine) OR tetatox OR tetatoxoid OR tetavax OR (tetanus toxoid) OR (57425 69 1) OR (93384 51 1))	18,380
**10**	((pertussis vaccine) OR (acellular pertussis vaccine) OR acelluvax OR bordetella pertussis vaccin) OR (Bordetella pertussis vaccine) OR (jnih 6) OR (pertussis acellular vaccine) OR (pertussis vaccin) OR pertuvac OR (whooping cough vaccine))	7,746
**11**	Step 1 AND Step 3 AND Step 5	225
**12**	Step 1 AND Step 3 AND Step 8	
**13**	Step 1 AND Step 2 AND Step 7	100
**14**	Step 1 AND Step 4 AND Step 5	167
**15**	Step 1 AND Step 4 AND Step 8	896
**16**	Step 1 AND Step 4 AND Step 7	137
**17**	Step 1 AND Step 4 AND Step 6	2536
**18**	Step 1 AND Step 2 AND Step 5	210
**19**	Step 1 AND Step 2 AND Step 8	1751
**20**	Step 1 AND Step 4 AND Step 8	
**21**	Step 1 AND Step 4 AND Step 7	206
**22**	Step 4 AND Step 6	
23	Step 10 AND Step 6	95

WHO International Clinical Trials Registry Platform Search Portal

Includes the following databases: ClinicalTrials.gov, International Standard Randomized Controlled Trial Number Register (ISRCTN), and clinical trial registries of Australia, China, Germany, India, Iran, The Netherlands, New Zealand, Sri Lanka), GSK Clinical Study Register, Clinicalstudyresults.org (includes Wyeth trial listings), and listings of the European Medicines Agency (EMEA). Searched in March 2013.
The searches generated 670 records that were manually inspected by two reviewers.

Contact with experts in the field

The following groups of experts were consulted and provided information on published and unpublished relevant research in the field:

- The Bandim Health Project coordinated by Professor Peter Aaby (Copenhagen, Denmark)
- Professor Frank Shann (University of Melbourne, Australia)
- Dr Chris Karp (Bill & Melinda Gates Foundation, USA)
- Dr Mihai Netea (Radboud University of Nijmegen, Holland)

Members of the Department of Immunization, Vaccines and Biologicals of the World Health Organization were asked for any studies (published and unpublished) regarding the potential non-specific effects of vaccines.

A list of 809 references was added to the screening process as a result of consultation with experts.

Appendix 2: Selection of results

We used the following algorithm to select one result from each cohort for the main effects of BCG, DTP and MCV, to avoid double counting of children.

1. Select comparison with vaccination sequence according to the WHO recommendations (e.g., BCG, DTP1-3, MCV). We depict ‘DTP after BCG’ as ‘BCP<DTP’
2. Select estimates from randomized trials.
3. Select estimates adjusted for age and other vaccines.
4. Estimates of primary interest
   - BCG
     A. BCG at birth vs. no BCG in preference to
     B. BCG vs. no BCG
   - DTP
     A. BCG<DTP (any number of doses) vs. BCG in preference to
     B. BCG<DTP (1 or 1-2 doses) vs. BCG in preference to
     C. BCG<DTP (2 or more doses) vs. BCG in preference to
     D. DTP (any number of doses) vs. no DTP in preference to
     E. DTP (1 or 1-2 doses) vs. no DTP in preference to
     F. DTP (2 or more doses) vs. no DTP
   - MCV
     A. BCG<DTP<MCV vs. BCG<DTP in preference to
     B. BCG<MCV vs. BCG in preference to
     C. DTP<MCV vs. DTP in preference to
     D. MCV vs. no MCV
5. Select comparison with least co-administration of other vaccines, particularly when vs. unvaccinated children
6. Select comparison involving children from the same area
7. Select estimate obtained using landmark (rather than retrospective) approach
8. Select estimate obtained from general population children rather than subgroups (e.g., hospitalized children)
9. Select comparison including the most comprehensive adjustment for potential confounders.
10. Select result for the shortest period of follow-up
11. Select result with the largest sample size
12. Select comparison with vaccination strategies according to the WHO recommendations (e.g., BCG at birth, MCV vaccine at 9 months)
13. Select estimate using the methodological approach claimed to be superior or more correct
14. Select result from more recent article

We omitted studies in which all children in one of the comparison groups had two of the vaccines administered simultaneously. The sources of data used in the forest plots are described in the first column of the tables in Appendix 3.

Appendix 3: Table of characteristics of included studies

Studies of BCG vaccine

Ref ID (citation)<br>Region	Follow-up	Vaccine comparisons (type, strain, reason)	Study design	Inclusion/Exclusion criteria	Vaccine ascertainment	Mortality ascertainment	Total number of children<br>Effect modifier
**Bangladesh 1986-2001**[1]<br>**Matlab (60 km southeast of Dhaka)**	≤60 months	BCG vs. no BCG<br>(Vaccine type/strain not reported, research purpose)	Observational cohort	Registration within the Maternal Child Health and Family Planning Programme area-Birth before January 1, 2000	Recorded at the point of vaccination in a record keeping book	Verbal autopsy	39,625<br>No modifier reported
**Benin 1983-1987**[2]<br>**Pahou and Avlkete (Atlantic coast, 30 km from Cotonou)**	≤ 36 months	BCG vs. no BCG<br>(Vaccine type/strain not reported, routine vaccination)	Case-control study	Cases: died between January 1986-October 1987, aged < 3 years; Controls: matched to cases for age, sex and village of residence	Review of health centre records	Routine home visits-interview with parents	Cases: 74; Controls: 230<br>No modifier reported
**Burkina Faso 1985-1993**[3]<br>**Pissila and Yako area villages**	≤ 24 months	BCG vs. no BCG, BCG vs. no BCG; BCG+DTP vs. no BCG, no DTP; BCG+DTP vs. BCG; DTP vs. no DTP<br>(Vaccine type/strain not reported, research purpose)	Observational cohort	Infants born in rural communities	Patient held vaccination cards	Visits every 6-12 months	9,412<br>Modifier: sex
**Canada 1933-1945**[4]<br>**Indian Reserve of Qu'Apelle**	≤ 60 months	BCG vs. no BCG<br>(Vaccine type/strain coded by number, subcutaneously administered, research purpose)	RCT	Indian infants born in the region of Qu'Appelle Indian Health Unit during the period of October 1933 to December I945<br>Excluded immigration, tuberculosis, faulty vaccination of controls	Recorded at the point of vaccination	Post-mortem examinations	609<br>No modifier reported
**Ghana 1998-2004**[5]<br>**Kassena and Nankana**	≤ 60 months	BCG vs. no BCG<br>(Vaccine type/strain not reported, research purpose)	Observational cohort	Born between 1 January 1998 and 31 December 2004 in Kassena and Nankana district in Northern Ghana	Demographic Surveillance System – Annual survey	Demographic Surveillance System – Routine household visits	18,368<br>No modifier reported
**Guinea Bissau 1984-1987**[6]<br>**Oio,Biombo, Cacheu and Gabu**	≤ 14 months	BCG vs.no BCG<br>(Vaccine type/strain not reported, routine vaccination-campaigns)	Observational cohort	Children who were 2–8 months old at a village visit<br>Children aged 9 months who would normally receive measles vaccine have been excluded	Patient held vaccination cards-BHP records	Household visits	1,657<br>Modifier: sex
**Guinea Bissau 1989-2001**[7-9]<br>**Bissau area**	≤ 12 months	BCG vs. no BCG; BCG at 1 week vs. no BCG; BCG at 6 weeks vs. no BCG; BCG at 1 week vs. BCG at 6 weeks (At 6 and 12 months)<br>(Vaccine type/strain not reported, routine vaccination)	Observational cohort	Low birth weight children born at the central hospital in Bissau between 1989-1999 with a recorded vaccination status and still living in the study area at the first home visit	Review of vaccination health records-Patient held vaccination cards-Household visits	Not reported	845<br>Modifier: sex
**Guinea Bissau 1990-1996**[10-13]<br>**Guinea Bissau (5 rural areas)**	≤ 60 months	BCG vs.no BCG<br>(Vaccine type/strain not reported, research purpose)	Observational cohort	Follow-up of 15,351 women and their children born alive during 1990 and 1996 and residing in rural areas of Guinea-Bissau (children had to be visited twice to be included in the study)<br>Children whose cards could not be inspected were excluded	Patient held vaccination cards	Household visits	10,298<br>No modifier reported
**Guinea Bissau 2002-2008**[14-18]	≤ 12 months	Early vs. delayed BCG in LBW; early BCG (after birth) vs. no	RCT with 2x2 factorial design	Low birth weight infants < 2,5 kg<br>Excluded malformations, wrong treatment, lost	Recorded at the point of vaccination	Verbal autopsy	2,343<br>Modifier: sex, VAS

Ref ID (citation) Region	Follow-up	Vaccine comparisons (type, strain, reason)	Study design	Inclusion/Exclusion criteria	Vaccine ascertainment	Mortality ascertainment	Total number of children Effect modifier
**Bandim area**		BCG vaccination in LBW; early BCG (after birth) vs. delayed administration of BCG in LBW (Copenhagen type, administered intradermally, research purpose)		to follow-up			
**India 1987-1989**[19] **Shirur in Pune, Maharashtra**	≤ 60 months	BCG only or BCG after DTP vs. no BCG; DTP as the most recent vaccine vs. no DTP; Only BCG vs. DTP after BCG or DTP only; BCG out-of-sequence vs. DTP recommended schedule; BCG vs. no BCG, DTP vs. no DTP; BCG before vs. simultaneously with DTP; BCG before vs. BCG after DTP (Vaccine type/strain not reported, routine vaccination)	Observational cohort (re-analysis)	Low birth weight children born between December 1987 and November 1989 in 45 contiguous villages in Shirur Administrative Block in Pune District	Recorded at the point of vaccination-household visits	Not reported	4,129 No modifier reported
**India 1998-2002**[20] **Tamil Nadu (two rural districts)**	≤ 6 months	BCG vs. no BCG; BCG+DTP vs. BCG; BCG+DTP vs. no BCG, no DTP; BCG, no DTP vs. no BCG, no DTP; BCG vs. no BCG (Vaccine type/strain not reported, routine vaccination)	Observational cohort	Live births between 12 August 1998 and 14 February 2002 in two rural blocks of Tamil Nadu	Patient held vaccination cards-Interview with the mother	Routine household visits	11,619 Modifier: sex, VAS
**India 2006-2011**[21] **Ballabgarh block (28 villages)**	≤ 36 months	BCG vs. no BCG; BCG+DTP vs. BCG; BCG+DTP+MCV vs. BCG+DTP; BCG+DTP vs. no BCG, no DTP; BCG+DTP+MCV vs. no BCG, no DTP, no MCV (Age groups: 0-5 weeks, 1.5-8 months, 9-15 months, 16-36 months) (Vaccine type/strain not reported, research purpose)	Observational cohort	Children born between 1 January 2006-31 December 2011 from 28 villages of the of Ballabgarh block in North India	Review of electronic database	Review of electronic database	12,412 Modifier: sex
**Malawi 1995-1997**[22] **Lungwena**	≤ 60 months	BCG vs. no BCG; BCG as the last vaccine received vs.no BCG (Vaccine type/strain not reported, routine vaccination)	Observational cohort	Children surviving the first week of age born between July 1995 and February 1997 in the antenatal clinic in Lungwena	Review of health centre records-Household visits-Vaccination cards	Verbal autopsy	751 Modifier: sex
**Papua New Guinea 1989-1994**[23] **Tari, Papua New Guinea Highlands**	≤ 24 months	BCG vs. no BCG; BCG vs. unvaccinated; BCG+DTP vs. BCG; DTP vs. no DTP; DTP vs. unvaccinated; BCG after vs. DTP before (Age groups: 29 days-5 months, 6-11 months, 12-23 months) (Vaccine type/strain not reported, routine vaccination)	Observational cohort	All children under demographic surveillance, born in Tari, between 1989 and 1994	Review of vaccination centre records	Verbal autopsy	6,665 Modifier: sex
**Senegal 1996-1999**[24,25] **Niakhar**	≤ 24 months	BCG+MCV vs. unvaccinated; BCG after DTP1 vs. unvaccinated;	Observational cohort	Children born in Niakhar, Senegal between September 1996 and December 1999	Recorded at the point of vaccination-Household	Not reported	4,133 Modifier: sex

Ref ID (citation) Region	Follow-up	Vaccine comparisons (type, strain, reason)	Study design	Inclusion/Exclusion criteria	Vaccine ascertainment	Mortality ascertainment	Total number of children Effect modifier
		DTP1 after BCG vs. unvaccinated; BCG+DTP vs. BCG; BCG+DTP1 vs. BCG; BCG+DTP1 vs. unvaccinated; BCG+DTP1-2 vs. unvaccinated; BCG+DTP1-3 vs. unvaccinated; BCG+DTP+MCV vs. unvaccinated (Vaccine type/strain not reported, routine vaccination)			visits-Vaccination card		
**USA c.1935**[26] **Arizona,Wyoming, Dakota, Alaska**	≤ 60 months	BCG vs. No BCG	Quasi-RCT	Native American Indians, aged 1-20 years, residents of Arizona, Wyoming, Dakota, Alaska who failed to react or gave doubtful reaction to tuberculin PPD skin test	Recorded at the point of vaccination	Not reported	3,008 (846 children ≤ 4 years of age) No modifier reported
**USA c.1941**[27] **Chicago**	≤ 60 months	BCG vs. No BCG ("Fresh", "freeze-dried" BCG vaccine, research purpose)	Quasi-RCT	Participants were newborns from tuberculous households placed in foster homes	Recorded at the point of vaccination	Autopsy	451 No modifier reported

In addition, three reports referred to studies that were excluded from analyses presented here.[8,28,29]

Studies of DTP vaccine

Ref ID (citation) Region	Follow-up	Vaccine comparisons (type, strain, reason)	Study design	Inclusion/Exclusion criteria	Vaccine ascertainment	Mortality ascertainment	Total number of children Effect modifier
**Bangladesh 1986-2001**[1,30] **Matlab (60 km southeast of Dhaka)**	≤9 months	Different sequence combinations of BCG and DTP vaccines: BCG+DTP1 vs. BCG (1.5-9 months); BCG simultaneously with DTP1 vs. DTP1 after BCG; BCG after DTP vs. DTP1 after BCG (1.5-9 months) (Vaccine type/strain not reported, routine vaccination)	Observational cohort	Children born between 1986 and 1999 within the Matlab HDSS	Routine household visits, recorded at the point of vaccination in a record-keeping book	Verbal autopsy as part of the routine HDSS	37,894 Modifier: sex
**Benin 1983-1987**[2] **Pahou and Avlkete (atlantic coast, 30 km from Cotonou)**	≤ 36 months	DTP1 vs. no DTP; DTP2 vs. no DTP; DTP3 vs. no DTP; DTP (4 doses) vs. no DTP (Vaccine type/strain not reported, routine vaccination)	Case-control study	Cases: died between January 1986-October 1987, aged < 3 years; Controls: matched to cases for age, sex and village of residence	Review of health centre records	Routine home visits-interview with parents	Cases: 74; Controls: 230 No modifier reported
**Burkina Faso 1985-1993**[3] **Pissila and Yako area villages**	≤ 24 months	DTP vs. no DTP; DTP1-2 vs. DTP1 (Vaccine type/strain not reported, research purpose)	Observational cohort	Infants born in rural communities	Patient held vaccination cards	Visits every 6-12 months	9,412 Modifier: sex

Ref ID (citation) Region	Follow-up	Vaccine comparisons (type, strain, reason)	Study design	Inclusion/Exclusion criteria	Vaccine ascertainment	Mortality ascertainment	Total number of children Effect modifier
**Ghana 1984-1991**[31 32] **Navrongo**	≤ 90 months	BCG+DTP1-2 vs. BCG (within 4 months of follow-up); BCG+DTP1-2 vs. BCG (within 24 months of follow-up); DTP (any) vs. assumed as unvaccinated (within 4 months of follow-up); DTP (3-4 doses) vs. DTP (0-2 doses) (Vaccine type/strain not reported, routine vaccination)	Observational cohort	Aged 6-90 months at study period, valid information regarding possession of a health card	Vaccination cards	Routine visits to homes and verbal autopsy	3,330 Modifier: sex
**Ghana 1998-2004**[5] **Kassena and Nankana**	≤ 60 months	DTP1 vs. no DTP; DTP2 vs. no DTP; DTP3 vs. no DTP (Vaccine type/strain not reported, research purpose)	Observational cohort	Born between 1 January 1998 and 31 December 2004 in Kassena and Nankana district in Northern Ghana	Demographic Surveillance System – Annual survey	Demographic Surveillance System – Routine household visits	18,368 No modifier reported
**Guinea Bissau 1984-1987**[6] **Oio,Biombo, Cacheu and Gabu**	≤ 14 months	DTP vs. no DTP; DTP1 vs. no DTP; DTP2-3 vs. no DTP (Vaccine type/strain not reported, routine vaccination-campaigns)	Observational cohort	Children who were 2–8 months old at a village visit	Patient held vaccination cards-BHP records	Household visits	1,657 Modifier: sex
**Guinea Bissau 1989-2001**[9,33-36] **Bandim I, Bandim II, Belem and Mindara (4 districts in Bissau)**	≤ 20 months	DTP vs. no DTP; DTP & Polio vs. DTP & Polio unvaccinated (Vaccine type/strain not reported, routine vaccination)	Observational cohort	Not reported (The study included 1491 children aged 1–17 months in four urban districts in Bissau)	Routine Household visits-Vaccination cards-Review of vaccination centre records	Verbal autopsy	2,800 Modifier: sex
**Guinea Bissau 1990-1996**[10-13,37] **Guinea Bissau (5 rural areas)**	≤ 60 months	BCG vs.no BCG; BCG+DTP1 vs. BCG; DTP1 vs. no DTP (Vaccine type/strain not reported, routine vaccination)	Observational cohort	Aged 0-6 months, during 1990-1996	Patient held vaccination cards	Household visits	10298 children analysed Modifier: sex
**Guinea Bissau 1996-2002**[37,38] **Paediatric ward at Bissau hospital**	≤ 20 months	DTP vs. no DTP (Vaccine type/strain not reported, routine vaccination)	Observational cohort	Hospitalized children 1-60 months old.	Vaccination cards, interview with moths or guardian	Observation in hospital	3,133 No modifier reported
**Guinea Bissau 2002-2008** [15,39] **Bandim area (6 suburban districts)**	≤ 6 months	DTP vs. no DTP; DTP vs. delayed DTP (all delayed BCG) ; BCG+DTP vs. BCG+delayed DTP (Vaccine type and strain not reported, routine vaccination)	RCT	Low birth weight children coming for their first vaccination	Vaccination cards	Verbal autopsy	2,343 Modifier: sex
**India 1987-1989**[19] **Shirur in Pune, Maharashtra**	≤ 60 months	BCG only or BCG after DTP vs. no BCG; DTP as the most recent vaccine vs. no DTP; Only BCG vs. DTP after BCG or DTP only; BCG out-of-sequence vs. DTP recommended schedule; MCV+DTP simultaneously vs. MCV only; DTP vs. no DTP; BCG before vs. simultaneously with DTP; BCG before vs. BCG	Observational cohort (re-analysis)	Low birth weight children born between December 1987 and November 1989 in 45 contiguous villages in Shirur Administrative Block in Pune District	Recorded at the point of vaccination-household visits	Not reported	4,129 No modifier reported

Ref ID (citation) Region	Follow-up	Vaccine comparisons (type, strain, reason)	Study design	Inclusion/Exclusion criteria	Vaccine ascertainment	Mortality ascertainment	Total number of children Effect modifier
		after DTP; MCV and DTP simultaneously vs. MCV before DTP (Vaccine type/strain not reported, routine vaccination)					
**India 1998-2002**[20] **Tamil Nadu (two rural districts)**	≤ 6 months	DTP vs. no DTP; DTP vs. no DTP (Vaccine type/strain not reported, routine vaccination)	Observational cohort	Live births between 12 August 1998 and 14 February 2002 in two rural blocks of Tamil Nadu	Patient held vaccination cards-Interview with the mother	Routine household visits	11,619 Modifier: Sex, VAS
**India 2006-2011**[21] **Ballabgarh block (28 villages)**	≤ 36 months	BCG+DTP vs. BCG; BCG+DTP+MCV vs. BCG+DTP; BCG+DTP vs. no BCG, no DTP; BCG+DTP+MCV vs. no BCG, no DTP, no MCV (Age groups: 0-5 weeks, 1.5-8 months, 9-15 months, 16-36 months) (MCV: monovalent type, research purpose)	Observational cohort	Children born between 1 January 2006-31 December 2011 from 28 villages of the of Ballabgarh block in North India	Review of electronic database	Review of electronic database	12,412 Modifier: sex
**Malawi1995-1997**[22] **Lungwena**	≤ 60 months	DTP1 vs. no DTP; DTP2 vs. no DTP; DTP (3 doses) vs. no DTP; BCG as the last vaccine received vs.no BCG; DTP1 as the last vaccine received vs. no DTP; DTP2 as the last vaccine received vs. no DTP; DTP3 as the last vaccine received vs. no DTP (Vaccine type/strain not reported, routine vaccination)	Observational cohort	Children surviving the first week of age born between July 1995 and February 1997 in the antenatal clinic in Lungwena	Review of health centre records-Household visits-Vaccination cards	Verbal autopsy	751 Modifier: sex
**Papua New Guinea1989-1994**[23] **Tari, Papua New Guinea Highlands**	≤ 24 months	BCG+DTP vs. BCG; DTP vs. no DTP; DTP vs. unvaccinated; DTP1-2 vs. unvaccinated; DTP vs. no DTP in children who received BCG; BCG after vs. DTP before (Age groups: 29 days-5 months, 6-11 months, 12-23 months) (Vaccine type/strain not reported, routine vaccination)	Observational cohort	All children under demographic surveillance, born in Tari, between 1989 and 1994	Review of vaccination centre records	Verbal autopsy	6,665 Modifier: sex
**Philippines 1988-1991**[40,41] **Metro Cebu**	≤ 30 months	DTP vs. no DTP (vaccine type/strain not reported, research purpose)	Observational cohort	Children under 30 months of age who received a BCG vaccination from July 1988 to January 1991	Vaccination cards-Review of vaccination centre records	Post mortem interviews	18,964 Modifier: sex
**Senegal 1996-1999**[24] **Niakhar**	≤ 24 months	DTP1 before BCG vs. unvaccinated; DTP1 after BCG vs. unvaccinated; BCG+DTP vs. BCG; BCG+DTP1 vs. BCG; BCG+DTP1 vs. unvaccinated; BCG+DTP1-2 vs. unvaccinated; BCG+DTP1-3 vs. unvaccinated;	Observational cohort	Children born in Niakhar, Senegal between September 1996 and December 1999	Recorded at the point of vaccination-Household visits-Vaccination card	Not reported	4,133 Modifier: sex

Ref ID (citation) Region	Follow-up	Vaccine comparisons (type, strain, reason)	Study design	Inclusion/Exclusion criteria	Vaccine ascertainment	Mortality ascertainment	Total number of children Effect modifier
		BCG+DTP+MCV vs. unvaccinated; DTP1+MCV vs. unvaccinated; DTP1-2+MCV vs. unvaccinated; DTP1-3+MCV vs. unvaccinated (Vaccine type/strain not reported, routine vaccination)					

In addition, six reports referred to studies that were excluded from analyses presented here.[8,25,28,42-44]

Studies of measles-containing vaccine (MCV)

Ref ID (citation) Region	Follow-up	Vaccine comparisons (type, strain, reason)	Study design	Inclusion/Exclusion criteria	Vaccine ascertainment	Mortality ascertainment	Total number of children Effect modifier
**Bangladesh 1977-1985**[45-48] **Matlab (45 km southeast of Dhaka; Blocks A,B,C,D)**	≤ 59 months	MCV vs. no MCV (Schwarz F88, Research purpose)	Observational cohort	Measles vaccinated and unvaccinated children aged 9-60 months and observed from March 1982 to October 1985, No record of MCV vaccination of the non vaccinees during the study period, vaccinees and corresponding non-vaccinees matched for month and year of birth, survival of each non-vaccinee at least up to the date of vaccination of their matched vaccinee	Routine visits to homes, Demographic surveillance system	Routine visits to homes, Demographic surveillance system	18,266 No modifier reported
**Bangladesh 1986-2001**[1] **Matlab (60 km southeast of Dhaka)**	≤60 months	MCV vs. no MCV (Vaccine type/strain not reported, research purpose)	Observational cohort	Registration within the Maternal Child Health and Family Planning Programme area-Birth before January 1, 2000	Recorded at the point of vaccination in a record-keeping book	Verbal autopsy	39,625 No modifier reported
**Benin 1983-1987**[2] **Pahou and Avlkete (atlantic coast, 30 km from Cotonou)**	≤ 36 months	BCG vs. no BCG; DTP1 vs. no DTP; DTP2 vs. no DTP; DTP3 vs. no DTP; DTP (4 doses) vs. no DTP; MCV vs. no MCV; MCV after 12 months vs. no MCV (Vaccine type/strain not reported, routine vaccination)	Case-control study	Cases: died between January 1986-October 1987, aged < 3 years; Controls: matched to cases for age, sex and village of residence	Review of health centre records	Routine home visits-interview with parents	Cases: 74; Controls: 230 No modifier reported
**Burundi 1984-1988**[49] **Muyinga sector**	≤ 60 months	MCV vs. no MCV (Monovalent, vaccine strain not reported, research purpose)	Observational cohort	Children < 5 years of age in 5 hills in Muyinga born since January 1984 and alive at July 1, 1988	Patient held vaccination cards – Household visits	Door-to-door census	1,899 No modifier reported
**Democratic Republic of Congo**[50] **Kasongo**	≤ 60 months	MCV vs. no MCV (Monovalent, Live attenuated measles vaccine ('Atenuvax', Merck, Sharp and Dohme), routine vaccination)	Observational cohort	Children <5 years of age in Kasongo	Recorded at the point of vaccination	Routine household visits	7, 092 No modifier reported

Ref ID (citation)<br>Region	Follow-up	Vaccine comparisons (type, strain, reason)	Study design	Inclusion/Exclusion criteria	Vaccine ascertainment	Mortality ascertainment	Total number of children<br>Effect modifier
**Ghana 1984-1991**[32]<br>**Navrongo**	≤ 90 months	BCG+DTP+MCV vs. BCG+DTP (mortality between 10-39 months) (Vaccine type/strain not reported, administered in campaign)	Observational cohort	Vaccination card seen, no card	Vaccination cards	Routine visits to homes and verbal autopsy	3,330<br>Modifier: sex
**Ghana 1994-1999**[28]<br>**Kassena and Nankana (later in Navrongo)**	≤ 60 months	Adjusted comparisons: Partial Partial BCG/DTP + MCV vs. no BCG, no DTP; Full BCG/ DTP/ MCV vs. no BCG, no DTP<br>Unadjusted comparison: MCV vs. no MCV with partial or full BCG/DTP coverage; BCG+ DTP3 +MCV vs. BCG+DTP3<br>(Age groups: 9-11 months, 12-23 months, 24-59 months)<br>(Vaccine type/strain not reported, routine vaccination)	Observational cohort	Vaccinated and unvaccinated children under 5 years of age (data from Navrongo	Review of health centre records – Patient held vaccination cards	Routine visits to homes	24,053<br>No modifier reported
**Ghana 1998-2004**[5]<br>**Kassena and Nankana**	≤ 60 months	MCV vs. no MCV<br>(Vaccine type/strain not reported, research purpose)	Observational cohort	Born between 1 January 1998 and 31 December 2004 in Kassena and Nankana district in Northern Ghana	Demographic Surveillance System – Annual survey	Demographic Surveillance System – Routine household visits	18,368<br>No modifier reported
**Guinea Bissau 1978-1983**[51-53]<br>**Bandim I, Quinhamel**	≤ 35 months	MCV vs. no MCV<br>(Vaccine type/strain not reported, research purpose)	Observational cohort	Not reported	Not reported	Not reported	489 (Quinhamel)<br>682 (Bandim)<br>No modifier reported
**Guinea Bissau 1984-1987** [54,55]<br>**Bandim I, Bandim II**	≤ 34 months	MCV vs. no MCV<br>(Monovalent, Schwarz measles vaccine, research purpose)	Observational cohort	Born between 1 August 1984 and 31 September 1985, registered in Bandim I and II before 4 months of age	Recorded at the point of vaccination (not clear this was the case)-Household visits	Routine household visits	2,722<br>No modifier reported
**Guinea Bissau 1989-2001**[8,33-35,56-59]<br>**Belem and Mindara (districts of Bissau)**	≤ 18 months	Early MCV (at 6 months) vs. IPV (at 6 months)<br>(Schwarz measles vaccine, research purpose)	RCT (re-analyses)	Not reported	Not reported	Not reported	300<br>Modifier: VAS
**Guinea Bissau 1990-1996**[13]<br>**Guinea Bissau (5 rural areas)**	≤ 60 months	Unadjusted comparison: MCV vs. no MCV (Age groups: 0-1 months, 2-3 months, 4-6 months, 7-8 months, 9-11 months, 12-13 months)<br>(Vaccine type/strain not reported, research purpose)	Observational cohort	Alive at the first visit and residing in the area-children had to be visited twice to be included in the study	Patient held vaccination cards	Household visits	10,298<br>No modifier reported
**Guinea Bissau 1999-2006**[60]<br>**BHP study area (rural)**	12-35 months	MCV vs. no MCV<br>(Vaccine type/strain not reported)	Observational cohort	Children aged 12-35 months who had their vaccination card inspected between 1 January 1999 and 15 May 2006, the date of the national MCV campaign, were included in the survival analysis	Routine household visits-Vaccination cards	Routine Household visits	18,119<br>Modifier: Sex
**Guinea Bissau 2002-2008**[39,59,61,62]<br>**Bandim (6 suburban districts)**	≤ 36 months	Early MCV vs. no MCV; Two doses MCV (4.5 & 9 months) vs. one dose MCV (9 months)<br>(Monovalent type, Edmonston	RCT	Children aged 4.5 months of age who had received 3 doses of DTP vaccine at least 4 weeks before study period	Recorded at the point of vaccination	Routine household visits - Visits to the health centres	6,648<br>Modifier: sex

Ref ID (citation) Region	Follow-up	Vaccine comparisons (type, strain, reason)	Study design	Inclusion/Exclusion criteria	Vaccine ascertainment	Mortality ascertainment	Total number of children Effect modifier
		and Schwarz strain, research purpose)					
**Guinea Bissau 2004-2009**[42,43] **BHP study area (urban and rural)**	6-35 months	MCV vs. no MCV (Vaccine type/strain not reported)	Observational cohort	Two studies: 1. Children born 2004-2008 living in the urban study area aged 6-35 months at the start of vitamin A supplementation campaigns in 2007 and 2008[42] 2. Children born 2006-2009 living in urban and rural areas aged 6-23 months who were due a vaccination were recruited to a randomized trial of vitamin A supplementation[43]	1. Recorded at the point of vaccination and through 3-monthly home visits, or administered in the context of a vaccination 2. Administered at the start of the vitamin A supplementation trial randomized trial	1. Verbal autopsy 2. Recorded in the context of a randomized trial	Modifier: VAS
**Haiti 1981-1982**[63] **Haiti (Cite Soleil, periurban slum)**	≤ 39 months of age	MCV (monovalent, research purpose) compared to unvaccinated children	Observational cohort	Births in Cite Soleil between October 1981 and April 1982; Children that survived to 9 months of age and received MCV vaccine	Review of health Centre records-Measles trial database	Questionnaire fulfilled by mother including date and age of the child at death	1,381<br><br>No modifier reported
**India 1985-1991**[64] **Kaniyambadi block (60 villages)**	≤ 60 months	MCV vs. no MCV (Monovalent type, Edmonston-Zagreb and Schwarz type, administered in campaign)	Observational cohort	Children born to residents of the area between January 1 1986 and December 31 1991	Household visits	Household visits-Verbal autopsy-Hospital records	16,665<br>No modifier reported
**India 1987-1989**[19] **Shirur in Pune, Maharashtra**	≤ 60 months	MCV+DTP simultaneously vs. MCV only; MCV before DTP vs. MCV only; MCV and DTP simultaneously vs. MCV before DTP; MCV vs. no MCV (Vaccine type/strain not reported, routine vaccination)	Observational cohort (re-analysis)	Low birth weight children born between December 1987 and November 1989 in 45 contiguous villages in Shirur Administrative Block in Pune District	Recorded at the point of vaccination-household visits	Not reported	4,129<br>No modifier reported
**India 1991-1998**[65] **Ballabgarh block (28 villages)**	12-59 months	MCV vs. no MCV in cases and controls (Vaccine type/strain not reported, routine vaccination)	Case-control study	Children aged 12-59 months born between 1 January 1991 and 31 December 1998, registered in the electronic database of the project area	Socio-demographic database	Multipurpose health workers registered deaths during routine domiciliary visits	330 cases and 320 controls<br>No modifier reported
**India 2006-2011**[21] **Ballabgarh block (28 villages)**	≤ 36 months	BCG+DTP+MCV vs. BCG+DTP; BCG+DTP+MCV vs. no BCG, no DTP, no MCV (Age groups: 0-5 weeks, 1.5-8 months, 9-15 months, 16-36 months) (MCV: monovalent type, research purpose)	Observational cohort	Children born between 1 January 2006-31 December 2011 from 28 villages of the of Ballabgarh block in North India	Review of electronic database	Review of electronic database	12,412<br>Modifier: sex
**Malawi 1995-1997**[22] **Lungwena**	≤ 60 months	MCV as the last vaccine received vs. no MCV (Vaccine type/strain not reported, routine vaccination)	Observational cohort	Children surviving the first week of age born between July 1995 and February 1997 in the antenatal clinic in Lungwena	Review of health centre records-Household visits-Vaccination cards	Verbal autopsy	751<br>Modifier: sex
**Nigeria 1956-1961**[66] **Imesi and Ilesha**	≤ 59 months	MCV vs. no MCV; MCV vs. pertussis/tetanus vaccines (Enders B strain, research purpose)	Observational cohort	Not clearly stated	Recorded at the point of vaccination	Not reported	2,053<br>No modifier reported
**Papua New Guinea 1989-1994**[23]	≤ 24 months	MCV vs. no MCV; MCV vs. unvaccinated	Observational cohort	All children under demographic surveillance, born in Tari, between 1989 and 1994	Review of vaccination centre records	Verbal autopsy	6,665<br>Modifier: sex

Ref ID (citation) Region	Follow-up	Vaccine comparisons (type, strain, reason)	Study design	Inclusion/Exclusion criteria	Vaccine ascertainment	Mortality ascertainment	Total number of children Effect modifier
**Tari, Papua New Guinea Highlands**		(Vaccine type/strain not reported, routine vaccination)					
**Senegal 1985-1987**[67] **Niakhar**	≤ 5 years	MCV vs. unvaccinated (Age groups: 9-23 months, 24-60 months) (Schwarz standard measles vaccine, research purpose)	Observational cohort	Children born in the Niakhar region in Senegal, from resident mothers, from February 1985 to January 1987	Annual Demographic monitoring system-Household visits	Post-mortem parental interviews	2,417 Modifier: sex
**Senegal 1987-1989**[67] **Niakhar**	≤ 5 years	MCV vs. unvaccinated (Age groups: 9-23 months, 24-60 months) (Schwarz standard measles vaccine, research purpose)	Observational cohort	Children born in the Niakhar region in Senegal, from resident mothers, between February 1, 1985-January 31,1991	Recorded at the point of vaccination-weekly Demographic monitoring system-Household visits	Post-mortem parental interviews	2,417<br><br>Modifier: sex
**Senegal 1989-1996**[25,67,68] **Niakhar**	≤ 24 months	MCV vs. no MCV; MCV vs. no MCV (± BCG or DTP) (MCV: Measles-Rubella type, routine vaccination)	Observational cohort (1989-1996 and 1996-1999 cohorts)	The first cohort included 8277 children born in the Niakhar area between 1 September 1989 and 31 August 1996. The second cohort included 4114 children born in the same area between 1 September 1996 and 31 December 1999	Recorded at the point of vaccination-Patient held vaccination cards	Household visits	12,391 No modifier reported
**Senegal 1996-1999**[24,25] **Niakhar**	≤ 24 months	BCG+MCV vs. unvaccinated; BCG+DTP+MCV vs. unvaccinated; DTP1+MCV vs. unvaccinated; DTP1-2+MCV vs. unvaccinated; DTP1-3+MCV vs. unvaccinated (Vaccine type/strain not reported, routine vaccination)	Observational cohort	Children born in Niakhar, Senegal between September 1996 and December 1999	Recorded at the point of vaccination-Household visits-Vaccination card	Not reported	4,133 Modifier: sex

References of included articles

1. Breiman RF, Streatfield PK, Phelan M, Shifa N, Rashid M, Yunus M. Effect of infant immunisation on childhood mortality in rural Bangladesh: analysis of health and demographic surveillance data. *Lancet* 2004; **364**: 2204-11.
2. Velema JP, Alihonou EM, Gandaho T, Hounye FH. Childhood mortality among users and non-users of primary health care in a rural west African community. *Int J Epidemiol* 1991; **20**: 474-9.
3. Vaugelade J, Pinchinat S, Guiella G, Elguero E, Simondon F. Non-specific effects of vaccination on child survival: Prospective cohort study in Burkina Faso. *British Medical Journal* 2004; **329**: 1309-11.
4. Ferguson RG, Simes AB. BCG vaccination of Indian infants in Saskatchewan. *Tubercle* 1949; **30**: 5-11.
5. Bawah AA, Phillips JF, Adjuik M, Vaughan-Smith M, Macleod B, Binka FN. The impact of immunization on the association between poverty and child survival: evidence from Kassena-Nankana District of northern Ghana. *Scandinavian journal of public health* 2010; **38**: 95-103.
6. Aaby P, Jensen H, Gomes J, Fernandes M, Lisse IM. The introduction of diphtheria-tetanus-pertussis vaccine and child mortality in rural Guinea-Bissau: An observational study. *Int J Epidemiol* 2004; **33**: 374-80.

7. Roth A, Jensen H, Garly ML, et al. Low birth weight infants and Calmette-Guerin bacillus vaccination at birth: Community study from Guinea-Bissau. *Pediatric Infectious Disease Journal* 2004; **23**: 544-50.
8. Garly ML, Martins CL, Bale C, et al. BCG scar and positive tuberculin reaction associated with reduced child mortality in West Africa: A non-specific beneficial effect of BCG? *Vaccine* 2003; **21**: 2782-90.
9. Garly ML, Trautner SL, Marx C, et al. Thymus Size at 6 Months of Age and Subsequent Child Mortality. *Journal of Pediatrics* 2008; **153**: 683-8.e3.
10. Aaby P, Jensen H. Routine vaccinations and child survival: effect of gender. *BMJ* 2002: http://www.bmj.com/rapid-response/2011/10/29/routine-vaccinations-and-child-survival-effect-gender.
11. Jensen H, Benn C, Nielsen J, Lisse IM, Rodrigues A, Aaby P. Diphtheria-tetanus-pertussis vaccination in low-income countries: improved child survival or survival bias? . *BMJ* 2005.
12. Jensen H, Benn CS, Lisse IM, Rodrigues A, Andersen PK, Aaby P. Survival bias in observational studies of the impact of routine immunizations on childhood survival. *Tropical Medicine and International Health* 2007; **12**: 5-14.
13. Kristensen I, Aaby P, Jensen H. Routine vaccinations and child survival: Follow up study in Guinea-Bissau, West Africa. *British Medical Journal* 2000; **321**: 1435-9.
14. Biering-Sorensen S, Aaby P, Napirna BM, et al. Small randomized trial among low-birth-weight children receiving bacillus Calmette-Gueerin vaccination at first health center contact. *Pediatric Infectious Disease Journal* 2012; **31**: 306-8.
15. Aaby P, Ravn H, Roth A, et al. Early diphtheria-tetanus-pertussis vaccination associated with higher female mortality and no difference in male mortality in a cohort of low birthweight children: An observational study within a randomised trial. *Archives of Disease in Childhood* 2012; **97**: 685-91.
16. Aaby P, Roth A, Ravn H, et al. Randomized trial of BCG vaccination at birth to low-birth-weight children: Beneficial nonspecific effects in the neonatal period? *Journal of Infectious Diseases* 2011; **204**: 245-52.
17. Benn CS, Fisker AB, Napirna BM, et al. Vitamin A supplementation and BCG vaccination at birth in low birthweight neonates: two by two factorial randomised controlled trial. *BMJ (Clinical research ed)* 2010; **340**: c1101.
18. Roth AE, Benn CS, Ravn H, et al. Effect of revaccination with BCG in early childhood on mortality: Randomised trial in Guinea-Bissau. *BMJ (Online)* 2010; **340**: 749.
19. Hirve S, Bavdekar A, Juvekar S, Benn CS, Nielsen J, Aaby P. Non-specific and sex-differential effects of vaccinations on child survival in rural western India. *Vaccine* 2012; **30**: 7300-8.
20. Moulton LH, Rahmathullah L, Halsey NA, Thulasiraj RD, Katz J, Tielsch JM. Evaluation of non-specific effects of infant immunizations on early infant mortality in a southern Indian population. *Tropical Medicine and International Health* 2005; **10**: 947-55.
21. Krishnan A, Srivastava R, Dwivedi P, Ng N, Byass P, Pandav CS. Non-specific sex-differential effect of DTP vaccination may partially explain the excess girl child mortality in Ballabgarh, India. *Tropical Medicine and International Health* 2013; **18**: 1329-37.
22. Aaby P, Vessari H, Nielsen J, et al. Sex differential effects of routine immunizations and childhood survival in rural Malawi. *Pediatric Infectious Disease Journal* 2006; **25**: 721-7.
23. Lehmann D, Vail J, Firth MJ, de Klerk NH, Alpers MP. Benefits of routine immunizations on childhood survival in Tari, Southern Highlands Province, Papua New Guinea. *Int J Epidemiol* 2005; **34**: 138-48.
24. Aaby P, Nielsen J, Benn CS, Trape J-F. Sex-differential and non-specific effects of routine vaccinations in a rural area with low vaccination coverage: an observational study from Senegal. *Transactions of the Royal Society of Tropical Medicine and Hygiene* 2015; **109**: 77-84.

25. Elguero E, Simondon KB, Vaugelade J, Marra A, Simondon F. Non-specific effects of vaccination on child survival? A prospective study in Senegal. *Tropical Medicine and International Health* 2005; **10**: 956-60.
26. Aronson JD. Protective vaccination against tuberculosis with special reference to BCG vaccination. *Am Rev Tuberc* 1948; **58**: 255-81.
27. Rosenthal SR, Loewinsohn E, Graham ML, Liveright D, Thorne MG, Johnson V. BCG vaccination in tuberculous households. *Am Rev Respir Dis* 1961; **84**: 690-704.
28. Nyarko PP, B.; Debpuur, C. Immunization status and child survival in rural Ghana. New York: Population Council, 2001.
29. Roth A, Sodemann M, Jensen H, et al. Tuberculin reaction, BCG scar, and lower female mortality. *Epidemiology* 2006; **17**: 562-8.
30. Aaby P, Ravn H, et al. Combined BCG and DTP vaccinations may reduce infant mortality more than the WHO-schedule of BCG first and then DTP: a re-analysis of demographic surveillance data from rural Bangladesh. *Unpublished*.
31. Benn CS, Aaby P, Nielsen J, Binka FN, Ross DA. Does vitamin A supplementation interact with routine vaccinations? An analysis of the Ghana Vitamin A Supplementation Trial. *American Journal of Clinical Nutrition* 2009; **90**: 629-39.
32. Welaga P, Nielsen J, Adjuik M, et al. Non-specific effects of diphtheria-tetanus-pertussis and measles vaccinations? An analysis of surveillance data from Navrongo, Ghana. *Tropical Medicine and International Health* 2012.
33. Aaby P, Jensen H, Garly ML, Bale C, Martins C, Lisse I. Routine vaccinations and child survival in a war situation with high mortality: Effect of gender. *Vaccine* 2002; **21**: 15-20.
34. Aaby P, Hedegaard K, Sodemann M, et al. Childhood mortality after oral polio immunisation campaign in Guinea-Bissau. *Vaccine* 2005; **23**: 1746-51.
35. Aaby P, Garly ML, Nielsen J, et al. Increased female-male mortality ratio associated with inactivated polio and diphtheria-tetanus-pertussis vaccines: Observations from vaccination trials in Guinea-Bissau. *Pediatric Infectious Disease Journal* 2007; **26**: 247-52.
36. Benn CS, Aaby P. Diphtheria-tetanus-pertussis vaccination administered after measles vaccine: Increased female mortality? *Pediatric Infectious Disease Journal* 2012; **31**: 1095-7.
37. Aaby P, Biai S, Veirum JE, et al. DTP with or after measles vaccination is associated with increased in-hospital mortality in Guinea-Bissau. *Vaccine* 2007; **25**: 1265-9.
38. Aaby P, Rodrigues A, Biai S, et al. Oral polio vaccination and low case fatality at the paediatric ward in Bissau, Guinea-Bissau. *Vaccine* 2004; **22**: 3014-7.
39. Benn CS, Martins C, Rodrigues A, et al. The effect of vitamin A supplementation administered with missing vaccines during national immunization days in Guinea-Bissau. *Int J Epidemiol* 2009; **38**: 304-11.
40. Chan GJ, Moulton LH, Becker S, Munoz A, BlackR.E RE. Non-specific effects of diphtheria - Tetanus - Pertussis vaccination on child mortality in Cebu, The Philippines. *Int J Epidemiol* 2007; **36**: 1022-9.
41. Chan GJ, Moulton LH, Becker S, Munoz A, Black RE. Author's response. *Int J Epidemiol* 2009; **38**: 323-4.
42. Fisker AB, Aaby P, Bale C, et al. Does the effect of vitamin A supplements depend on vaccination status? An observational study from Guinea-Bissau. *BMJ Open* 2012; **2**.
43. Fisker AB, Bale C, Rodrigues A, et al. High-dose vitamin A with vaccination after 6 months of age: a randomized trial. *Pediatrics* 2014; **134**: e739-e48.
44. Fisker AB, Ravn H, Rodrigues A, et al. Co-administration of live measles and yellow fever vaccines and inactivated pentavalent vaccines is associated with increased mortality compared with measles and yellow fever vaccines only. An observational study from Guinea-Bissau. *Vaccine* 2014; **32**: 598-605.
45. Aaby P, Bhuiya A, Nahar L, Knudsen K, de Francisco A, Strong M. The survival benefit of measles immunization may not be explained entirely by the prevention of measles disease: A community study from rural Bangladesh. *Int J Epidemiol* 2003; **32**: 106-15.

46. Bishai D, Koenig M, Ali Khan M. Measles vaccination improves the equity of health outcomes: Evidence from Bangladesh. *Health Economics* 2003; **12**: 415-9.
47. Clemens JD, Stanton BF, Chakraborty J, et al. Measles vaccination and childhood mortality in rural Bangladesh. *American Journal of Epidemiology* 1988; **128**: 1330-9.
48. Koenig MA, Khan MA, Wojtyniak B, et al. Impact of measles vaccination on childhood mortality in rural Bangladesh. *Bulletin of the World Health Organization* 1990; **68**: 441-7.
49. Chen RT, Weierbach R, Bisoffi Z, et al. A 'post-honeymoon period' measles outbreak in Muyinga sector, Burundi. *Int J Epidemiol* 1994; **23**: 185-93.
50. Van Balen H, Mercenier P, Daveloose P. Influence of measles vaccination on survival pattern of 7-35-month-old children in Kasongo, Zaire. *Lancet* 1981; **1**: 764-7.
51. Aaby P, Bukh J, Lisse IM, Smits AJ. Measles vaccination and reduction in child mortality: a community study from Guinea-Bissau. *Journal of Infection* 1984; **8**: 13-21.
52. Aaby P, Bukh J, Lisse IM, et al. Determinants of measles mortality in a rural area of Guinea-Bissau: crowding, age, and malnutrition. *J Trop Pediatr* 1984; **30**: 164-8.
53. Aaby P, Andersen M, Sodemann M, Jakobsen M, Gomes J, Fernandes M. Reduced childhood mortality after standard measles vaccination at 4-8 months compared with 9-11 months of age. *British Medical Journal* 1993; **307**: 1308-11.
54. Aaby P, Lisse IM, Whittle H, et al. Long-term survival in trial of medium-titre Edmonston-Zagreb measles vaccine in Guinea-Bissau: Five-year follow-up. *Epidemiology and Infection* 1994; **112**: 413-20.
55. Aaby P, Knudsen K, Jensen TG, et al. Measles incidence, vaccine efficacy, and mortality in two urban African areas with high vaccination coverage. *Journal of Infectious Diseases* 1990; **162**: 1043-8.
56. Benn CS, Bale C, Sommerfelt H, Friis H, Aaby P. Hypothesis: Vitamin A supplementation and childhood mortality: Amplification of the non-specific effects of vaccines? *Int J Epidemiol* 2003; **32**: 822-8.
57. Veirum JE, Sodemann M, Biai S, et al. Routine vaccinations associated with divergent effects on female and male mortality at the paediatric ward in Bissau, Guinea-Bissau. *Vaccine* 2005; **23**: 1197-204.
58. Aaby P, Garly ML, Bale C, et al. Survival of previously measles-vaccinated and measles-unvaccinated children in an emergency situation: An unplanned study. *Pediatric Infectious Disease Journal* 2003; **22**: 798-805.
59. Aaby P, Martins CL, Garly ML, et al. Measles vaccination in the presence or absence of maternal measles antibody: impact on child survival. *Clinical Infectious Diseases* 2014; **59**: 484-92.
60. Fisker AB, Hornshøj L, Rodrigues A, et al. Effects of the introduction of new vaccines in Guinea-Bissau on vaccine coverage, vaccine timeliness, and child survival: an observational study. *The Lancet Global Health* 2014; **2**: e478-e87.
61. Aaby P, Martins CL, Garly ML, et al. Non-specific effects of standard measles vaccine at 4.5 and 9 months of age on childhood mortality: Randomised controlled trial. *BMJ (Online)* 2010; **341**: 1262.
62. Benn CS, Martins C, Fisker AB, et al. Interaction between neonatal vitamin A supplementation and timing of measles vaccination: A retrospective analysis of randomised trials. (unpublished manuscript).
63. Holt EA, Boulos R, Halsey NA, Boulos LM, Boulos C. Childhood survival in Haiti: Protective effect of measles vaccination. *Pediatrics* 1990; **85**: 188-94.

64. George K, Joseph A, Muliyil J, Abraham S, Bhattacharji S, John KR. Measles vaccination before nine months. *Tropical Medicine and International Health* 1998; **3**: 751-6.
65. Kabir Z, Long J, Reddaiah VP, Kevany J, Kapoor SK. Non-specific effect of measles vaccination on overall child mortality in an area of rural India with high vaccination coverage: A population-based case-control study. *Bulletin of the World Health Organization* 2003; **81**: 244-50.
66. Hartfield J, Morley D. Efficacy of measles vaccine. *J Hyg* 1963; **61**: 143-7.
67. Aaby P, Samb B, Simondon F, et al. Divergent mortality for male and female recipients of low-titer and high- titer measles vaccines in rural Senegal. *American Journal of Epidemiology* 1993; **138**: 746-55.
68. Aaby P, Samb B, Simondon F, et al. A comparison of vaccine efficacy and mortality during routine use of high-titre Edmonston-Zagreb and Schwarz standard measles vaccines in rural Senegal. *Transactions of the Royal Society of Tropical Medicine and Hygiene* 1996; **90**: 326-30.

Appendix 4: Excluded studies

Of the 877 full-text articles identified as potentially relevant for the review, 770 were excluded (See Figure 1). The reasons for exclusion for these studies were as follows.

Level (N excluded)	Question	Answer	Number excluded per answer
**Full-text screening (N = 639)**	Did the study reported on BCG, DTP or Measles-containing vaccines	No	78
	Study design	Cross-sectional	51
		Not primary research	342
		Uncontrolled studies	21
		Other not relevant designs	27
	Were participants children ≤ 5 years of age?	No	9
		Not applicable	339
	Did the study report all-cause or non-target mortality data?	No	65
		No mortality data reported	542
**Eligibility (N = 126)**	Appropriate study design?	No	90
	Mortality reported as an outcome?	No	51
	BCG/DTP/MV examined?	No	37
	Outcome data in children ≤ 5 years of age?	No	14

Appendix 5: Assessments of risk of bias

Risk-of-bias assessments for randomized and quasi-randomized trials of BCG vaccine

Canada 1933-1945[4]

Result selected	Sample	Allocation or randomization	Blinding and co-interventions	Ascertainment of mortality	Data & analysis
**Risk ratio (unadjusted) = 0.94 (0.67, 1.32) Computed frm counts in Table IV (first row, less than 1 year)**	Quasi-randomized trial of BCG vs. no BCG. All Indian infants born in the Qu'Appelle Indian Health Unit between October 1933 and December 1945. 309 vaccinated and 310 unvaccinated children. 306 and 303 respectively were included in the analyses.<br>Selected for inclusion in review: All.	Allocation: "Families of comparable status in respect of housing, sanitation and certain other economic and social factors likely to affect the health of children were paired, and one member of each such pair was allotted at random to one of two groups, designated 'Group A' and 'Group B'. All children born into the families of Group A were vaccinated in one year, while all children born into families of Group B in the same year were taken as controls. In the following year this situation would be reversed and so on throughout the duration of the study." Allocation appears to be random in principle, but unlikely to be adequately concealed. No information on similarity of groups.	Blinding of participants: None.<br>Blinding of vaccine administrators: None.<br>Co-interventions and departures from allocated intervention: No information.	Assessed at 12-month visit.<br>Blinding of outcome assessors: Probably not, but objective outcome.	Missing data: Outcome data reasonably complete.<br>Analysis: Unadjusted comparison of numbers of deaths.

Unclear risk of bias due to confounding (allocation may be unconcealed and no information about similarity of groups)
Moderate risk of performance bias due to deviations from intended interventions (participants knew vaccination status)
Low risk of detection bias in measurement of outcomes
Low risk of attrition bias due to missing outcome data
Unclear risk of bias in selection of the reported result
Overall: Moderate risk of bias (participants knew vaccination status)

Guinea-Bissau 2002-2008 (early phase of trial)[14]

Result selected	Sample	Allocation or randomization	Blinding and co-interventions	Ascertainment of mortality	Data & analysis
**MRR (unadjusted) = 0.28 (0.06, 1.37) Table 1 (4 weeks)**	Randomized trial of early BCG vs. delayed BCG (only time before delayed BCG considered in this review). 105 low birth weight children from 2 health centres randomized Nov 2002 to Nov 2004. Selected for inclusion in review: All.	Allocation: Randomization methods described elsewhere.[16]	Blinding of participants: None.[16] Blinding of vaccine administrators: None.[16]	No details. Blinding of outcome assessors: Probably not, but objective outcome.	Missing data: Outcome data reasonably complete. Analysis: Cox proportional hazards model.

Low risk of bias due to confounding
Low risk of performance bias due to deviations from intended interventions
Low risk of detection bias in measurement of outcomes
Low risk of attrition bias due to missing outcome data
Unclear risk of bias in selection of the reported result
Overall: Low risk of bias

Guinea-Bissau 2002-2008 (main phase of trial)[16]

Result selected	Sample	Allocation or randomization	Blinding and co-interventions	Ascertainment of mortality	Data & analysis
**HR (adjusted) = 0.55 (0.34, 0.89) Table 3 total at 4 weeks**	Randomized trial of early BCG vs. delayed BCG (only time before delayed BCG considered in this review). 2343 low birth weight children from 3 health centres. 607 randomized November 2004 to May 2005, and 1736 randomized May 2005 to January 2008. In the second of these periods, trial extended to factorial design with addition of vitamin vs. placebo comparison. <u>Selected for inclusion in review</u>: All.	<u>Allocation</u>: "Block randomization procedures have been described in detail elsewhere. Twins were allocated the same treatment to prevent potential confusion regarding who had been vaccinated." A cited reference refers to the factorial period of the trial only:[17] "Once consent was provided, the mother drew an envelope from a bag. Each bag was prepared by the study supervisor and contained 48 envelopes; each envelope contained a lot name. Within each bag were 12 envelopes with lots marked "BCG 6," 12 marked "BCG 7," 12 marked "no BCG 6," and 12 marked "no BCG 7." The numbers "6" and "7" indicated from which of two numbered bottles, "6" or "7," the child should receive treatment (that is, either 25000 IU vitamin A or placebo)." "The envelopes were closed and non-transparent, making it impossible to identify the allocation before the envelopes were opened." "There were few differences in anthropometric measurements, gestational age, or background factors between children who received BCG and controls. The BCG group had more twins and tended to have more mothers who had died before enrollment".	<u>Blinding of participants</u>: "No placebo for BCG was given."; "a control vaccine may have a nonspecific impact on mortality. Furthermore, if we had used a placebo, control mothers might have believed that the child had received BCG and might therefore not have sought BCG vaccination. We therefore preferred not to use a placebo". <u>Blinding of vaccine administrators</u>: None. <u>Co-interventions and departures from allocated intervention</u>: No information. <u>Comment</u>: 11% of children in the control group received BCG during follow-up.	When a death was identified a standard verbal autopsy was conducted by a clinician about 3 months after the death. <u>Blinding of outcome assessors</u>: Probably not, but objective outcome.	<u>Missing data</u>: "Of the 2343 children enrolled in the trial between November 2004 and March 2008, 23 were excluded (Figure 1). The remaining 2320 children were included in the main analysis" <u>Analysis</u>: Cox proportional hazards model with age as underlying time.

Low risk of bias due to confounding
Low risk of performance bias due to deviations from intended interventions
Low risk of detection bias in measurement of outcomes
Low risk of attrition bias due to missing outcome data
Unclear risk of bias in selection of the reported result
Overall: Low risk of bias

Also from this cohort

Boys vs. girls: Boys RR=0.59 (0.32, 1.11); Girls RR=0.51 (0.25, 1.06); from text on page 248[16]

Vitamin A supplementation: Table 4 (computed from MRRs)[17]

USA c.1935[26]

Result selected	Sample	Allocation or randomization	Blinding and co-interventions	Ascertainment of mortality	Data & analysis
**MRR (unadjusted) = 0.91 (0.41, 1.99) Computed from rates in Table 9, aged 0-4 years**	Randomized trial of BCG vs. no BCG. 3008 children attending the Indian Service schools, followed-up for up to a possible 11 years (interrupted by WWII). Selected for inclusion in review: 846 age under 5.	Allocation: "a record was prepared for each person who failed to react to tuberculin PPD. All of these records for each school an adjacent area were then sorted b sex and year of birth. An alternate division of the records was then made within each sex and age group. Approximately one-half received the BCG vaccine while the remaining number served as controls. In a small number of instances, the person selected was absent from school and one of those listed for the control group was substituted and the absentee then served as a control."	Blinding of participants: Probably not. "At the same time that the vaccine was given, the control group received an intracutaneous injection of 0.1 cc physiological saline." However children receiving BCG would develop a reaction. Blinding of vaccine administrators: No information. Co-interventions and departures from allocated intervention: No information. "Neither vaccinated nor controls were isolated before or after vaccination, nor was their mode of living modified."	No details. Blinding of outcome assessors: Probably not, but objective outcome.	Missing data: Virtually all children followed for 6 years. There were variations after this due to World War II; attempts made to collect data up to 11 years later but numbers are much lower for later years. Analysis: Unadjusted comparison of counts of deaths.

Moderate risk of bias due to confounding (absentees allocated to the control arm)
Moderate risk of performance bias due to deviations from intended interventions (any blinding unlikely to be maintained; no information on co-interventions)
Low risk of detection bias in measurement of outcomes
Low risk of attrition bias due to missing outcome data
Unclear risk of bias in selection of the reported result
Overall: Moderate risk of bias (lack of randomization, blinding and no information on co-intervention)

USA c.1941[27]

Result selected	Sample	Allocation or randomization	Blinding and co-interventions	Ascertainment of mortality	Data & analysis
**Risk ratio (unadjusted) = 0.42 (0.13, 1.35) Computed from counts in Table 14A**	Randomized trial of BCG vs. no BCG. 451 children of expectant mothers with tuberculosis or where tuberculosis was present in the immediate household. Followed up to 13 years. 231 vaccinated and 220 controls. BCG was given at different times depending on subgroup. Babies were placed in state approved foster homes until approximately 6-8 weeks old, this varied based on the above factors.<br>Selected for inclusion in review: All.	Allocation: "In each subgroup… the children were randomly drawn and alternately assigned to vaccinated or control groups. Thus, the randomized drawing was independent of the process of classifying into subgroups. The formation of the subgroup before alternation reduces the size of population required to ensure unbiased vaccinated and control groups. This alternation was done on a master chart in the main office by a physician who did not do the field work." No significant differences found between vaccine and control groups with the exception of birth weight when split into 13 weight intervals. When regrouped into 3 groups, no significant difference found.	Blinding of participants: "In the nonvaccinated, the same procedure was followed except that saline was used instead of the vaccine (placebo). However children receiving BCG would develop a reaction.<br>Blinding of vaccine administrators: No information.<br>Co-interventions and departures from allocated intervention: BCG was administered at different times depending on whether child was removed at birth/ later and the level of TB risk. It ranged from birth to 3 months old.<br>"There were no appreciable differences in the vaccinated and control groups in any of the above categories" [home visits, roentgenograms, examinations by physicians].	Visited weekly while in foster home. Every 6 months they returned to the clinic.<br>Blinding of outcome assessors: Probably not, but objective outcome.	Missing data: Results table imply deaths known for all participants. However, "some 23 per cent were not followed for the specified time in both groups… There were no significant differences in the total of vaccinated and control subjects lost from the study. However, there were significant differences between the groups in regard to reason for loss. This was due to an excess of vaccinated lost because of delinquency or moving and an excess of controls lost for 'other' reasons". It is not clear whether these are counted in the denominator when presenting mortality data.<br>Analysis: Unadjusted comparison of counts of deaths.

Moderate risk of bias due to confounding (alternation with no allocation concealment)
Low risk of performance bias due to deviations from intended interventions
Low risk of detection bias in measurement of outcomes
Moderate risk of attrition bias due to missing outcome data (unclear whether outcomes known for 23% lost to follow up were)
Unclear risk of bias in selection of the reported result
Overall: Moderate risk of bias (no allocation concealment and uncertainty around missing data)

Risk-of-bias assessments for non-randomized studies of BCG vaccine

Bangladesh 1986-2001[1]

Result selected	Sample	Confounders measured (*adjusted for)	Comparability of groups	Ascertainment of vaccine status	Co-interventions	Ascertainment of mortality	Data & analysis
**Hazard ratio (adjusted) = 0.2 (0.07, 0.54) Table 6 (0-60 days)**	Observational comparison. Based on 39,625 children born between Jan 1 1986 and Jan 1 2000. 1731 excluded because they died (1600) or migrated (131) before 42 days (vaccines start at 6 weeks). Analysis of 37,894 children for BCG.<br>Selected for inclusion in review: 37,894 children (including 9704 who received BCG aged 0-60 days).	SES: Maternal education, asset score.<br>Child's health: Distance from hospital, MUAC (not used, too much missing data).<br>Other: Birth order, religion, maternal age.<br>Age: Yes*.<br>Gender: Yes.	Vaccines more likely among: Older mothers, higher education, not first born, higher asset score (least poor), non Muslims, closer to hospital.<br>Mortality more likely among: Older mothers, lower education, first born (DTP)/third born (MCV), lowest asset score.	Frequency: Every 2 weeks until Jan 1998, once a month since then.<br>Method: Monthly immunization days, community worker vaccinates children on this day and records them in record keeping book.<br>Vaccinated: Children given vaccine during immunization day.<br>Unvaccinated: No explicit definition given. Children with no vaccine recorded in book/did not attend vaccination day.<br>Dead children: Status based on vaccines received up to 30 days before death.<br>Approach: Information updated on day vaccine given.	DTP/OPV: High probability of co-administration (55% of BCG given simultaneously with DTP1, 10.1% with DTP2 and 5.5% with DTP3). High probability of differential DTP co-intervention (follow-up 60 months).<br>MCV: High probability of differential MCV co-intervention (follow-up 60 months).<br>Other: No information about any other co-interventions.	All deaths included except those due to trauma or accidents.	Cox proportional hazards model "with time varying covariates with DTP grouped by age at time of BCG vaccination." Reference group not vaccinated with BCG. No confounders included in the model. Reanalysed using 30 day lag period & got consistent results (data not shown). Not clear but looks like mortality is up to 60 months, not adjusted for MCV, unclear about DTP.

Moderate risk of bias in selection of participants into the study
High risk of bias due to confounding (no adjustment for child's health or SES / unadjusted comparison of two groups)
Moderate risk of bias in classification of vaccination status
Very high risk of bias due to deviations from intended interventions (High probability of differential co-intervention of DTP and MCV)
Low risk of bias in measurement of outcomes
Moderate risk of bias due to missing outcome data
Moderate risk of bias in selection of the reported result
Overall: Very high risk of bias (co-intervention with DTP, MCV)

Also from this cohort
DTP with BCG vs. DTP after BCG: HR=0.56 (0.33, 0.97) Table 3 (computed from HRs)[30]
BCG after DTP vs. DTP after BCG: HR=0.88 (0.48, 1.63) Table 3 (computed from HRs)[30]
BCG with or after DTP vs DTP after BCG: MRR=0.66 (0.42, 1.03) Table 3 (computed from rates)[30]
Also from this paper
Age: Table 6 (adjusted results)

Benin 1983-1987[2]

Result selected	Sample	Confounders measured (*adjusted for)	Comparability of groups	Ascertainment of vaccine status	Co-interventions	Ascertainment of mortality	Data & analysis
**Odds ratio (adjusted) = 0.68 (0.38, 1.23) Table 2**	Case-control study. Cases: All children who died between January 1986 and October 1987 and before the age of 3 years. Controls: selected from register of all children in the area, matched for age, sex and village of residence. "Some 74 cases and 230 controls were available for analysis" with 1-4 matched controls per case.<br>"Children who were born and died within the interval of about 3 months between surveys were rarely reported. 2 infants who were known to have died within one month of birth were excluded. All other children had at least attained 4 months of age."<br>Selected for inclusion in review: 143 children who were unvaccinated and 151 children who were vaccinated.	SES: Socioeconomic score* (no details how this is calculated).<br>Child's health: Weight for age*.<br>Other: NR.<br>Age: NR.<br>Gender: NR.	Vaccines more likely among: No information.<br>Mortality more likely among: Lower socioeconomic status.	Frequency: None.<br>Method: Preventive child care cards, kept at the communal health centre.<br>Vaccinated: Children with a vaccine recorded by vaccination team during visits to the village.<br>Unvaccinated: Children with no recorded vaccine.<br>Vaccination card could not be found for 10 children (1 case, 9 controls).	DTP: No information. High probability of differential co-intervention (follow-up 4-35 months).<br>MCV: No information. High probability of differential co-intervention (follow-up 4-35 months).<br>Other: No information about any other co-interventions.	When a child under 3 died the interviewer recorded the symptoms parents mentioned. A medical doctor subsequently visited the household to establish a probable cause of death.	Analysis by conditional logistic regression.

High risk of bias in selection of participants into the study (deaths in first month excluded)
High risk of bias due to confounding (despite matching, some key confounders were not addressed)
Moderate risk of bias in classification of vaccination status
High risk of bias due to deviations from intended interventions (high probability of differential co-intervention of DTP and MCV)
Low risk of bias in measurement of outcomes
Moderate risk of bias due to missing outcome data
Moderate risk of bias in selection of the reported result
Overall: High risk of bias (confounding and subsequent vaccinations)

Burkina Faso 1985-1993[3]

Result selected	Sample	Confounders measured (*adjusted for)	Comparability of groups	Ascertainment of vaccine status	Co-interventions	Ascertainment of mortality	Data & analysis
**Hazard ratio (adjusted) = 0.5 (0.34, 0.75) Table 3 adj results BCG vs. unvaccinated, first visit**	Observational comparison. During 1985 and 1996 9412 births were registered. 204 stillbirths and 123 infants with no recorded month of birth were excluded, leaving 9085 infants. Selected for inclusion in review: Children who survived until first visit (up to 7 months of age).	SES: Area*. Child's health: Child's health service use, diarrhoea*, fever, cough and malnutrition during first year. Other: Age of mother, age of father, number of wives, mode of delivery, dispensary in village*, family health service use*, birth season*, birth period. Age: Yes*. Gender: Yes.	Vaccines more likely among: Boys, children in Yako, older mothers, modern delivery mode, dispensary in village, frequent health service use (child & family), having diarrhoea, fever, cough and malnutrition, being born Mar-Oct, being born after 1988 in Pissila and 1989 in Yako. Mortality more likely among: Boys, Pissila, younger mothers, more wives, modern delivery, no dispensary, frequent child's health service use, rare family health service use, having diarrhoea, fever, cough & malnutrition, born Nov-Feb, birth in 1986-88 in Pissila and 1987-89 in Yako.	Frequency: Every 6 months, although in 1993-5 the average interval was 12 months. Method: Vaccination cards. Vaccinated: Vaccination recorded on vaccination card. Unvaccinated: Children with no vaccine recorded or whose card weren't seen. Dead children: When child died, belongings were discarded including vaccination cards. Approach: Landmark, based on vaccination status at first visit only.	DTP: "Most of the vaccinated children received either BCG followed by DTP or the vaccines simultaneously"; no information on proportions. High probability of differential DTP co-intervention (follow-up 6 months from first visit (which was in the first 6 months of life)). MCV: High probability of differential MCV co-intervention (follow-up 6 months from first visit (which was in the first 6 months of life)). Other: No information about any other co-interventions.	Collected during 6 monthly-annual visits.	Cox proportional hazards model. Follow-up began at first visit (before 7 months). Censoring at 2nd visit, 6 months after 1st visit, out-migration or death.

Very high risk of bias in selection of participants into the study (first visit took place up to 7 months old, so early effects of BCG on mortality not considered)
High risk of bias due to confounding (likely confounding, including by SES)
High risk of bias in classification of vaccination status (children assumed unvaccinated when card not seen)
High risk of bias due to deviations from intended interventions (likely co-interventions including DTP and MCV)
Low risk of bias in measurement of outcomes
Moderate risk of bias due to missing outcome data
Moderate risk of bias in selection of the reported result
Overall: Very high risk of bias (start of follow-up for children up to 7 months old)

Also from this paper

Boys vs. girls: Boys RR=0.42 (0.23, 0.77); Girls RR=0.58 (0.34, 0.98); Table 4 (adjusted results)

Ghana 1998-2004[5]

Result selected	Sample	Confounders measured (*adjusted for)	Comparability of groups	Ascertainment of vaccine status	Co-interventions	Ascertainment of mortality	Data & analysis
**Hazard ratio (adjusted) = 0.18 (0.17, 0.2)**<br>**Table II**	Observational comparison. 17,967 children born between 1st January 1998 and 31st December 2004.<br>Selected for inclusion in review: All.	SES: Mother's education*, poverty status*.<br>Child's health: NR.<br>Other: No<br>Age: Yes*.<br>Gender: No.	Vaccines more likely among: No information.<br>Mortality more likely among: Lower education, higher poverty.	Frequency: Annual collection of vaccination status.<br>Method: Annual survey.<br>Vaccinated: Unclear.<br>Unvaccinated: Unclear.<br>Dead children: No information.<br>Approach: Unclear.<br>Comment: "Annual updates of immunization status and educational attainment and indicators of socioeconomic status are linked to the demographic register."; "Models for the analysis presented here are based on the status of each child at the time of last observation with respect to the WHO definition."	DTP: High probability of differential DTP co-intervention (follow-up to 5 years).<br>MCV: High probability of differential MCV co-intervention (follow-up to 5 years).<br>Other: No information.	All-cause mortality, quarterly visits.	Time conditional HR. No description of any censoring.

Low risk of bias in selection of participants into the study
High risk of bias due to confounding (no adjustment for SES or child's health)
Insufficient information risk of bias in classification of vaccination status (high risk of bias or possibly very high risk of bias: cannot tell how vaccination status was defined; retrospective approach may have been used)
Very high risk of bias due to deviations from intended interventions (high degree of co-intervention with DTP and MCV)
Low risk of bias in measurement of outcomes
Moderate risk of bias due to missing outcome data
Moderate risk of bias in selection of the reported result
Overall: Very high risk of bias (high degree of co-intervention with other vaccines; unable to judge methods for determining vaccination status from publication)

Guinea-Bissau 1984-1987[6]

Result selected	Sample	Confounders measured (*adjusted for)	Comparability of groups	Ascertainment of vaccine status	Co-interventions	Ascertainment of mortality	Data & analysis
**Hazard ratio (adjusted) = 0.63 (0.3, 1.33)** **Text of paper (p. 377)**	Observational comparison, 1984-1987. 1657 infants examined between 2 and 8 months of age. May be children who were never registered as they were not reported during pregnancy and they died or moved before they had the chance to be seen at one of the mobile bi-annual visits. Children followed-up until next visit or 6 months later if next visit was later than that (children presumably aged up to 14 months). BCG coverage increased from 1% and 7% in 1984 and 1985 respectively, to 26% and 29% in 1986 and 1987 respectively. No indication what age BCG recommended. Selected for inclusion in review: All.	SES: Region* Child's health: Weight for age. Other: Season*, period*, DTP* (very few children received DTP after BCG). Age: Yes*. Gender: Yes*.	Vaccines more likely among: No information. Mortality more likely among: No information.	Frequency: 6 monthly Method: BHP records/ vaccination cards. Vaccinated: BHP provided vaccine/saw vaccination card. Unvaccinated: Those who didn't attend initial examinations, children travelling or absent and children examined but too sick to be vaccinated Dead children: No information. Approach: Landmark.	DTP: High probability of differential co-intervention with DTP. MCV: Low probability of differential co-intervention (up to 18% received MCV during 6 months of follow-up). Other: OPV was given with DTP. No information about any of the other co-interventions.	Mortality recorded at 6 monthly visits. If a child did not attend examinations assistants visited the compound to inquire whether the child was travelling, had moved, or died. Cause of death was reviewed and children who died as the result of an accident were censored.	Cox proportional hazards model. Censoring at earliest of 6 months of follow-up, death, migration or new examination.

High risk of bias in selection of participants into the study (appears that follow-up could begin after DTP vaccination)
High risk of bias due to confounding (no adjustment for child's health, potential adjustment for post BCG variable (DTP))
High risk of bias in classification of vaccination status (unvaccinated group may include vaccinated children, and bias towards null from landmark approach)
High risk of bias due to deviations from intended interventions (high rate of subsequent DTP vaccination)
Low risk of bias in measurement of outcomes
Moderate risk of bias due to missing outcome data
Moderate risk of bias in selection of the reported result
Overall: High risk of bias (no adjustment for child's health; selection bias; potential misclassification of vaccination status; co-intervention with DTP)

Guinea-Bissau 1989-2001[7]

Result selected	Sample	Confounders measured (*adjusted for)	Comparability of groups	Ascertainment of vaccine status	Co-interventions	Ascertainment of mortality	Data & analysis
**Hazard ratio (adjusted) = 0.05 (0.01, 0.46) Table 2 vaccinated 1st week vs. unvaccinated at 6 months**	Observational comparison. 22,657 children were born in the area between 1989 and 1999. Birth weight registered for 8481 children born at the hospital. Of these, vaccination status was determined for 7138 children (1343 missing). Only those registered as low birth weight (LBW) were included in the final analyses (N=845). Selected for inclusion in review: 695 LBW children included in the analysis censoring at 6 months.	SES: Maternal schooling*, area*. Child's health: Birth weight*. Other: Mother's age, year of birth*, mother's ethnicity*, civil status, season, day of birth*. Age: Yes*. Gender: Yes*.	Vaccines more likely among: No information. Mortality more likely among: No information.	Frequency: 3 monthly. Method: Registering vaccine at local health centre or vaccination card. Vaccinated: Card seen with vaccine recorded. Unvaccinated: Children with no recorded vaccine, no information on children missing cards. Dead children: No information. Approach: Landmark.	DTP: High probability of differential co-intervention with DTP (followed-up to 6 months). MCV: Low probability of differential co-intervention with DTP (followed-up to 6 months). Other: Nothing reported for any of the co-interventions.	Likely 3 monthly visits.	Cox proportional hazards model. All survival analyses started from age at first examination or 8 days of age, whichever came latest. Children were censored at moving or 6 months of age, whichever came first. Also did separate analyses censoring at the start of the war.

Moderate risk of bias in selection of participants into the study
High risk of bias due to confounding (likely residual confounding despite adjustment)
High risk of bias in classification of vaccination status (likely that unvaccinated group included assumed unvaccinated children)
High risk of bias due to deviations from intended interventions (high degree of co-intervention with DTP)
Low risk of bias in measurement of outcomes
Moderate risk of bias due to missing outcome data
Moderate risk of bias in selection of the reported result
Overall: High risk of bias (co-intervention with DTP; likely residual confounding)

Also from this paper

Boys vs. girls: Boys RR=0.11 (0.02, 0.57); Girls RR=0.24 (0.06, 0.91); footnote to Table 2

Age: Table 2 (computed from MRRs)

Guinea-Bissau 1990-1996[10]

Result selected	Sample	Confounders measured (*adjusted for)	Comparability of groups	Ascertainment of vaccine status	Co-interventions	Ascertainment of mortality	Data & analysis
**Hazard ratio (adjusted) = 0.56 (0.37, 0.84)** **Text of paper**	Observational comparison. 10,298 children born alive of which 686 died, 90 moved and 770 were too young before the first visit. 8752 were alive at first visit and their survival ascertained at the second visit. 8104 were under 7 months at first visit. Selected for inclusion in review: 5274 children aged under 0-6 months at first visit and who either had a vaccination card examined or had no card. Children followed-up until next visit or 6 months later if next visit was later than that (children aged up to 12 months). Results may refer to 4418 children.	SES: Cluster*, maternal education, latrine. Child's health: Maternal tetanus, MUAC, well, birth at home. Other: Length of follow-up*, season, period, maternal age, birth order, ethnicity, previous dead children, DTP*. Age: Yes* . Gender: Yes.	Vaccines more likely among: Children who had more contact with health system, mothers who received tetanus during pregnancy and who gave birth outside the home. They also had larger MUAC, younger mothers, had fewer children, have a latrine and not belong to Balanta or Pepel ethnicity. Mortality more likely among: No information.	Frequency: Not updated. Method: Vaccination card Vaccinated: Children whose card was seen. Unvaccinated: Children who had no date or were declared not to have received the vaccine. Dead children: No information. Approach: Landmark (date vaccination assessed).	DTP: High probability of differential co-intervention with DTP (By 6 months 19 (1%) unvaccinated children had received at least one dose of DTP, 1298 (53%) vaccinated children had received at least one dose of DTP. Followed-up to maximum of 12 months). MCV: Moderate probability of differential co-intervention with MCV (children followed up until to maximum of 12 months, and 791 children received MCV between 7 and 11 months). Other: No information provided for any other co-interventions.	Information on mortality was obtained at subsequent visits (meaning children had to be visited twice to be included in the study).	Cox proportional hazards model. "There was no loss to follow-up because it was always possible to get information on all children from relatives living in the same compound". Analysis repeated excluding children considered unvaccinated because they had no card.

High risk of bias in selection of participants into the study (follow-up begins after BCG vaccinations for some children)
High risk of bias due to confounding (no adjustment for SES and child's health, potential adjustment for post BCG variable (DTP))
High risk of bias in classification of vaccination status (assumed no card meant unvaccinated)
High risk of bias due to deviations from intended interventions (high degree of co-intervention with DTP and moderate risk of co-intervention with MCV)
Low risk of bias in measurement of outcomes
Moderate risk of bias due to missing outcome data
Moderate risk of bias in selection of the reported result
Overall: High risk of bias (late start of follow-up; likely confounding; co-intervention with DTP; vaccination ascertainment)

Also from this paper

Boys vs. girls: Boys RR=0.63 (0.38, 1.04); Girls RR=0.45 (0.23, 0.86); from text on page 1.

India 1987-1989[19]

Result selected	Sample	Confounders measured (*adjusted for)	Comparability of groups	Ascertainment of vaccine status	Co-interventions	Ascertainment of mortality	Data & analysis
**MRR (unadjusted) = 0.6 (0.18, 1.97) Computed based on Table 4 (Groups 1 vs. 0)**	Observational comparison. Live births between December 1987 and November 1989 in 45 contiguous villages. Analysis files from a previous study available for 4138 live births (286 deaths). Of these, 255 records were missing sex, birthday, or exit day, which left 3883 children (282 deaths). Selected for inclusion in review: 3072 unvaccinated children or children with BCG [Groups 0 and I in the paper].	SES: NR. Child's health: Birth weight, current weight, length/ height, common childhood morbidities. Other: NR. Age: Yes. Gender: Yes.	Vaccines more likely among: No information. Mortality more likely among: No information.	Frequency: 3 monthly visits Method: Vaccination card at home or when the child was bought to the clinic for immunization. Vaccinated: Children with a recorded BCG vaccine. Unvaccinated: Children without a recorded vaccine, children whose vaccination card wasn't seen. Dead children: Data were available for 282/286 dead children. Approach: Landmark.	DTP: High probability of DTP co-intervention (none of the children had received DTP; follow-up stopped on receipt of DTP). MCV: Moderate probability of MCV co-intervention (follow-up to 1 year; 25% of the whole cohort received MCV in the first year). Other: No information is provided about any of the co-interventions.	All-cause mortality likely collected at 3 monthly visits at home.	Unadjusted analysis based on mortality rates in first 12 months, follow-up censored at receipt of DTP. But result is not more extreme than an adjusted analysis that also includes children who had BCG after DTP [MRR=0.41, Table 5].

High risk of bias in selection of participants into the study (vaccinated group was selected according to future events (children subsequently DTP vaccinated excluded))
High risk of bias due to confounding (unadjusted comparison of two groups)
High risk of bias in classification of vaccination status (unvaccinated group included those children whose card wasn't seen)
High risk of bias due to deviations from intended interventions (high probability of co-intervention with DTP and MCV)
Low risk of bias in measurement of outcomes
Moderate risk of bias due to missing outcome data
Moderate risk of bias in selection of the reported result
Overall: High risk of bias (no adjustment for age; assumptions about vaccination status)

Also from this paper

DTP with BCG vs. DTP after BCG: MRR=0.23 (0.03, 1.83) Table 4 (computed from rates)
BCG after DTP vs. DTP after BCG: MRR=0.13 (0.01, 2.22) Table 4 (computed from rates)
BCG with or after DTP vs CTP after BCG: HR=0.11 (0.01, 0.91) Table 5

India 1998-2002[20]

Result selected	Sample	Confounders measured (*adjusted for)	Comparability of groups	Ascertainment of vaccine status	Co-interventions	Ascertainment of mortality	Data & analysis
**Hazard ratio (adjusted) = 0.44 (0.29, 0.66) Table 4, model I (No DTP, BCG)**	Observational comparison. Live births between August 1998 and February 2002. Of the 13,294 infants born 11,619 were born alive and enrolled. Of these 10,274 were alive as of 7 days old (meaning approx 1250 died). Selected for inclusion in review: Child-time before receipt of DTP.	SES: Wood fuel, have roof, lease land, own cattle, maternal education, electricity, TV. Child's health: Birth weight. Other: Mother's prior live births, season. Age: Yes*. Gender: Yes.	Vaccines more likely among: Higher birth weight, higher SES (hard roof, electricity, TV, maternal education). Mortality more likely among: Those SES categories associated with lower vaccination coverage (using wood fuel, owning cattle, owning/leasing land).	Frequency: Fortnightly Method: Mother questioned at visits, if mother couldn't remember the vaccination card was checked if in the house. Vaccinated: Children with reported vaccine. Unvaccinated: No reported vaccination, a different code was used for children who received an unknown vaccine. Dead children: No information Approach: Probably retrospective: vaccination information collected retrospectively but not fully clear what vaccination times were used in the analysis. But only a 2-week window between visits.	DTP: Low probability of differential co-intervention (children who had received BCG only). MCV: Low probability of differential co-intervention (follow-up to 6 months). Other: Part of a vitamin A trial. No information about any of the other co-interventions.	Fortnightly visits.	Cox proportional hazards model. Censoring at earliest of death, 6 months of age, loss to follow-up and receipt of first unknown vaccine.

High risk of bias in selection of participants into the study (follow-up begins at 1 week, after some BCG vaccinations)
High risk of bias due to confounding (no adjustment for SES or child's health, likely confounding SES)
High risk of bias in classification of vaccination status (retrospective collection of vaccination data)
Moderate risk of bias due to deviations from intended interventions
Low risk of bias in measurement of outcomes
Moderate risk of bias due to missing outcome data
Moderate risk of bias in selection of the reported result
Overall: High risk of bias (no adjustment for SES or child's health; retrospective collection of vaccination data)

Also from this paper

Boys vs. girls: Boys RR=0.57 (0.33, 0.98); Girls RR=0.38 (0.20, 0.73); Table 4

Vitamin A supplementation: Table 4 (computed from HRs)

India 2006-2011[21]

Result selected	Sample	Confounders measured (*adjusted for)	Comparability of groups	Ascertainment of vaccine status	Co-interventions	Ascertainment of mortality	Data & analysis
**MRR (unadjusted) = 0.12 (0.09, 0.16) Computed from rates in Table 3 (0-5 weeks)**	Observational comparison. Live study births from 1 January 2006 to 31 December 2011 in 28 villages in Ballabgarh. A total of 12,142 births with immunization details were available. A total of 11,390 had complete information on confounders and were included in analyses. Selected for inclusion in review: Time during which most recent vaccine was BCG between 0 and 5 weeks.	SES: Mother's education, father's education, caste, wealth index. Child's health: Access to health care, presence of a health facility in the village. Other: Birth order. Age: Yes. Gender: Yes. All included in analyses.	Vaccines more likely among: No information. Mortality more likely among: No information.	Frequency: Monthly work plan. Method: Fortnightly visits added to computer database. Vaccinated: Recorded as having received BCG as last vaccine based on computerized system. Unvaccinated: Recorded as not having received any vaccines based on computerized system. Dead children: N/A. Approach: Used date of vaccination, although likely to be quite accurate.	DTP: Low probability of differential DTP (follow-up to 6 weeks). MCV: Low probability of differential MCV (follow-up to 6 weeks). Other: No information is provided about any of the co-interventions.	All-cause mortality Visits / computerized system.	Unadjusted mortality ratios. Children included in each group from receipt of vaccine until receipt of another vaccine or any other exit criteria. Exit criteria were 9 months of age, end of study or migration.

Moderate risk of bias in selection of participants into the study
Very high risk of bias due to confounding (unadjusted analysis, with importantly different ages in vaccinated and unvaccinated periods)
Moderate risk of bias in classification of vaccination status
Moderate risk of bias due to deviations from intended interventions
Low risk of bias in measurement of outcomes
Moderate risk of bias due to missing outcome data
Moderate risk of bias in selection of the reported result
Overall: Very high risk of bias (confounding by age)

Also from this paper

Boys vs. girls: Boys RR=0.09 (0.05, 0.15); Girls RR=0.15 (0.10, 0.23); Table 3 (computed from rates)

Malawi 1995-1997[22]

Result selected	Sample	Confounders measured (*adjusted for)	Comparability of groups	Ascertainment of vaccine status	Co-interventions	Ascertainment of mortality	Data & analysis
**Hazard ratio (adjusted) = 0.45 (0.16, 1.23) Table 2B (children present) BCG vs. No BCG**	Observational comparison. Live births between July 1995 and February 1997. The cohort includes approximately 95% of newborn children (N=803). Of these, 36 were stillborn and 16 died during first week (no vaccines). Selected for inclusion in review: 751 children present at monthly anthropometric examinations.	SES: Maternal schooling, district. Child's health: Weight for age, weight for height, twinning. Other: HIV status of mother*, birth order, season of birth, religion, maternal age & present for examination*. Age: Yes*. Gender: Yes.	Vaccines more likely among: No information. Mortality more likely among: No information (but *associated* with HIV infection, twinning, religion, district, travelling and weight for age).	Frequency: Monthly visits up until 18 months, quarterly from 18 to 60 months. Method: Vaccination card, information verified from health centre records. Vaccinated: Vaccine recorded on card/ health centre records. Unvaccinated: Assumed children without evidence of vaccination were unvaccinated. Dead children: No information. Approach: Landmark. Comment: "Almost all children received the vaccines in the planned sequence."	DTP: High probability of differential DTP co-intervention (follow-up to 8 months; censored in analysis). MCV: Low probability of differential MCV co-intervention (follow-up to 8 months). Other: No information provided for any pre-defined co-interventions.	No information, likely collected at monthly visits.	Cox proportional hazards model. Estimates refer to BCG as most recent vaccine. Children were censored at 8 months. Absent children censored in analysis until they were again examined. Small differences in MR for BCG between retrospective and landmark approaches.

Moderate risk of bias in selection of participants into the study
High risk of bias due to confounding (no adjustment for SES or child's health)
High risk of bias in classification of vaccination status (assumptions about non-vaccination)
High risk of bias due to deviations from intended interventions (high probability of co-intervention with DTP and MCV)
Low risk of bias in measurement of outcomes
Moderate risk of bias due to missing outcome data
Moderate risk of bias in selection of the reported result
Overall: High risk of bias (no adjustment for SES or child's health; assumptions about non-vaccination)

Also from this paper

Boys vs. girls: Boys RR=0.44 (0.13, 1.44); Girls RR=0.40 (0.07, 2.15); Table 2B

Papua New Guinea 1989-1994[23]

Result selected	Sample	Confounders measured (*adjusted for)	Comparability of groups	Ascertainment of vaccine status	Co-interventions	Ascertainment of mortality	Data & analysis
**Hazard ratio (adjusted) = 0.17 (0.09, 0.34) Table 5a, 29 days-5 months**	Observational comparison. Children born under demographic surveillance, registered within 60 days of birth and who survived more than 28 days were included. 6665 children born between 1989 and 1994. 2617 were excluded for a number of reasons, 117 died aged <29 days. Thus 4048 were included in survival analysis. <u>Selected for inclusion in review</u>: 3937 who were aged 29 days-5 months.	<u>SES</u>: Region. <u>Child's health</u>: Twin <u>Other</u>: Hep B*, pneumococcal vaccine*, birth order*, birth year, death of older sibling, birth interval from previous sibling, multiple births, mother's age*, propensity score*, DTP*, MCV*. <u>Age</u>: Yes*. <u>Gender</u>: Yes*.	<u>Vaccines more likely among</u>: Children born to mothers less than 23 and older than 35. <u>Mortality more likely among</u>: No information.	<u>Frequency</u>: Monthly clinics but frequent disruptions. Clinic cards bought to office monthly for entry of dates. <u>Method</u>: Clinic cards held by nurses and child's health books kept by mothers. <u>Vaccinated</u>: Vaccination recorded on clinic records. <u>Unvaccinated</u>: No vaccine recorded on clinic cards. <u>Dead children</u>: Cards kept by nurses for 1 year after last attendance, likely to have information for dead children. <u>Approach</u>: Unclear; vaccination information appears to be prospectively recorded for living and dead children.	<u>DTP:</u> High probability of differential DTP co-intervention (follow-up to 6 months). <u>MCV</u>: Low probability of differential MCV co-intervention (follow-up to 6 months). <u>Other</u>: No information about other co-interventions.	Reported during monthly demographic surveillance. Determined by verbal autopsy.	Cox proportional hazards model with propensity score adjustment. All vaccine included in model. Censoring at migration or end of the study period.

High risk of bias in selection of participants into the study (children had to survive to 29 days to be included)
High risk of bias due to confounding (no adjustment for SES or child's health, adjustment for future DTP)
Moderate risk of bias in classification of vaccination status
High risk of bias due to deviations from intended interventions (high probability of co-intervention with DTP)
Low risk of bias in measurement of outcomes
Moderate risk of bias due to missing outcome data
Moderate risk of bias in selection of the reported result
Overall: High risk of bias (no adjustment for SES or child's health, adjustment for future DTP; late start of follow-up)

Also from this paper

BCG with or after DTP vs. DTP after BCG (age 1-5 months): HR=2.01 (0.89, 4.55) Table 3 (computed from rates across boys and girls)
BCG with or after DTP vs. DTP after BCG (age 6-11 months): HR=0.62 (0.24, 1.64) Table 3 (computed from rates across boys and girls)
Boys vs. girls: Boys RR=0.21 (0.10, 0.45); Girls RR=0.11 (0.04, 0.30); Table 3 (computed from rates)

Senegal 1996-1999[24]

Result selected	Sample	Confounders measured (*adjusted for)	Comparability of groups	Ascertainment of vaccine status	Co-interventions	Ascertainment of mortality	Data & analysis
**Hazard ratio (adjusted) = 0.98 (0.5, 1.9) Table 1**	Observational comparison. 4133 children born and registered in study area between Sept 1996 and Dec 1999. 4102 included in the analyses.<br>Selected for inclusion in review: Among 4421 children who followed the WHO strategy or were unvaccinated.	SES: Health centre area*.<br>Child's health: NR<br>Other: Year of vaccination*, season of vaccination*.<br>Age: Yes*.<br>Gender: Yes.	Vaccines more likely among: No information.<br>Mortality more likely among: No information.	Frequency: Every 3 months after mid 1997.<br>Method: Project records and then vaccination card.<br>Vaccinated: All vaccines are provided and recorded by project team until mid 1997. Following this children with a noted vaccine and date were considered vaccinated.<br>Unvaccinated: Those with no recorded vaccine. After mid 1997 this also includes children with no information available.<br>Dead children: Generally no information provided.<br>Approach: Landmark.	DTP: High probability of differential DTP co-intervention (follow-up to 24 months; censored at receipt of DTP).<br>MCV: High probability of differential MCV co-intervention (follow-up to 24 months; censored at receipt of MCV).<br>Other: No information provided for any of the co-interventions.	No information.	Cox proportional hazards model. Censored at 24 months of age, registration of next vaccine, death or migration.

Moderate risk of bias in selection of participants into the study
High risk of bias due to confounding (no adjustment for child's health)
High risk of bias in classification of vaccination status (children with no information included as unvaccinated, and further bias towards null from landmark approach)
High risk of bias due to deviations from intended interventions (high probability of co-intervention with DTP and MCV)
Low risk of bias in measurement of outcomes
Moderate risk of bias due to missing outcome data
Moderate risk of bias in selection of the reported result
Overall: High risk of bias (no adjustment for child's health; co-intervention with DTP and MCV)

Also from this paper
DTP with BCG vs. DTP after BCG: HR=0.51 (0.25, 1.07) Table 1 (computed from HRs)
BCG after DTP vs. DTP after BCG: HR=0.52 (0.07, 4.05) Table 1 (computed from HRs)
BCG with or after DTP vs. DTP after BCG: HR=0.56 (0.27, 1.15) Table 1 (computed from HRs)
Boys vs. girls: Boys RR=0.92 (0.41, 2.08); Girls RR=0.65 (0.21, 2.02); Table 1 (computed from rates)

Risk-of-bias assessments for non-randomized studies of DTP vaccine

Bangladesh 1986-2001[30]

Result selected	Sample	Confounders measured (*adjusted for)	Comparability of groups	Ascertainment of vaccine status	Co-interventions	Ascertainment of mortality	Data & analysis
**Hazard ratio (adjusted) = 0.52 (0.31, 0.87) Table 3**	Observational comparison. Based on children born between 1986 and 1999. 37,894 children were followed between 6 weeks and 9 months of age. Selected for inclusion in review: Children who received BCG only (N=670 by subtraction; top row of Figure 2) versus children who received BCG followed by DTP1 (N=5740).	SES: Maternal education*, asset score*. Child's health: Distance from hospital. Other: Birth order*, religion*, maternal age*, Period (year of birth). Age: Yes*. Gender: Yes*. BCG: Yes (all children had received BCG).	Vaccines more likely among: Higher education. Mortality more likely among: Lower education, lowest asset score.	Frequency: Every 2 weeks until Jan 1998, once a month since then. Method: Monthly immunization days, community worker vaccinates children on this day and records them in record-keeping book. Vaccinated: Children with a recorded BCG and DTP1 vaccination. Unvaccinated: Children with recorded BCG vaccine but no recorded DTP1 vaccine. Dead children: No information. Approach: Information updated on day vaccine given.	BCG: Low probability of differential BCG co-intervention (only included children who received DTP after BCG). OPV: OPV nearly always given with DTP. MCV: Low probability of differential MCV co-intervention (follow-up to 9 months). Censoring at MCV. Other: No information about any other co-interventions.	Cause of death ascertained by verbal autopsy. Twenty of the 712 deaths were due to accidents; these deaths have been excluded from the analysis.	Cox proportional hazards model with time varying covariates. Censored deaths due to trauma or accidents, and at date of MCV if received before 9 months. Repeated analysis without 30 day lag period & got consistent results (data not shown).

Moderate risk of bias in selection of participants into the study
High risk of bias due to confounding (no adjustment for child's health)
Moderate risk of bias in classification of vaccination status
Moderate risk of bias due to deviations from intended interventions
Low risk of bias in measurement of outcomes
Moderate risk of bias due to missing outcome data
Moderate risk of bias in selection of the reported result
Overall: High risk of bias (no adjustment for child's health)

Also from this paper

DTP with BCG vs. DTP after BCG: HR=0.56 (0.33, 0.97) Table 3 (computed from HRs)
BCG after DTP vs. DTP after BCG: HR=0.88 (0.48, 1.63) Table 3 (computed from HRs)
BCG with or after DTP vs DTP after BCG: MRR=0.66 (0.42, 1.03) Table 3 (computed from rates)[30]
Boys vs. girls: Boys RR=0.84 (0.38, 1.85); Girls RR=0.36 (0.18, 0.72); Suppl. Table 1 (computed from rates)

Benin 1983-1987[2]

Result selected	Sample	Confounders measured (*adjusted for)	Comparability of groups	Ascertainment of vaccine status	Co-interventions	Ascertainment of mortality	Data & analysis
**Odds ratio (adjusted) = 2.2 (0.93, 5.22) Table 2**	Case-control study. Cases: All children who died between January 1986 and October 1987 and before the age of 3 years. Controls: selected from register of all children in the area, matched for age, sex and village of residence. "Some 74 cases and 230 controls were available for analysis" with 1-4 matched controls per case. "Children who were born and died within the interval of about 3 months between surveys were rarely reported. 2 infants who were known to have died within one month of birth were excluded. All other children had at least attained 4 months of age."<br>Selected for inclusion in review: 91 children who were unvaccinated and 41 children who had 1 DTP dose.	SES: Socioeconomic score* (no details how this is calculated).<br>Child's health: Weight for age*.<br>Other: NR.<br>Age: Yes (matched*).<br>Gender: Yes (matched*).<br>BCG: Yes.	Vaccines more likely among: No information.<br>Mortality more likely among: Lower socioeconomic status.	Frequency: None.<br>Method: Preventive child care cards, kept at the communal health centre.<br>Vaccinated: Children with a vaccine recorded by vaccination team during visits to the village.<br>Unvaccinated: Children with no recorded vaccine. Vaccination card could not be found for 10 children (1 case, 9 controls).	BCG: No information on co-administration.<br>OPV: Co-administered with DTP.<br>MCV: No information. High probability of differential co-intervention (follow-up 4-35 months).<br>Other: No information provided for any other co-interventions.	When a child under 3 died the interviewer recorded the symptoms parents mentioned. A medical doctor subsequently visited the household to establish a probable cause of death.	Analysis by conditional logistic regression.

Moderate risk of bias in selection of participants into the study
High risk of bias due to confounding (despite matching, some key confounders were not addressed)
Moderate risk of bias in classification of vaccination status
High risk of bias due to deviations from intended interventions (likely co-interventions including MCV)
Low risk of bias in measurement of outcomes
Moderate risk of bias due to missing outcome data
Moderate risk of bias in selection of the reported result
Overall: High risk of bias (likely confounding; unknown BCG co-administration; likely co-interventions including MCV)

Burkina Faso 1985-1993[3]

Result selected	Sample	Confounders measured (*adjusted for)	Comparability of groups	Ascertainment of vaccine status	Co-interventions	Ascertainment of mortality	Data & analysis
**Hazard ratio (adjusted) = 1 (0.6, 1.67)**<br>**Table 3 (vaccination status at first visit, ratio of BCG and DTP to BCG)**	Observational comparison. During 1985 and 1993, 9412 births were registered. 204 stillbirths and 123 infants with no recorded month of birth were excluded leaving 9085 infants.<br>Selected for inclusion in review: Children who survived until first visit (up to 7 months of age).	SES: Area*.<br>Child's health: Child's health service use, diarrhoea*, fever, cough and malnutrition during first year.<br>Other: Age of mother, age of father, number of wives, mode of delivery, dispensary in village*, family health service use*, birth season*, birth period.<br>Age: Yes*.<br>Gender: Yes.<br>BCG: All received BCG, but not clear when.	Vaccines more likely among: Boys, children in Yako, older mothers, modern delivery mode, dispensary in village, frequent health service use (child & family), having diarrhoea, fever, cough & malnutrition, being born Mar-Oct, being born after 1988 in Pissila and 1989 in Yako.<br>Mortality more likely among: Boys, Pissila, younger mothers, more wives, modern delivery, no dispensary, frequent child's health service use, rare family health service use, having diarrhoea, fever, cough & malnutrition, born Nov-Feb, birth in 1986-88 in Pissila and 1987-89 in Yako.	Frequency: Every 6 months although in 1993-5 the average interval was 12 months.<br>Method: Vaccination cards<br>Vaccinated: Vaccination recorded on vaccination card.<br>Unvaccinated: Children with no vaccine recorded or whose card weren't seen.<br>Dead children: When child died, belongings were discarded including vaccination cards.<br>Approach: Landmark, based on vaccination status at first visit only.	BCG: "Most of the vaccinated children received either BCG followed by DTP or the vaccines simultaneously"; no information on proportions.<br>OPV: No information.<br>MCV: High probability of differential MCV co-intervention (follow-up 6 months from first visit (which was in the first 6 months of life)).<br>Other: No information.	Collected during 6-monthly visits.	Cox proportional hazards model.<br>Follow-up began at first visit (before 7 months).<br>Censoring at 2nd visit, 6 months after 1st visit, out-migration or death.

High risk of bias in selection of participants into the study (children had to survive to first visit to be included)
High risk of bias due to confounding (likely confounding, including by SES)
High risk of bias in classification of vaccination status (children assumed unvaccinated when card not seen)
High risk of bias due to deviations from intended interventions (some received BCG simultaneously; likely co-interventions including MCV)
Low risk of bias in measurement of outcomes
Moderate risk of bias due to missing outcome data
Moderate risk of bias in selection of the reported result
Overall: High risk of bias (potential selection bias; co-administration of BCG; assumptions about non-vaccination; likely con-intervention with MCV)

Also from this paper

Boys vs. girls: Boys RR=1.29 (0.61, 2.71); Girls RR=0.81 (0.39, 1.66); Table 4 (computed from RRs)

Ghana 1984-1991[32]

Result selected	Sample	Confounders measured (*adjusted for)	Comparability of groups	Ascertainment of vaccine status	Co-interventions	Ascertainment of mortality	Data & analysis
**Hazard ratio (adjusted) = 2.39 (0.82, 6.99) Computed from rates in Table 5 (row 3. vs. row 2.)**	Observational comparison. Children enrolled into placebo arm of a vitamin A trial (N=6882). 3330 children aged 6-35 months; 3082 included in analysis (excluding those with no information on health card, or health card known to exist but not seen).<br>Selected for inclusion in review: 665 children who had health cards confirming receipt of DTP or no DTP (and no MCV) [Groups 2 and 3 in the paper].	SES: Zone* and radio in compound*.<br>Child's health: Ever breastfed, still breastfeeding, MUAC, measles before enrolment, previously admitted to hospital, weight for age*.<br>Other: NR.<br>Age: Yes*.<br>Gender: Yes.<br>BCG: BCG scar.	Vaccines more likely among: Children still breastfeeding, lower weight for age children and children more likely to have been hospitalized.<br>Mortality more likely among: No information.	Frequency: Every 4 months.<br>Method: Vaccination cards.<br>Vaccinated: Children whose card was seen and had received DTP.<br>Unvaccinated: Children whose card was seen and had not received DTP.<br>Dead children: Vaccination status not updated for children who died between visits.<br>Approach: Landmark.<br>Comment: There is also a 'no health card' group, which could be treated as an unvaccinated group. However. "15% of children without a health card had a BCG scar. These children may have lost the card or never been issued one."	BCG: Co-administered with DTP in 76% of children.<br>OPV: "Most children had registered an OPV at the same time as a DTP vaccine".[31]<br>MCV: High probability of differential MCV co-intervention (86% received MCV either simultaneously with DTP or before DTP).<br>Other: Many missing vaccines given in first 4 months of enrolment (50% additional DTP, 50% MCV), although these appear evenly balanced between groups. Only included children who had received placebo in vitamin A trial. No information provided about any of the other co-interventions.	Deaths were identified through visits and independently by key informants based in the community. Follow-up procedures were independent of the vaccination status of the child.	Cox proportional hazards model. Vaccination status at enrolment was a fixed term variable during follow-up.

Very high risk of bias in selection of participants into the study (included children were 6-35 months, so early effects of DTP on mortality not considered)
High risk of bias due to confounding (likely confounding)
Moderate risk of bias in classification of vaccination status
Very high risk of bias due to deviations from intended interventions (high proportion of co-administration with BCG and with MCV)
Low risk of bias in measurement of outcomes
Moderate risk of bias due to missing outcome data
Moderate risk of bias in selection of the reported result
Overall: Very high risk of bias (selection of children into the study; high proportion of co-administration with BCG; high proportion of co-administration with MCV)

Ghana 1998-2004[5]

Result selected	Sample	Confounders measured (*adjusted for)	Comparability of groups	Ascertainment of vaccine status	Co-interventions	Ascertainment of mortality	Data & analysis
**Hazard ratio (adjusted) = 0.15 (0.14, 0.16) Table II, DTP1**	Observational comparison. 17,967 children born between 1st January 1998 and 31st December 2004. Selected for inclusion in review: All.	SES: Mother's education*, poverty status*. Child's health: NR. Other: Mother's age. Age: Yes*. Gender: No. BCG: Yes.	Vaccines more likely among: BCG recipients. Mortality more likely among: Lower education, higher poverty.	Frequency: Annual collection of vaccination status. Method: Annual survey. Vaccinated: Unclear. Unvaccinated: Unclear. Dead children: No information. Approach: Unclear. Comment: "Annual updates of immunization status and educational attainment and indicators of socioeconomic status are linked to the demographic register."; "Models for the analysis presented here are based on the status of each child at the time of last observation with respect to the WHO definition."	BCG: No information. OPV: Usual to be given simultaneously with DTP. MCV: High probability of differential MCV co-intervention (follow-up to 5 years). Other: No information .	All-cause mortality, quarterly visits.	Time conditional HR. No description of any censoring.

Low risk of bias in selection of participants into the study
Very high risk of bias due to confounding (high degree of confounding with BCG)
Insufficient information to assess risk of bias in classification of vaccination status (high risk of bias or possibly very high risk of bias: cannot tell how vaccination status was defined; retrospective approach may have been used)
Very high risk of bias due to deviations from intended interventions (high degree of co-intervention with MCV)
Low risk of bias in measurement of outcomes
Moderate risk of bias due to missing outcome data
Moderate risk of bias in selection of the reported result
Overall: Very high risk of bias (high degree of co-intervention with other vaccines; unable to judge methods for determining vaccination status from publication)

Guinea-Bissau 1984-1987[6]

Result selected	Sample	Confounders measured (*adjusted for)	Comparability of groups	Ascertainment of vaccine status	Co-interventions	Ascertainment of mortality	Data & analysis
**Hazard ratio (adjusted) = 1.92 (1.04, 3.52)**<br>**Text of paper, p. 377**	Observational comparison. 1984-1987, when DTP first introduced into the area. The sample was 1657 infants examined between 2 and 8 months of age (DTP from 3 months although some as early as 2 months). May be children who were never registered as they were not reported during pregnancy and they died or moved before they had the chance to be seen at one of the mobile bi-annual visits. Children followed-up until next visit or 6 months later if next visit was later than that (children presumably aged up to 14 months).Few had previously received BCG.<br>Selected for inclusion in review: All.	SES: Region*.<br>Child's health: Weight for age.<br>Other: Season*, period*.<br>Age: Yes*.<br>Gender: Yes*.<br>BCG: Yes*.	Vaccines more likely among: No information.<br>Mortality more likely among: No information.<br>No DTP tended to have lower weight for age z-scores, which was associated with higher mortality. These scores not used as many unvaccinated children not weighed as they were travelling.	Frequency: 6 monthly.<br>Method: BHP records / vaccination cards.<br>Vaccinated: BHP provided vaccine / saw vaccination card with date of vaccine.<br>Unvaccinated: Those who were examined on days no vaccines were available, children travelling or absent, children examined but too sick to be vaccinated and children who were 2 months old.<br>Dead children: No information.<br>Approach: Landmark.	BCG: No information ("few children had received BCG first, the proportion increasing from 1% to 29% between 1984 and 1987").<br>OPV: Co-administered with DTP from 1984/5.<br>MCV: Low probability of differential co-intervention (up to 18% received MCV during 6 months of follow-up, but proportion appears similar for DTP and no DTP groups).<br>Other: Up to 40% rate of DTP vaccinations were received during the 6 months of follow-up: more in the DTP group. No information on other co-interventions.	Mortality recorded at 6 monthly visits. If a child did not attend examinations, assistants visited the compound to inquire whether the child was travelling, had moved, or died. Cause of death was reviewed and children who died as the result of an accident were censored.	Cox proportional hazards model. Censoring at earliest of 6 months of follow-up, death, migration or new examination.

High risk of bias in selection of participants into the study (follow-up could begin after DTP vaccination)
High risk of bias due to confounding (no adjustment for child's health)
High risk of bias in classification of vaccination status (unvaccinated group may include vaccinated children, and bias towards null from landmark approach)
High risk of bias due to deviations from intended interventions (high rate of subsequent DTP vaccinations)
Low risk of bias in measurement of outcomes
Moderate risk of bias due to missing outcome data
Moderate risk of bias in selection of the reported result
Overall: High risk of bias (no adjustment for child's health; assumptions about non-vaccination)

Also from this paper

Boys vs. girls: Boys RR=1.56 (0.70, 3.48); Girls RR=2.34 (1.04, 5.27); from text on page 377.

Guinea-Bissau 1989-2001[33]

Result selected	Sample	Confounders measured (*adjusted for)	Comparability of groups	Ascertainment of vaccine status	Co-interventions	Ascertainment of mortality	Data & analysis
**Hazard ratio (adjusted) = 1.58 (0.36, 7.02)** **Text of paper, p. 18**	Observational comparison. 774 children aged 5 weeks to 5 months of whom 313 had vaccination card inspected in preceding 3 months. Of 2065 aged 6-20 months, of whom 1178 had their card inspected, 220 had not received MCV. Selected for inclusion in review: All children aged 5 weeks-20 months who had not received MCV (N=533).	SES: Ownership of pigs. Child's health: Breastfeeding, infections, hospitalization, arm circumference. Other: Living with mother. Age: Yes*. Gender: Yes*. BCG: Virtually all children vaccinated with BCG.	Vaccines more likely among: No information. Mortality more likely among: No information.	Frequency: Every 3 months. Method: Vaccination cards & local health centre records. Vaccinated: Vaccine recorded on vaccination card, registration of vaccine at local health centre. Unvaccinated: No vaccine recorded on vaccination card, registration of vaccine at local health centre. Dead children: No information. Approach: Landmark, using vaccination status at the last home visit in the 3 months before the start of the war.	BCG: No information. OPV: Co-administrated with DTP. MCV: Low probability of differential MCV co-intervention (low vaccination rate during war period). Other: No information provided about any other co-interventions.	Evaluated from onset of war (7 June 1998) until 31 August 1998. Information obtained when routine surveillance was resumed in Sept 1998. Reviewed verbal autopsies to establish cause of death.	Cox proportional hazards model. No information about censoring.

Very high risk of bias in selection of participants into the study (follow-up begins after DTP vaccinations, and restriction to sample determined by subsequent MCV)
High risk of bias due to confounding (no adjustment for SES or child's health)
High risk of bias in classification of vaccination status (children without vaccination card assumed unvaccinated)
Moderate risk of bias due to deviations from intended interventions
Moderate risk of bias in measurement of outcomes
Moderate risk of bias due to missing outcome data
Moderate risk of bias in selection of the reported result
Overall: Very high risk of bias (follow-up begins after DTP vaccinations, and restriction to sample determined by subsequent MCV)

Also from this paper

Boys vs. girls: Boys RR=0.21 (0.01, 3.34); Girls RR=2.33 (0.14, 39.16); Table 2 (computed from counts)

Guinea-Bissau 1990-1996[10]

Result selected	Sample	Confounders measured (*adjusted for)	Comparability of groups	Ascertainment of vaccine status	Co-interventions	Ascertainment of mortality	Data & analysis
**Hazard ratio (adjusted) = 1.74 (1.1, 2.75)** **Text of paper**	Observational comparison. 10,298 children born alive of whom 686 died, 90 moved, and 770 were too young before the first visit. 8752 were alive at first visit and their survival ascertained at the second visit. 8104 were under 7 months at first visit. DTP given from 6 weeks. Selected for inclusion in review: 5274 children aged under 0-6 months at first visit and who either had a vaccination card examined or had no card. Children followed-up until next visit or 6 months later if next visit was later than that (children aged up to 12 months). Results may refer to 4418 children.	SES: Cluster*, maternal education, latrine. Child's health: MUAC, well, birth at home. Other: Season, period, maternal tetanus, maternal age, birth order, ethnicity, previous dead children, length of follow-up*. Age: Yes*. Gender: Yes. BCG: Yes*.	Vaccines more likely among: Children who had more contact with health system, mothers who received tetanus during pregnancy and who gave birth outside the home. They also had larger MUAC, younger mothers, had fewer children, have a latrine and not belong to Balanta or Pepel ethnicity. Mortality more likely among: No information.	Frequency: Visits every 5-7 months on average. Method: Vaccination card. Vaccinated: Children whose card was seen and vaccine recorded. Unvaccinated: Children who had no date or were declared not to have received the vaccine. Also children with no card. Dead children: No information. Approach: Landmark. Only vaccination status at first visit used (aged 0-6 months).	BCG: No information. OPV: Almost always co-administered with DTP. MCV: Moderate probability of differential co-intervention with MCV (children potentially followed up to maximum of 12 months, and 791 children received MCV between 7 and 11 months). Other: No information provided for any other co-interventions.	Information on mortality was obtained at subsequent visits (meaning children had to be visited twice to be included in the study).	Cox proportional hazards model. "There was no loss to follow-up because it was always possible to get information on all children from relatives living in the same compound". Analysis repeated excluding children considered unvaccinated because they had no card.

High risk of bias in selection of participants into the study (follow-up begins after DTP vaccinations)
High risk of bias due to confounding (likely confounding not adjusted for)
High risk of bias in classification of vaccination status (assumed no card meant unvaccinated)
High risk of bias due to deviations from intended interventions (likely MCV co-intervention)
Low risk of bias in measurement of outcomes
Moderate risk of bias due to missing outcome data
Moderate risk of bias in selection of the reported result
Overall: High risk of bias (likely confounding; assumptions about non-vaccination; co-intervention with MCV)
Also from this paper
Boys vs. girls: Boys RR=1.45 (0.81, 2.59); Girls RR=2.31 (1.16, 4.59); from text on page 1. [10]

Guinea-Bissau 1996-2002[38]

Result selected	Sample	Confounders measured (*adjusted for)	Comparability of groups	Ascertainment of vaccine status	Co-interventions	Ascertainment of mortality	Data & analysis
**Risk ratio (adjusted) = 3.45 (1.30, 9.09) Table 1**	Observational comparison. 3133 children admitted to paediatric hospital from May 2001 to April 2002.<br>Selected for inclusion in review: 719 included in the analysis (excluding those who had a measles vaccine).	SES: Home district.<br>Child's health: Weight for age.<br>Other: Examining clinician, season.<br>Age: Yes*.<br>Gender: No.<br>BCG: All children vaccinated with BCG.	Vaccines more likely among: Those with higher weight for age.<br>Mortality more likely among: No information.	Frequency: During hospital visit.<br>Method: Vaccination cards and interview with mother or guardian.<br>Vaccinated: Vaccine recorded on vaccination card, or combination of interview and evidence on child's body.<br>Unvaccinated: No vaccine recorded on vaccination card, or combination of interview and lack of evidence on child's body.<br>Dead children: Some children died quickly without vaccination status being ascertained (excluded from analysis).<br>Approach: Landmark, using vaccination status at arrival to hospital.	BCG: All children with vaccination cards had been vaccinated.<br>OPV: Co-administrated with DTP.<br>MCV: No MCV co-intervention (excluded from the analysis).<br>Other: No information provided about any other co-interventions.	Observed during the hospital stay.	Mantel-Haenszel method, stratified by age and number of doses.

Very high risk of bias in selection of participants into the study (restriction to hospitalized sample, follow-up begins after DTP vaccinations, and restriction to sample determined by subsequent MCV)
High risk of bias due to confounding (limited adjustment for SES or child's health)
High risk of bias in classification of vaccination status (children without vaccination card excluded)
Moderate risk of bias due to deviations from intended interventions
Low risk of bias in measurement of outcomes
Moderate risk of bias due to missing outcome data
Moderate risk of bias in selection of the reported result
Overall: Very high risk of bias (restriction to hospitalized sample, follow-up begins after DTP vaccinations, and restriction to sample determined by subsequent MCV)

Also from this cohort[37]
DTP with MCV vs. MCV after DTP: RR=1.95 (1.12, 3.42) Table 3 (computed from counts)
DTP after MCV vs. MCV after DTP: RR=1.52 (0.71, 3.21) Table 3 (computed from counts)
DTP with or after MCV vs. MCV after DTP: RR=1.72 (0.89, 3.34). Obtained as arithmetic mean of the two results above (on the log scale)

Guinea-Bissau 2002-2008[15]

Result selected	Sample	Confounders measured (*adjusted for)	Comparability of groups	Ascertainment of vaccine status	Co-interventions	Ascertainment of mortality	Data & analysis
**Hazard ratio (adjusted) = 4.33 (1.54, 12.2)** **Table 3 (DRR, BCG at birth)**	Observational comparison. Low birth weight cohort examining early vs. late BCG. 2343 children randomized in original trial. Of these, 1855 children were seen at 2 months and card was seen for 1830 children (99%). Selected for inclusion in review: 935 children who received BCG at birth and whose card was seen at 2 month visit.	SES: Mother's education. Child's health: Birth weight, twinning, breastfeeding, MUAC*, Ballard score, weight gain, weight at 2 months, weight for age, length, height for age, head circumference & abdominal circumference. Other: Study area, birth order, consultations, mother died, maternal MUAC, mother's age, ethnicity & living with father. Age: Yes*. Gender: Yes*. BCG: All received BCG at birth.	Vaccines more likely among: Older mothers, mothers with more schooling, families with fewer twins and Bandim. "The delayed DTP (unvaccinated) group had significantly lower anthropometric indices suggesting that the healthier children received DTP early". Mortality more likely among: No information.	Frequency: Data only collected at 2 months. Method: Vaccination cards were examined and all vaccine dates were noted. Vaccinated: Children with DTP recorded on card. Unvaccinated: Children with no DTP recorded on card. Didn't include children with no information. Dead children: Asked to see cards of dead children. Approach: Landmark. Only vaccination status at 2 months used.	BCG: Not co-administered. OPV: No information. MCV: Low probability of differential MCV co-intervention ("A total of 13 children received MCV at 4.5-5 months as part of a trial of early MCV"; children censored at MCV). Other: Sample comes from a trial also of vitamin A, so this was balanced. More than two thirds of the children in the no DTP group received DTP during follow-up.	All-cause mortality collected at 3 monthly visits. Verbal autopsy was conducted by a clinician 3 months after death.	Cox proportional hazards model. Censoring at death, MCV, migration or the 6 month visit, whichever came first.

High risk of bias in selection of participants into the study (follow-up begins after DTP vaccinations)
High risk of bias due to confounding (likely confounding from SES and child's health)
Moderate risk of bias in classification of vaccination status
High risk of bias due to deviations from intended interventions (substantial DTP vaccination in the no DTP group)
Low risk of bias in measurement of outcomes
Moderate risk of bias due to missing outcome data
Moderate risk of bias in selection of the reported result
Overall: High risk of bias (likely confounding; DTP co-intervention in the no-DTP group)

Also from this paper

Boys vs. girls: Boys RR=2.48 (0.61, 10); Girls RR=7.18 (1.53, 33.7); Table 3

India 1987-1989[19]

Result selected	Sample	Confounders measured (*adjusted for)	Comparability of groups	Ascertainment of vaccine status	Co-interventions	Ascertainment of mortality	Data & analysis
**MRR (unadjusted) = 1.11 (0.3, 4.12) Computed from Table 4, comparison of groups I and II**	Observational comparison. Live births between December 1987 and November 1989 in 45 contiguous villages. Analysis files from a previous study available for 4138 live births (286 deaths). Of these, 255 records were missing sex, birthday or exit day, which left 3883 children (282 deaths).<br>Selected for inclusion in review: 1723 children with BCG or BCG followed by DTP [Groups I and II in the paper].	SES: NR.<br>Child's health: Birth weight, current weight, length/height, common childhood morbidities.<br>Other: NR.<br>Age: Yes.<br>Gender: Yes.<br>BCG: All received BCG.	Vaccines more likely among: ("Children with the best nutritional status were vaccinated first").<br>Mortality more likely among: NR.<br>Birth weights similar in the two groups.	Frequency: 3 monthly visits.<br>Method: Vaccination card at home or when the child was bought to the clinic for immunization.<br>Vaccinated: Children with a recorded BCG and DTP vaccines.<br>Unvaccinated: Children with a recorded BCG vaccine.<br>Dead children: Data were available for 282/286 dead children.<br>Approach: Landmark.	BCG: No co-administration.<br>OPV: Given simultaneously.<br>MCV: Moderate probability of MCV co-intervention (9% received MCV simultaneously with DTP; follow-up to 1 year; 25% of the whole cohort received MCV in the first year).<br>Other: No information.	All-cause mortality at 3-monthly visits at home.	Unadjusted analysis based on mortality rates in first 12 months.

Moderate risk of bias in selection of participants into the study
Very high risk of bias due to confounding (unadjusted comparison of children of different ages)
Moderate risk of bias in classification of vaccination status
High risk of bias due to deviations from intended interventions (co-administration and co-intervention with MCV)
Low risk of bias in measurement of outcomes
Moderate risk of bias due to missing outcome data
Moderate risk of bias in selection of the reported result
Overall: Very high risk of bias (unadjusted analysis; co-intervention with MCV)

Also from this paper

DTP with BCG vs. DTP after BCG: MRR=0.23 (0.03, 1.83) Table 4 (computed from rates)
BCG after DTP vs. DTP after BCG: MRR=0.13 (0.01, 2.22) Table 4 (computed from rates)
BCG with or after DTP vs CTP after BCG: HR=0.11 (0.01, 0.91) Table 5
DTP with MCV vs. MCV after DTP: HR=4.77 (0.33, 70.18) Table 6
DTP after MCV vs. MCV after DTP: HR=15.89 (2.21, 118.99) Table 6
DTP with or after MCV vs MCV after DTP: HR=9.14 (1.24, 67.3) Text p. 7306

India 1998-2002[20]

Result selected	Sample	Confounders measured (*adjusted for)	Comparability of groups	Ascertainment of vaccine status	Co-interventions	Ascertainment of mortality	Data & analysis
**Hazard ratio (adjusted) = 1.64 (0.87, 3.07) Computed from results in Table 4 , model I (ratio of 'DTP, BCG' to 'No DTP, BCG')**	Observational comparison. Live births between August 1998 and February 2002. Of the 13,294 infants born 11,619 were born alive and enrolled. Of these 10,274 were alive as of 7 days old.<br>Selected for inclusion in review: All child-time after receipt of BCG.	SES: Wood fuel, have roof, lease land, own cattle, maternal education, electricity, TV.<br>Child's health: Birth weight.<br>Other: Mother's prior live births, season.<br>Age: Yes*.<br>Gender: Yes.<br>BCG: All received BCG.	Vaccines more likely among: Higher birth weight, higher SES (hard roof, electricity, TV, maternal education).<br>Mortality more likely among: Those SES categories associated with lower vaccination coverage (using wood fuel, owning cattle, owning/leasing land).	Frequency: Fortnightly.<br>Method: Mother questioned at visits, if mother couldn't remember the vaccination card was checked if in the house.<br>Vaccinated: Children with reported vaccine.<br>Unvaccinated: No reported vaccination, a different code was used for children who received an unknown vaccine.<br>Dead children: No information.<br>Approach: Probably retrospective: vaccination information collected retrospectively but not fully clear what vaccination times were used in the analysis. But only a 2-week window between visits.	BCG: Co-administration unlikely as median time of BCG is 2.7 weeks.<br>OPV: Co-administered with DTP.<br>MCV: Low probability of differential co-intervention (follow-up to 6 months).<br>Other: Part of a vitamin A trial. No information about any of the other co-interventions.	Fortnightly visits.	Cox proportional hazards model. Effect estimate for review obtained using ratio of HRs for "DTP, BCG" vs. "No DTP, BCG".<br>Censoring at earliest of death, 6 months of age, loss to follow-up and receipt of first unknown vaccine.

Moderate risk of bias in selection of participants into the study
High risk of bias due to confounding (no adjustment for SES or child's health, likely confounding by SES)
High risk of bias in classification of vaccination status (retrospective collection of vaccination data)
Moderate risk of bias due to deviations from intended interventions
Low risk of bias in measurement of outcomes
Moderate risk of bias due to missing outcome data
Moderate risk of bias in selection of the reported result
Overall: High risk of bias (likely confounding; retrospective collection of vaccination data)

Also from this paper

Boys vs. girls: Boys RR=1.04 (0.40, 2.69); Girls RR=2.52 (1.04, 6.29); Table 4 (computed from HRs)

Vitamin A supplementation: Table 4 (computed from HRs)

India 2006-2011[21]

Result selected	Sample	Confounders measured (*adjusted for)	Comparability of groups	Ascertainment of vaccine status	Co-interventions	Ascertainment of mortality	Data & analysis
**MRR (unadjusted) = 0.28 (0.2, 0.4) Computed from Table 3 (6 weeks – 8 months), BCG+DTP vs. BCG only**	Observational comparison. Live study births from 1 January 2006 to 31 December 2011 in 28 villages in Ballabgarh. A total of 12,142 births with immunization details were available. A total of 11,390 had complete information on confounders and were included in analyses.<br>Selected for inclusion in review: Child-time during which most recent vaccine was BCG or DTP between 6 weeks and 8 months.	SES: Mother's education, father's education, caste, wealth index.<br>Child's health: Access to health care, presence of a health facility in the village.<br>Other: Birth order.<br>Age: Yes.<br>Gender: Yes.<br>All included in analyses.<br>BCG: Yes.	Vaccines more likely among: No information.<br>Mortality more likely among: No information.	Frequency: Monthly work plan.<br>Method: Fortnightly visits added to computer database.<br>Vaccinated: Recorded as having received DTP as last vaccine based on computerized system (approx. 90% had received BCG).<br>Unvaccinated: Recorded as having received BCG as last vaccine based on computerized system (some received DTP beforehand).<br>Dead children: N/A.<br>Approach: Used date of vaccination, although likely to be quite accurate.	BCG: No co-administration.<br>OPV: No information.<br>MCV: No co-administration. Low probability of differential MCV (follow-up to 8 months; 'censored' at next vaccine).<br>Other: No information.	All-cause mortality from visits/ computerized system.	Unadjusted mortality ratios. Children included in each group from receipt of vaccine until receipt of another vaccine or any other exit criteria. Exit criteria were 3 years of age, end of study or migration. Sensitivity analyses conducted using 2 week lag period.

Moderate risk of bias in selection of participants into the study
Very high risk of bias due to confounding (unadjusted comparison with importantly different ages in BCG and DTP periods)
Moderate risk of bias in classification of vaccination status
Moderate risk of bias due to deviations from intended interventions
Low risk of bias in measurement of outcomes
Moderate risk of bias due to missing outcome data
Moderate risk of bias in selection of the reported result
Overall: Very high risk of bias (confounding, particularly by age)

Also from this paper

Boys vs. girls: Boys RR=0.22 (0.14, 0.35); Girls RR=0.37 (0.22, 0.62); Table 3 (computed from rates)

Malawi 1995-1997[22]

Result selected	Sample	Confounders measured (*adjusted for)	Comparability of groups	Ascertainment of vaccine status	Co-interventions	Ascertainment of mortality	Data & analysis
**Hazard ratio (adjusted) = 3.19 (0.8, 12.8) Table 2 (only children present)**	Observational comparison. Live births between July 1995 and February 1997. The cohort includes approximately 95% of newborn children (N=803). Of these, 36 were stillborn and 16 died during first week (no vaccines). Selected for inclusion in review: Children present at monthly anthropometric examinations.	SES: Maternal schooling, district. Child's health: Weight for age, weight for height, twinning. Other: HIV status of mother*, birth order, season of birth, religion, maternal age & present for examination. Age: Yes*. Gender: Yes. BCG: Yes.	Vaccines more likely among: No information. Mortality more likely among: No information (but *associated* with HIV infection, twinning, religion, district, travelling and weight for age).	Frequency: Monthly visits up until 18 months, quarterly from 18 to 60 months. Method: Vaccination card, information verified from health centre records. Vaccinated: Vaccine recorded on card / health centre records. Unvaccinated: Assumed children without evidence of vaccination were unvaccinated. Dead children: No information but used both methods. Approach: Landmark. Comment: "Almost all children received the vaccines in the planned sequence."	BCG: NR. OPV: Nearly always co-administered. MCV: Low probability of differential MCV co-intervention (follow-up to 8 months). Other: No information provided for any pre-defined co-interventions.	No information, likely collected at monthly visits.	Cox proportional hazards model. Absent children censored in analysis until they were again examined. No information about amount of missing data for dead children in retrospective approach. Large differences in MR for DTP between retrospective and landmark approaches.

Moderate risk of bias in selection of participants into the study
High risk of bias due to confounding (no adjustment for SES or child's health)
High risk of bias in classification of vaccination status (assumptions about non-vaccination)
Moderate risk of bias due to deviations from intended interventions
Low risk of bias in measurement of outcomes
Moderate risk of bias due to missing outcome data
Moderate risk of bias in selection of the reported result
Overall: High risk of bias (no adjustment for SES or child's health; assumptions about non-vaccination)
Also from this paper
DTP with MCV vs. MCV after DTP: HR=5.27 (1.11, 25) page 724
Boys vs. girls: Boys RR=2.06 (0.43, 9.75); Girls RR=5.44 (0.88, 33.7); Table 2B

Papua New Guinea 1989-1994[23]

Result selected	Sample	Confounders measured (*adjusted for)	Comparability of groups	Ascertainment of vaccine status	Co-interventions	Ascertainment of mortality	Data & analysis
**Hazard ratio (adjusted) = 0.48 (0.22, 1.09) Table 6 (DTP1, 29 days-5 months)**	Observational comparison. Children born under demographic surveillance, registered within 60 days of birth, and who survived more than 28 days were included. 6665 children born between 1989 and 1994. 2617 were excluded for a number of reasons. Thus 4048 were included in survival analysis.<br>Selected for inclusion in review: 2788 who had received BCG before 6 months, who survived the first month of life.	SES: Region.<br>Child's health: Twin.<br>Other: Hep B*, pneumococcal vaccine*, birth order, birth year*, death of older sibling, birth interval from previous sibling, multiple births, mother's age*, MCV*, subsquent DTP doses*.<br>Age: Yes*.<br>Gender: Yes.<br>BCG: All children had received BCG but may have been after DTP.	Vaccines more likely among: Children born to mothers less than 23 and older than 35.<br>Mortality more likely among: No information.	Frequency: Monthly clinics but frequent disruptions. Clinic cards bought to office monthly for entry of dates.<br>Method: Clinic cards held by nurses and child's health books kept by mothers.<br>Vaccinated: Vaccination recorded on clinic records.<br>Unvaccinated: No vaccine recorded on clinic cards or no linked clinic card.<br>Dead children: Cards kept by nurses for 1 year after last attendance, likely to have information for dead children.<br>Approach: Unclear; vaccination information appears to be prospectively recorded for living and dead children.	BCG: Co-administration apparent in up to 2185 children who received DTP before or with BCG.<br>OPV: Co-administered with DTP.<br>MCV: Low probability of differential MCV co-intervention (follow-up to 6 months).<br>Other: Pigbel vaccine co-administered with DTP. Hep B introduced at birth. No information about other co-interventions.	Reported during monthly demographic surveillance. Determined by verbal autopsy.	Cox proportional hazards model. Censoring at death, migration, 6 months or the end of the study period.

Moderate risk of bias in selection of participants into the study
High risk of bias due to confounding (no adjustment for SES or child's health)
High risk of bias in classification of vaccination status (assumed unvaccinated if there was no linked clinic card)
High risk of bias due to deviations from intended interventions (co-administration of BCG, pigbel)
Low risk of bias in measurement of outcomes
Moderate risk of bias due to missing outcome data
Moderate risk of bias in selection of the reported result
Overall: High risk of bias (no adjustment for SES or child's health; co-administration of pigbel)

Also from this paper
BCG after DTP vs. DTP after BCG (age 1-5 months): HR=2.01 (0.89, 4.55) Table 3 (computed from rates across boys and girls)
BCG after DTP vs. DTP after BCG (age 6-11 months): HR=0.62 (0.24, 1.64) Table 3 (computed from rates across boys and girls)
Boys vs. girls: Boys RR=0.76 (0.31, 1.86); Girls RR=0.50 (0.13, 2.01); Table 3 (computed from rates)

Philippines 1988-1991[40]

Result selected	Sample	Confounders measured (*adjusted for)	Comparability of groups	Ascertainment of vaccine status	Co-interventions	Ascertainment of mortality	Data & analysis
**Hazard ratio (adjusted) = 0.87 (0.33, 2.29)** **Text of paper (p. 1025)**	Observational comparison. Children aged up to 30 months who had received a BCG vaccine and had complete baseline data (N=14,334/18,964). Selected for inclusion in review: 10,231 were included in the landmark analysis.	SES: Maternal education* and TV & radio ownership*, household cluster*. Child's health: Prenatal care and birth weight*. Other: None. Age: Yes*. Gender: Yes*. BCG: All children had received BCG but may have been with or after DTP.	Vaccines more likely among: Higher maternal education and prenatal care from a nurse/ midwife. As well as being male, low birth weight and no TV ownership. Mortality more likely among: Lower maternal education, TV/radio ownership & low birth weight.	Frequency: Every 6 months. Method: Examination of vaccination card/clinic records. Vaccinated: Children whose card was seen and a vaccine was noted. Unvaccinated: If mother/ caregiver didn't know if child had received vaccine/ no vaccine received. Dead children: Vaccination status was collected post-mortem. Approach: Landmark.	BCG: Probably co-administered with DTP in some children. OPV: Co-administered with DTP . MCV: High probability of differential MCV co-administration (follow-up to 30 months, but censored at receipt of MCV). Other: No information is provided for any of the co-interventions.	Death information was obtained via post mortem interviews.	Cox proportional hazards model. Estimate adjusted by sex obtained as meta-analysis of estimates in boys and girls. Censored at receipt of MCV or receipt of unknown vaccine, 30 months of age, outmigration or study end.

Very high risk of bias in selection of participants into the study (inclusion of children up to 30 months of age at start of follow-up)
High risk of bias due to confounding (likely confounding not adjusted for)
High risk of bias in classification of vaccination status (considered unvaccinated if mother did not know whether vaccinated)
No information on risk of bias due to deviations from intended interventions (MCV received during long follow-up time; insufficient information about co-administration of BCG: claimed to be high in a letter)
Low risk of bias in measurement of outcomes
Moderate risk of bias due to missing outcome data
Moderate risk of bias in selection of the reported result
Overall: Very high risk of bias (selection of children too long after vaccination)

Also from this paper

Boys vs. girls: Boys RR=0.85 (0.25, 2.87); Girls RR=0.96 (0.26, 5.15); from text on page 1025

Senegal 1996-1999[24]

Result selected	Sample	Confounders measured (*adjusted for)	Comparability of groups	Ascertainment of vaccine status	Co-interventions	Ascertainment of mortality	Data & analysis
**Hazard ratio (adjusted) = 1.37 (0.54, 3.47) Computed from results in Table 1 (ratio of DTP1 to 'BCG not yet DTP' (BCG-first group))**	Observational comparison. 4133 children born and registered in study area between Sept 1996 and Dec 1999. 4102 included in the analyses.<br>Selected for inclusion in review: Among 319 children who followed the WHO strategy.	SES: Health centre area*.<br>Child's health: NR<br>Other: Year of vaccination*, season of vaccination*.<br>Age: Yes*.<br>Gender: Yes*.<br>BCG: All children had received BCG.	Vaccines more likely among: No information.<br>Mortality more likely among: No information.	Frequency: Every 3 months after mid 1997.<br>Method: Project records and then vaccination card.<br>Vaccinated: All vaccines are provided and recorded by project team until mid 1997. Following this children with a noted vaccine and date were considered vaccinated.<br>Unvaccinated: Those with no recorded vaccine. After mid 1997 this also includes children with no information available.<br>Dead children: Generally no information provided.<br>Approach: Landmark.	BCG: No co-administration.<br>OPV: Usually co-administered with DTP.<br>MCV: High probability of differential MCV co-intervention (follow-up to 24 months; censored at receipt of MCV).<br>Other: No information provided for any of the co-interventions.	No information.	Cox proportional hazards model. Censored at 24 months of age, registration of next vaccine, death or migration.

Moderate risk of bias in selection of participants into the study
High risk of bias due to confounding (no adjustment for child's health)
High risk of bias in classification of vaccination status (children with no information included as unvaccinated, and further bias towards null from landmark approach)
High risk of bias due to deviations from intended interventions (high probability of MCV)
Low risk of bias in measurement of outcomes
Moderate risk of bias due to missing outcome data
Moderate risk of bias in selection of the reported result
Overall: High risk of bias (no adjustment for child's health)

Also from this paper
DTP with BCG vs. DTP after BCG: HR=0.51 (0.25, 1.07) Table 1 (computed from HRs)
BCG after DTP vs. DTP after BCG: HR=0.52 (0.07, 4.05) Table 1 (computed from HRs)
BCG with or after DTP vs. DTP after BCG: HR=0.56 (0.27, 1.15) Table 1 (computed from HRs)
DTP with MCV vs. MCV after DTP: HR=1.96 (0.95, 4.04) Table 1 (computed from HRs)
DTP after MCV vs. MCV after DTP: HR=2.40 (1.00, 5.75) Table 1 (computed from HRs)
DTP with or after MCV vs. MCV after DTP: HR=2.05 (1.16, 3.62) Table 1 (computed from HRs)
Boys vs. girls: Boys RR=0.42 (0.09, 2.10); Girls RR=3.55 (0.92, 17.57); Table 1 (computed from rates)

Risk-of-bias assessments for randomized trials of measles-containing vaccine (MCV)

Guinea-Bissau 1989-2001(a)[56]

Result selected	Sample	Allocation or randomization	Blinding and co-interventions	Ascertainment of mortality	Data & analysis
**Risk ratio (unadjusted) = 1 (0.2, 4.92)** **Text of paper (p. 825)**	300 children randomised to MCV or IPV at 6 months. 19 moved or died before receiving the 9 month MCV vaccination.[36] Selected for inclusion in review: All 300 children.	Allocation: "Randomisation was done on a per patient basis and was done with a list of random numbers." (Lancet 1997;350:101-5)	Blinding of participants: Control children received IPV. Blinding of vaccine administrators: No information. Co-interventions and departures from allocated intervention: Half the children were randomised to receive either placebo or vitamin A with their vaccine.	No details. Blinding of outcome assessors: Probably not, but objective outcome.	Missing data: No information. Analysis: Not clear.

Unclear risk of bias due to confounding (allocation may be unconcealed and no information about similarity of groups)
Moderate risk of performance bias due to deviations from intended interventions (participants knew vaccination status)
Low risk of detection bias in measurement of outcomes
Unclear risk of attrition bias due to missing data
Unclear risk of bias in selection of the reported result
Overall: Moderate risk of bias (due to knowledge of vaccination status)

Also from this paper

Vitamin A supplementation: from text on page 825 (computed from counts)

Guinea-Bissau 1989-2001(b)[35]

Result selected	Sample	Allocation or randomization	Blinding and co-interventions	Ascertainment of mortality	Data & analysis
**MRR (unadjusted) = 0.94 (0.53, 1.67) Computed from rates in Table 3 (Second two-dose trial; combined across 3+ and 0-2 doses of DTP)**	Randomized trial of MCV at 6 and 9 months vs. IPV at 6 months and MCV at 9 months.<br>All children born between September 1994 and October 2001 and registered before 6 months of age.[35] War started on 7th June 1998, the war ended in May 1999. Mortality was considerably higher during this period.<br>Selected for inclusion in review: Apparently at least 8511 children randomized (Table 1).	Allocation: No details regarding randomisation process and how allocation of MCV v IPV was done.	Blinding of participants: Control children received IPV.<br>Blinding of vaccine administrators: No information.<br>Co-interventions and departures from allocated intervention: No information.	No details.<br>Blinding of outcome assessors: Probably not, but objective outcome.	Missing data: No information.<br>Analysis: Cox proportional hazards model.[35]

Unclear risk of bias due to confounding (no information about allocation process)
Moderate risk of performance bias due to deviations from intended interventions (participants knew vaccination status)
Low risk of detection bias in measurement of outcomes
Unclear risk of attrition bias due to missing data
Unclear risk of bias in selection of the reported result
Overall: Moderate risk of bias (due to knowledge of vaccination status)

Also from this paper

Boys vs. girls: Boys RR=1.01 (0.52, 1.96); Girls RR=0.94 (0.53, 1.67); Table 1 (computed from counts)

Guinea-Bissau 2002-2008[61]

Result selected	Sample	Allocation or randomization	Blinding and co-interventions	Ascertainment of mortality	Data & analysis
**Hazard ratio (adjusted) = 0.67 (0.38, 1.19) Table 2 (4.5-9 months, all children)**	Between August 2003 and April 2007, 6648 children were enrolled, 6417 were included in the main analysis. Half received vaccine at 4.5 months and half received nothing. All children received MCV at 9 months. Selected for inclusion in review: All.	Allocation: "The mothers or guardians were asked to select an envelope with the allocation number that defined to which group the child belonged. We randomised children to one of three equal sized groups by using block randomisation with 24 envelopes per bag. Block randomisation was organised within the whole study area and not by health centre. The data manager, who was not involved in the recruitment of children, prepared bags with 24 numbered envelopes indicating the randomisation group. These numbers could not be seen by the doctor informing and obtaining consent from the mothers or guardians. A new bag was opened only when the previous one was empty." "No major differences existed in the demographic, socioeconomic, and health related background factors between children included in the early two dose measles vaccine arm (group A) and the two groups with measles vaccine at 9 months of age (groups B +C) (table 1)."	Blinding of participants: No, "No placebo was given." Blinding of vaccine administrators: No, "However, the health workers, doctors, and nurses in the paediatric ward and at the health centres do not inspect the vaccination cards of sick children to guide the treatment, and they were not aware of the purpose of the study." Co-interventions and departures from allocated intervention: None apparent.	Visit to health centre at 9 plus 3 monthly home visits. Blinding of outcome assessors: Probably no, although the outcome is objective. "However, the health workers, doctors, and nurses in the paediatric ward and at the health centres do not inspect the vaccination cards of sick children to guide the treatment and they were not aware of the purpose of the study."	Missing data: "The study was not perfectly balanced with respect to drop-out. As we tested specific strains of measles vaccine, children who had received measles vaccine elsewhere before they attended the 9 month vaccination session were censored in the study because the strain and quality of their measles vaccine would not be known. We excluded more children at the 9 month vaccination session in groups B and C than in group A (fig 1)." 231 children were excluded from the main analysis (80 children had had measles infection before enrolment at 4.5 months of age (five died), 131 children were enrolled within 25 days of the third DTP vaccination as opposed to 4 weeks after (seven died), 18 children were enrolled twice (none died); 2 children who had the wrong age recorded; they were both one year older than originally assumed (neither died). "A further 17 children received the wrong type of measles vaccine at 9 months of age and they have been censored in the analysis from the date of this vaccination (none died)." Analysis: Cox proportional hazards model with age as underlying time.

Low risk of bias due to confounding
Moderate risk of performance bias due to deviations from intended interventions (participants knew vaccination status)
Low risk of detection bias in measurement of outcomes
Moderate risk of attrition bias due to missing data (imbalance in drop-out)
Unclear risk of bias in selection of the reported result
Overall: Moderate risk of bias (due to knowledge of vaccination status)

Also from this cohort

Boys vs. girls: Boys RR=0.94 (0.44, 2.01); Girls RR=0.46 (0.19, 1.11);Table 2[61]

Vitamin A supplementation: Table 3 (computed from rates)[62]

Nigeria c.1961[66]

Result selected	Sample	Allocation or randomization	Blinding and co-interventions	Ascertainment of mortality	Data & analysis
**Risk ratio (unadjusted) = 0.41 (0.14, 1.15) Computed from counts in text (page 144)**	Children under-5 from hospital clinic in Ilesha. Over 2000 children between 6 months and 2 years accepted an invitation for vaccination. attended, record cards available for 1962 children. Selected for inclusion in review: All 1962 with record cards at follow-up.	Allocation: "By a system of random numbers, these children were divided into two equal groups".	Blinding of participants: Probably yes, since placebo was apparently used. "The other group was a control one, receiving gamma globulin and inert material identical with that used in the culture of the virus". Blinding of vaccine administrators: Unclear. Another study reported in the paper is described as "This was not a blind study, since the investigators knew which children had received measles vaccine", suggesting that this might not be the case for the current study. Co-interventions and departures from allocated intervention: None apparent.	Not clear. "At the end of the trial period at the Ilesha Hospital 1962 record cards were Available"; "Among the children in this trial there were 17 known deaths". It is not clear whether these two sources are linked. The paper reports that "the follow-up had to be made in a crowded and over-worked clinic". Blinding of outcome assessors: Probably no, although the outcome is objective.	Missing data: Number randomized not reported, but known to be over 2000. It is not clear whether the numbers of deaths relate to the children with record cards or the larger number randomized. Analysis: None.

Unclear risk of bias due to confounding (no information on randomization procedure)
Low risk of performance bias due to deviations from intended interventions
Unclear risk of detection bias in measurement of outcomes
Moderate risk of attrition bias due to missing data (unknown number at start of study)
Unclear risk of bias in selection of the reported result
Overall: Moderate risk of bias

Risk-of-bias assessments for non-randomized studies of measles-containing vaccine (MCV)

Bangladesh 1977-1985[45]

Result selected	Sample	Confounders measured (*adjusted for)	Comparability of groups	Ascertainment of vaccine status	Co-interventions	Ascertainment of mortality	Data & analysis
**Hazard ratio (adjusted) = 0.51 (0.42, 0.62)**<br>**Text of paper (page 110)**	Matched cohort. 9133 children aged 9-60 months in vaccination area received MCV. An unvaccinated match was found (based on month of birth) for 8135 children. The remaining 998 children were not included in the analyses. Although MCV started in March 1982, events (e.g. mortality) were started in October 1982 so survival has only been analysed from October. One unvaccinated child was included twice so the second pair was excluded resulting in 8134 pairs.<br>Selected for inclusion in review: All.	SES: Maternal education*.<br>Child's health: NR.<br>Other: Size of dwelling*, number of siblings*.<br>Age: Yes*.<br>Gender: Yes*. In analyses which included covariates 635 children were excluded due to missing information.<br>BCG: Yes.<br>DTP: Yes.	Vaccines more likely among: No information.<br>Mortality more likely among: No information.<br>BCG: Coverage 48% and 39% in the two areas.<br>DTP: Coverage 50% and 51% in the two areas.	Frequency: N/A.<br>Method: A detailed record keeping system contained details on the vaccination status and date of vaccination of all children aged <5 years in the study area.<br>Vaccinated: Children who lived in a MCV area (blocks A & C) who had already received MCV.<br>Unvaccinated: Children in blocks B & D where no MCV campaign running and who had not received MCV.	DTP: No information on co-administration.<br>Other: No information.	Community health workers visited households every second week and reported to the field assistants of the Demographic Surveillance System.	Cox proportional hazards model for paired survival data. Censoring at moving, end of study.

Moderate risk of bias in selection of participants into the study
High risk of bias due to confounding (phased introduction of vaccines into two areas; matching by birth date only)
Moderate risk of bias in classification of vaccination status
Insufficient information to assess risk of bias due to deviations from intended interventions
Low risk of bias in measurement of outcomes
Moderate risk of bias due to missing outcome data
Moderate risk of bias in selection of the reported result
Overall: High risk of bias (matching by birth date only)

Bangladesh 1986-2001[1]

Result selected	Sample	Confounders measured (*adjusted for)	Comparability of groups	Ascertainment of vaccine status	Co-interventions	Ascertainment of mortality	Data & analysis
**Hazard ratio (adjusted) = 0.93 (0.65, 1.34) Table 5**	Observational comparison. Based on 39,625 children born between Jan 1 1986 and Jan 1 2000. 1731 excluded because they died (1600) or migrated (131) before 42 days (vaccines start at 6 weeks). Selected for inclusion in review: Analysis of 36,650 for MCV (1244 died (712) or migrated (532) before 9 months).	SES: Maternal education*, asset score*. Child's health: Distance from hospital, MUAC (not used, too much missing data). Other: Birth order*, religion*, maternal age*. Age: Yes*. Gender: Yes*. BCG: Yes*. DTP: Yes* (number of DTP vaccinations).	Vaccines more likely among: Older mothers, higher education, not first born, higher asset score (least poor), non Muslims, closer to hospital. Mortality more likely among: Older mothers, lower education, first born (DTP)/third born (MCV), lowest asset score.	Frequency: Every 2 weeks until Jan 1998, once a month since then. Method: Monthly immunization days, community worker vaccinates children on this day and records them in record keeping book. Vaccinated: Children given vaccine during immunization day. Unvaccinated: Children with no vaccine recorded. Dead children: Status based on vaccines received up to 30 days before death. Approach: Information updated on day vaccine given.	DTP: 77% had received 3 doses of DTP by 9 months, this increased to 93% by 60 months. An additional analysis that censored children on receipt of subsequent vaccines gave HR = 0.61 (0.44, 0.85), but is at risk of bias (towards favouring measles vaccine) due to informative censoring. Other: No information.	All deaths included except those due to trauma or accidents.	Cox proportional hazards model with time varying covariates. Censored deaths due to trauma or accidents. Any subsequent DTP that occurred later than usual schedule was included as a covariate in the model. Reanalysed using 30 day lag period & got consistent results (data not shown).

Moderate risk of bias in selection of participants into the study
High risk of bias due to confounding (no adjustment for child's health)
Moderate risk of bias in classification of vaccination status
High risk of bias due to deviations from intended interventions (16% received additional DTP doses after MCV)
Low risk of bias in measurement of outcomes
Moderate risk of bias due to missing outcome data
Moderate risk of bias in selection of the reported result
Overall: High risk of bias (no adjustment for child's health; some co-intervention with DTP)

Benin 1983-1987[2]

Result selected	Sample	Confounders measured (*adjusted for)	Comparability of groups	Ascertainment of vaccine status	Co-interventions	Ascertainment of mortality	Data & analysis
**Odds ratio (adjusted) = 0.36 (0.16, 0.81)**<br>**Table 2, up to 12 months**	Case-control study. Cases: All children who died between January 1986 and October 1987 and before the age of 3 years. Controls: selected from register of all children in the area, matched for age, sex and village of residence. "Some 74 cases and 230 controls were available for analysis" with 1-4 matched controls per case.<br>"Children who were born and died within the interval of about 3 months between surveys were rarely reported. 2 infants who were known to have died within one month of birth were excluded. All other children had at least attained 4 months of age."<br>Selected for inclusion in review: 177 children who were unvaccinated and 75 children who were vaccinated at less than 12 months of age.	SES: Socioeconomic score* (no details how this is calculated).<br>Child's health: Weight for age*.<br>Other: NR.<br>Age: NR.<br>Gender: NR.<br>BCG: Yes.<br>DTP: Yes (by dose).	Vaccines more likely among: No information.<br>Mortality more likely among: Lower socioeconomic status.	Frequency: None<br>Method: Preventive child care cards, kept at the communal health centre.<br>Vaccinated: Children with a vaccine recorded by vaccination team during visits to the village.<br>Unvaccinated: Children with no recorded vaccine.<br>Dead children: N/A.	DTP: No information on co-administration.<br>Other: No information.	When a child under 3 died the interviewer recorded the symptoms parents mentioned. A medical doctor subsequently visited the household to establish a probable cause of death.	Analysis by conditional logistic regression.

Moderate risk of bias in selection of participants into the study
High risk of bias due to confounding (despite matching, some key confounders were not addressed)
Moderate risk of bias in classification of vaccination status
Insufficient information for bias due to deviations from intended interventions
Low risk of bias in measurement of outcomes
Moderate risk of bias due to missing outcome data
Moderate risk of bias in selection of the reported result
Overall: High risk of bias (key confounders were not addressed)
Also from this paper
Age: Table 2

Burundi 1984-1988[49]

Result selected	Sample	Confounders measured (*adjusted for)	Comparability of groups	Ascertainment of vaccine status	Co-interventions	Ascertainment of mortality	Data & analysis
**MRR (unadjusted) = 0.3 (0.17, 0.52) Computed from rates in Table 5**	Observational comparison. Door-to-door census of all households was done in January 1989 to identify children younger than 5. Children born since January 1984 and alive at 1 July 1988 were registered. A total of 1899 children were registered, of these 1442 had vaccination cards. 74% vaccinated between 9 and 23 months.<br>Selected for inclusion in review: All.	SES: NR.<br>Child's health: Measles infection, measles symptoms (fever, rash, cough, runny nose, red eyes, diarrhoea), hospitalized.<br>Other: Duration of outbreak.<br>Age: Yes.<br>Gender: NR.<br>BCG: No.<br>DTP: No.	Vaccines more likely among: No information.<br>Mortality more likely among: No information.	Frequency: None.<br>Method: Child home-based vaccination record.<br>Vaccinated: Children with MCV and date on card.<br>Unvaccinated: Children with no vaccine on card and children with no card.<br>Dead children: Excluded from sample.<br>Approach: Used categorization of children according to previous vaccination or not.	DTP: No information on co-administration.<br>Other: No information.	Deaths since 1 July 1988 were recorded during door to door census. Same households were visited in September 1989 to assess survival of children found during the January census.	Followed-up for 6 months.<br>67 children received a non-measles vaccine after 9 months of age and had missed an opportunity for MCV.

Very high risk of bias in selection of participants into the study (children were enrolled up to age of 5 years, MCV recommended from 9 months)
Very high risk of bias due to confounding (unadjusted analyses, previous vaccinations not considered)
High risk of bias in classification of vaccination status (unvaccinated group includes children assumed unvaccinated)
Insufficient information for risk of bias due to deviations from intended interventions
Low risk of bias in measurement of outcomes
Moderate risk of bias due to missing outcome data
Moderate risk of bias in selection of the reported result
Overall: Very high risk of bias (children were enrolled up to age of 5 years; unadjusted analyses)

DR Congo 1973-1975[50]

Result selected	Sample	Confounders measured (*adjusted for)	Comparability of groups	Ascertainment of vaccine status	Co-interventions	Ascertainment of mortality	Data & analysis
**MRR (unadjusted) = 0.29 (0.09, 0.98) Computed from survival probabilities in Table II (Group 1v vs. Group 2)**	Observational comparison. In May 1974 children born after 1 June 1969 were entered into the study. Up to April 1977 children under 5 continued to be recruited. In total 7092 children were entered into the study and visited. Groups 1 & 2 consisted of children born between September 1974 and October 1975. 306 children in group 1 were invited for vaccine, of these 255 accepted. No other Ns are reported.<br>Selected for inclusion in review: Children in Group 1v (invited for vaccination and accepted) and Group 2 (unvaccinated). Approx 600 children.	SES: None.<br>Child's health: Weight, height, arm circumference (not used).<br>Other: None.<br>Age: NR.<br>Gender: NR.<br>The authors report "there are no gross social differences between the two areas and both areas have the same health services." In addition they report that "their anthropometric data were identical."<br>BCG: No.<br>DTP: No.	Vaccines more likely among: No information.<br>Mortality more likely among: No information.	Frequency: N/A.<br>Method: Invitation and attendance at clinic.<br>Vaccinated: Children who attend an invitation for MCV.<br>Unvaccinated: Children in area with no MCV.<br>Dead children: N/A.<br>Approach: Categorization.	DTP: No information.<br>Other: No information.	3 monthly visits to study area.	Comparison of cumulative survival probabilities, 7-21 months. No data about vaccines during follow-up or if the unvaccinated children were later vaccinated.

Moderate risk of bias in selection of participants into the study
High risk of bias due to confounding (unadjusted analysis of groups)
Moderate risk of bias in classification of vaccination status
Insufficient information to assess risk of bias due to deviations from intended interventions
Low risk of bias in measurement of outcomes
Moderate risk of bias due to missing outcome data
Moderate risk of bias in selection of the reported result
Overall: High risk of bias (unadjusted analysis of groups)

Ghana 1984-1991[32]

Result selected	Sample	Confounders measured (*adjusted for)	Comparability of groups	Ascertainment of vaccine status	Co-interventions	Ascertainment of mortality	Data & analysis
**Hazard ratio (adjusted) = 0.51 (0.29, 0.97) Table 4 (MRR MCV/DTP all children)**	Observational comparison. Children enrolment into placebo arm of a vitamin A trial (N=6882). 3330 children aged 6-35 months; 3082 included in analysis (excluding those with no information on health card, or health card known to exist but not seen).<br>Selected for inclusion in review: 1793 children who had health cards confirming receipt of MCV (plus DTP) or no MCV (plus DTP) [Groups 3, 4, 5 and 6 in the paper].	SES: Zone* and radio in compound*.<br>Child's health: Ever breastfed, still breastfeeding, MUAC, measles before enrolment, previously admitted to hospital, weight for age*.<br>Other: NR.<br>Age: Yes*.<br>Gender: Yes.<br>BCG: BCG scar.<br>DTP: Yes (all had received at least 1 dose of DTP).	Vaccines more likely among: Children still breastfeeding, lower weight for age children and children more likely to have been hospitalized.<br>Mortality more likely among: No information.	Frequency: Every 4 months.<br>Method: Vaccination cards.<br>Vaccinated: Children whose card was seen and had received DTP and MCV.<br>Unvaccinated: Children whose card was seen and had received DTP.<br>Dead children: Vaccination status not updated for children who died between visits.<br>Approach: Landmark.<br>Comment: There is also a 'no health card' group, which could be treated as an unvaccinated group. However. "15% of children without a health card had a BCG scar. These children may have lost the card or never been issued one."	DTP: 86% of children who had received DTP & MCV received them simultaneously or received at least one dose of DTP after MCV.<br>Other: Many missing vaccines given in first 4 months of enrolment (50% additional MCV), although these appear evenly balanced between groups. Only included children who had received placebo in vitamin A trial. No information provided about any of the other co-interventions.	Deaths were identified through visits and independently by key informants based in the community. Follow-up procedures were independent of the vaccination status of the child.	Cox proportional hazards model. Vaccination status at enrolment was a fixed term variable during follow-up.

Moderate risk of bias in selection of participants into the study
High risk of bias due to confounding (likely residual confounding)
Moderate risk of bias in classification of vaccination status
Very high risk of bias due to deviations from intended interventions (high proportion of co-administration with DTP)
Low risk of bias in measurement of outcomes
Moderate risk of bias due to missing outcome data
Moderate risk of bias in selection of the reported result
Overall: Very high risk of bias (likely residual confounding)
Also from this paper
Boys vs. girls: Boys RR=0.50 (0.19, 1.31); Girls RR=0.52 (0.22, 1.23); Table 4 (computed from rates)

Ghana 1994-1999[28]

Result selected	Sample	Confounders measured (*adjusted for)	Comparability of groups	Ascertainment of vaccine status	Co-interventions	Ascertainment of mortality	Data & analysis
**Hazard ratio (unadjusted) = 0.78 (0.43, 1.41) Table 3, (group 4 vs. group 2)**	Observational comparison. Children who were born between October 1994 and December 1999 and for whom information is available in at least 1 of the annual vaccination surveys. The survey only collects information for living children, so children who were born and died between successive visits were not included in the database. 24,053 children in the study area of which 17,753 are included and 6,300 are not included. Of the 17,753, mother's information was missing for 0.3% and these children were subsequently excluded resulting in 17,701 children.<br>Selected for inclusion in review: Time after receiving BCG/DTP3 & MCV or BCG and DTP3 [Groups 2 and 4 in the paper].	SES: Mother's education, father's education, compound size.<br>Child's health: Cell location (health services delivery), distance to nearest health facility.<br>Other: Mothers age at birth, mother resident, father resident, age at first observation, distance to Navrongo town.<br>Age: Yes.<br>Gender: Yes.<br>BCG: Yes (all children had received BCG).<br>DTP: Yes (all children had received all 3 doses of DTP).	Vaccines more likely among: No information.<br>Mortality more likely among: No information.	Frequency: Annual collection of vaccination status.<br>Method: Inspection of vaccination cards or other written records.<br>Vaccinated: Children who have a recorded BCG, DTP3 and MCV.<br>Unvaccinated: Children with a recorded BCG and DTP3 vaccines.<br>Dead children: Vaccination status not updated for children who died between birth and subsequent visit.<br>Approach: Probably retrospective (suggestion that retrospectively collected dates of vaccination used in analysis).	DTP: Low probability of differential co-intervention of DTP (only included children who had received all 3 doses of DTP).<br>Other: No information; only 2 months of follow-up used.	Collected at 3 monthly intervals to village compounds and recorded on Navrongo Demographic Surveillance System.	Cox proportional hazards model. Censoring at out-migration.

Very high risk of bias in selection of participants into the study (children included only if seen in annual surveys)
High risk of bias due to confounding (unadjusted analysis of groups)
Very high risk of bias in classification of vaccination status (retrospective approach probably used)
Moderate risk of bias due to deviations from intended interventions
Low risk of bias in measurement of outcomes
Moderate risk of bias due to missing outcome data
Moderate risk of bias in selection of the reported result
Overall: Very high risk of bias (children included only if seen in annual surveys; retrospective approach probably used)

Ghana 1998-2004[5]

Result selected	Sample	Confounders measured (*adjusted for)	Comparability of groups	Ascertainment of vaccine status	Co-interventions	Ascertainment of mortality	Data & analysis
**Hazard ratio (adjusted) = 0.14 (0.13, 0.16) Table II**	Observational comparison. 17,967 children born between 1st January 1998 and 31st December 2004. Selected for inclusion in review: All.	SES: Mother's education*, poverty status*. Child's health: NR. Other: Mother's age. Age: Yes*. Gender: No. BCG: Yes. DTP: Yes.	Vaccines more likely among: BCG recipients. Mortality more likely among: Lower education, higher poverty.	Frequency: Annual collection of vaccination status. Method: Annual survey. Vaccinated: Unclear. Unvaccinated: Unclear. Dead children: No information. Approach: Unclear. Comment: "Annual updates of immunization status and educational attainment and indicators of socioeconomic status are linked to the demographic register."; "Models for the analysis presented here are based on the status of each child at the time of last observation with respect to the WHO definition."	DTP: No information. Other: No information.	All-cause mortality, quarterly visits.	Time conditional HR. No description of any censoring.

Low risk of bias in selection of participants into the study
Very high risk of bias due to confounding (high degree of confounding with BCG and DTP)
Insufficient information to assess risk of bias in classification of vaccination status (high risk of bias or possibly very high risk of bias: cannot tell how vaccination status was defined; retrospective approach may have been used)
Insufficient information to assess risk of bias due to deviations from intended interventions
Low risk of bias in measurement of outcomes
Moderate risk of bias due to missing outcome data
Moderate risk of bias in selection of the reported result
Overall: Very high risk of bias (high degree of confounding with BCG and DTP)

Guinea-Bissau 1978-1983[51]

Result selected	Sample	Confounders measured (*adjusted for)	Comparability of groups	Ascertainment of vaccine status	Co-interventions	Ascertainment of mortality	Data & analysis
**Risk ratio (adjusted) = 0.51 (0.15, 1.81) Computed from counts in Table III (meta-analysis of risk ratios within two time periods)**	Observational comparison. 143 children who were unvaccinated at the beginning of 1981.<br>Selected for inclusion in review: All.	SES: NR.<br>Child's health: weights measured quarterly until 3 years (not reported or used).<br>Other: NR.<br>Age: Yes.<br>Gender: No.<br>BCG: No.<br>DTP: No.	Vaccines were likely among: No information.<br>Mortality was likely among: No information.	Frequency: Quarterly from beginning 1980.<br>Method: Records kept by local health centre.<br>Vaccinated: Children vaccinated since beginning of 1981 according to their central health file.<br>Unvaccinated: Unvaccinated at the beginning of 1981 according to central health file.<br>Dead children: N/A.<br>Approach: Categorization of children by vaccine given or not.	DTP: No information.<br>Other: No information.	Mortality rates during 1 year of follow-up. Interviews were conducted with adult members of the household where measles had been reported to check information on measles infection. Any death within a month of an attack of measles was classified as a measles death.	Death rates during one year of follow-up in children aged 6-11 months at the beginning of 1981.

High risk of bias in selection of participants into the study (enrolment after vaccination likely to have happened)
High risk of bias due to confounding (crudely adjusted analysis)
Moderate risk of bias in classification of vaccination status
Insufficient information to assess risk of bias due to deviations from intended interventions
Low risk of bias in measurement of outcomes
Moderate risk of bias due to missing outcome data
Moderate risk of bias in selection of the reported result
Overall: High risk of bias (enrolment after vaccination likely to have happened; crudely adjusted analysis)

Guinea-Bissau 1978-1983[52]

Result selected	Sample	Confounders measured (*adjusted for)	Comparability of groups	Ascertainment of vaccine status	Co-interventions	Ascertainment of mortality	Data & analysis
**Risk ratio (unadjusted) = 0.62 (0.2, 1.88) Computed from counts in Table 3 (Feb 1981-Mar 1982)**	Observational comparison. 305 children under 5 were examined in June 1979. In March 1982 the registered population of children under 5 was 489.<br>Selected for inclusion in review: Approximately 210 contributing to rates on MCV vs. no MCV.	SES: NR.<br>Child's health: Weight for age (only at start of period in which child suffered measles have been used).<br>Other: Season.<br>Age: Yes.<br>Gender: Yes.<br>BCG: No.<br>DTP: No.	Vaccines more likely among: No information.<br>Mortality more likely among: No information.	Frequency: Not updated<br>Method: No information.<br>Vaccinated: No information.<br>Unvaccinated: No information.<br>Dead children: No information.<br>Comments: Vaccination of measles susceptible children aged 6 months or older was introduced from Feb/March 1981.	DTP: No information.<br>Other: No information.	All cause mortality and measles deaths. "Health problems are discussed with the village committee, which has been given the responsibility of registering deaths." "Inquiries have been made about children who do not turn up for a re-examination in order to find out if they have died, moved or left the area temporarily."	Unadjusted counts provided for vaccinated and unvaccinated children (also rates standardized to one year of follow-up, but not for both comparator groups).

Insufficient information to assess risk of bias in selection of participants into the study
High risk of bias due to confounding (unadjusted analysis, previous vaccines not considered)
Insufficient information to assess risk of bias in classification of vaccination status (cannot tell how vaccination status was defined)
Insufficient information to assess risk of bias due to deviations from intended interventions
Low risk of bias in measurement of outcomes
Moderate risk of bias due to missing outcome data
Moderate risk of bias in selection of the reported result
Overall: High risk of bias (unadjusted analysis)

Guinea-Bissau 1984-1987[55]

Result selected	Sample	Confounders measured (*adjusted for)	Comparability of groups	Ascertainment of vaccine status	Co-interventions	Ascertainment of mortality	Data & analysis
**Hazard ratio (adjusted) = 0.34 (0.17, 0.68)**<br>**Text of paper (p. 1046; value of 2.95 inverted)**	Observational comparison. Children born between August 1984 and September 1985 who were registered in the area by 4 months. Children were followed until June 1987. 274 from Bandim 1 and 448 from Bandim 2.<br>Selected for inclusion in review: All, excluding those vaccinated before 8.5 months of age with MCV.	SES: Area*.<br>Child's health: NR.<br>Other: NR.<br>Age: Yes*.<br>Gender: Yes*.<br>BCG: No.<br>DTP: No.	Vaccines more likely among: No information.<br>Mortality more likely among: No information.	Frequency: Every 3 months (Bandim 1), every 3-5 months (Bandim 2).<br>Method: Vaccination cards.<br>Vaccinated: Vaccine indicated on vaccination card.<br>Unvaccinated: No explicit definition given.<br>Dead children: No information.<br>Approach: "Vaccines regularly monitored in area so undoubtedly few vaccinated children classified as unvaccinated due to loss of the card".	DTP: No information.<br>Other: No information.	Monthly visits in Bandim 1 to register deaths, visits are every 3-5 months in Bandim 2.	Cox proportional hazards model. Censoring at moving away

Moderate risk of bias in selection of participants into the study
High risk of bias due to confounding (no adjustment for child's health or previous vaccines)
Moderate risk of bias in classification of vaccination status
Insufficient information to assess risk of bias due to deviations from intended interventions
Low risk of bias in measurement of outcomes
Moderate risk of bias due to missing outcome data
Moderate risk of bias in selection of the reported result
Overall: High risk of bias (no adjustment for child's health or previous vaccines)
Also from this cohort
Boys vs. girls: Boys RR=0.76 (0.22, 2.64); Girls RR=0.24 (0.09, 0.62); Table 2 (computed from rates, standard group only)[54]

Guinea-Bissau 1990-1996[13]

Result selected	Sample	Confounders measured (*adjusted for)	Comparability of groups	Ascertainment of vaccine status	Co-interventions	Ascertainment of mortality	Data & analysis
**Hazard ratio (adjusted) = 0.48 (0.27, 0.87)**<br>**Text of paper**	Observational comparison. 10,298 children born alive of which 686 died, 90 moved and 770 were too young before the first visit.<br>Selected for inclusion in review: 4230 children aged 7-13 months at second visit and who either had a vaccination card examined or had no card. Children followed-up until next visit or 6 months later if next visit was later than that (children aged up to 18 months).	SES: Cluster*, maternal education, latrine.<br>Child's health: MUAC, well, birth at home.<br>Other: Season, period, maternal tetanus, maternal age, birth order, ethnicity, previous dead children, length of follow-up.<br>Age: Yes*.<br>Gender: Yes.<br>BCG: Yes*.<br>DTP: Yes.	Vaccines more likely among: Children who had more contact with health system, mothers who received tetanus during pregnancy and who gave birth outside the home. They also had larger MUAC, younger mothers, had fewer children, have a latrine and not belong to Balanta or Pepel ethnicity.<br>Mortality more likely among: No information.	Frequency: Visits every 5-7 months on average.<br>Method: Vaccination card.<br>Vaccinated: Children whose card was seen and vaccine recorded.<br>Unvaccinated: Children who had no date or were declared not to have received the vaccine.<br>Dead children: No information<br>Approach: Landmark. Only vaccination status at second visit used (aged 7-13 months).	DTP: Coverage for DTP 1 increased by approximately 10% between 9 and 17 months and by 30% for DTP3 during the same period.<br>Other: No information.	Information on mortality was obtained at subsequent visits (meaning children had to be visited twice to be included in the study).	Cox proportional hazards model. "There was no loss to follow-up because it was always possible to get information on all children from relatives living in the same compound". Analysis repeated excluding children considered unvaccinated because they had no card.

High risk of bias in selection of participants into the study (follow-up starts age 7-13 months)
High risk of bias due to confounding (likely confounding not adjusted for)
Moderate risk of bias in classification of vaccination status
Moderate risk of bias due to deviations from intended interventions
Low risk of bias in measurement of outcomes
Moderate risk of bias due to missing outcome data
Moderate risk of bias in selection of the reported result
Overall: High risk of bias (follow-up starts age 7-13 months; likely confounding)

Guinea-Bissau 1999-2006[60]

Result selected	Sample	Confounders measured (*adjusted for)	Comparability of groups	Ascertainment of vaccine status	Co-interventions	Ascertainment of mortality	Data & analysis
**Hazard ratio (adjusted) = 0.71 (0.56, 0.90) Table 4, all children**	Observational study. Children aged 12-35 months who had their vaccination cards inspected between January 1999 and May 2006. This cohort included 18.119 children of whom 12,119 had their vaccination card inspected.<br>Selected for inclusion in review: Children who had received MCV and unvaccinated children at 12 months of age.	SES: Maternal education, village cluster*, schooling*.<br>Child's health: Arm circumference.<br>Other: Maternal age, ethnicity.<br>Age: Yes*.<br>Gender: Yes.<br>BCG: Yes.<br>DTP: Yes .	Vaccines more likely among: Those with younger mothers, larger mid-upper arm circumference.<br>Mortality more likely among: No information.	Frequency: None in urban area, 6 monthly in rural areas.<br>Method: Vaccination card.<br>Vaccinated: Children with a vaccine recorded.<br>Unvaccinated: Children with no recorded vaccine on card, only included child whose card was seen.<br>Dead children: N/A.<br>Approach: Landmark.	DTP: In 2007 30% of MCV administered simultaneously with or followed by DTP.<br>Other: No information. 26% of measles unvaccinated children who had a card inspected at a subsequent visit had received MCV during follow-up.	Followed through HDSS 6 monthly visits.	Cox proportional hazards model. Effect of survival during following 6 months. Censoring at 6 months.

High risk of bias in selection of participants into the study (children included from 12-23 months, eligible for MCV from 9 months)
High risk of bias due to confounding (no adjustment for child's health)
Moderate risk of bias in classification of vaccination status
High risk of bias due to deviations from intended interventions (co-administration and co-intervention of DTP, unvaccinated children receiving MCV during follow-up)
Low risk of bias in measurement of outcomes
Moderate risk of bias due to missing outcome data
Moderate risk of bias in selection of the reported result
Overall: High risk of bias (children included from 12-23 months; no adjustment for SES or child's health)

Also from this cohort

DTP with MCV vs. MCV after DTP: RR=1.95 (1.12, 3.42) Table 3 (computed from counts)[37]
DTP after MCV vs. MCV after DTP: RR=1.52 (0.71, 3.21) Table 3 (computed from counts)[37]
DTP with or after MCV vs. MCV after DTP: RR=1.72 (0.89, 3.34). Obtained as arithmetic mean of the two results above (on the log scale)[37]
Boys vs. girls: Boys RR=0.87 (0.62, 1.23); Girls RR=0.59 (0.43, 0.80); Table 4 (adjusted MRR)[60]

Guinea-Bissau 2004-2009[43]

Result selected	Sample	Confounders measured (*adjusted for)	Comparability of groups	Ascertainment of vaccine status	Co-interventions	Ascertainment of mortality	Data & analysis
**MRR (adjusted) = 1.22 (0.77, 1.93) Supplementary information (computed from effects for DTP vs MCV and for DTP+MCV vs MCV)**	Randomised trial in children aged 6-23 months in urban and rural areas of HDSS of Bandim Health Project (BHP) due a vaccination.<br>Selected for inclusion in review: 3764 included in the analysis (those receiving either measles or DTP vaccine at enrolment).<br><br>Specific result is an observational comparison	SES: Maternal education, mother signed enrolment form.<br>Child's health: Weight for age, length for age, weight for length, arm circumference for age, maternal arm circumference, breast fed.<br>Other: Rural v urban*, season, ethnicity, age of mother, vitamin A supplementation*.<br>Age: Yes*.<br>Gender: Yes*.<br>BCG: Yes.	Vaccines more likely among: No information.<br>Mortality more likely among: No information.	Vaccines administered at enrolment into trial by government nurses in health centres and by BHP team during outreach campaigns (rural).<br>Method: Home visits and health centres.<br>Vaccinated: Unclear.<br>Unvaccinated: Unclear.<br>Dead children: Follow-up of children who died due to accidents was censored on the date of death.<br>Approach: Landmark, using vaccination provided at VAS administration at start of trial.	BCG: No information.<br>OPV: Co-administered at birth with BCG and with DTP.<br>DTP: Those receiving both DTP and MCV at the start of the trial are excluded from our analysis.<br>Other: Very few with MCV as most recent received any further vaccines.<br>Yellow fever started to be given with MCV during trial. Hepatitis B and H. influenzae type B.<br>At least one third of the children with most recent vaccine as DTP received MCV during follow-up.	Interview to ascertain cause of death.	Moratlity rates presented for children categorized by vaccines provided at enrolment.

Moderate risk of bias in selection of participants into the study
High risk of bias due to confounding (no adjustment for child's health)
Low risk of bias in classification of vaccination status
High risk of bias due to deviations from intended interventions (children subsequently received measles vaccine)
Low risk of bias in measurement of outcomes
Moderate risk of bias due to missing outcome data
Moderate risk of bias in selection of the reported result
Overall: High risk of bias (no adjustment for child's health)

Also from this cohort

Boys vs. girls: Boys RR=1.09 (0.48, 2.49); Girls RR=1.11 (0.54, 2.28); Table 3 (computed from rates)

DTP with MCV vs. MCV after DTP: HR= 3.24 (1.2, 8.73) Table 2[44]

Vitamin A supplementation: Table 3[42]

Haiti 1981-1982[63]

Result selected	Sample	Confounders measured (*adjusted for)	Comparability of groups	Ascertainment of vaccine status	Co-interventions	Ascertainment of mortality	Data & analysis
**Odds ratio (adjusted) = 0.1 (0.02, 0.42)) Table 7 (all children, value 10.0 inverted)**	Observational comparison. Door-to-door survey was done of all households between January and June 1985 to identify mothers who had births between October 1981 and April 1982. 1499 children were born during this period and 1381 survived until 9 months. Case children were born between October 1981 and April 1982 and died between 9 and 39 months. Control children were born in the same time interval and survived until 39 months or were alive at the time of the interview if younger than 39 months.<br>Selected for inclusion in review: All.	SES: Mother literate, mother works, house with cement walls, cement floors, porch on house, electricity in house, ownership of radio, number of rooms in house.<br>Child's health: Nutritional status (breastfeeding / solid foods), hospitalized after 9 months of age.<br>Other: Mother knowledge of rehydration, mother uses family planning, first born, maternal age, birth interval, crowding (more than 5 per house), mother's marital status.<br>Age: Yes.<br>Gender: Yes.<br>Unclear but possible all variables controlled for.<br>BCG: No.<br>DTP: No.	Vaccines more likely among: Literate mothers, mothers with knowledge of rehydration, mothers who work, mothers who use family planning, children not first born, children without cement walls, houses with more than 1 room, mother not owning a radio.<br>Mortality more likely among: Birth interval greater than 24 months, hospitalized children.	Frequency: N/A.<br>Method: Records from MCV trial, clinic records maintained by health centre.<br>Vaccinated: Children with recorded vaccine.<br>Unvaccinated: Children with no recorded vaccine.<br>Dead children: N/A.<br>Approach: Categorization into vaccinated or not.<br>Comments: Of the 620 children who participated in the 1982 MCV study, 306 were identified in 1985. 19 children had received MCV subsequently and the remaining children were unvaccinated.	DTP: No information.<br>Other: No information.	Information was obtained during the interview about any deceased children including date and age of child.	Stepwise logistic regression stratified by confounders. Deaths after 39 months of age were excluded from the analysis.

High risk of bias in selection of participants into the study (restricted to children who survived to 9 months and whose mother still around 4-5 years after birth)
High risk of bias due to confounding (unadjusted analysis)
Moderate risk of bias in classification of vaccination status
Insufficient information to assess risk of bias due to deviations from intended interventions
Low risk of bias in measurement of outcomes
Moderate risk of bias due to missing outcome data
Moderate risk of bias in selection of the reported result
Overall: High risk of bias (restricted to children who survived to 9 months and whose mother still around 4-5 years after birth; unadjusted analysis)

India 1986-1991[64]

Result selected	Sample	Confounders measured (*adjusted for)	Comparability of groups	Ascertainment of vaccine status	Co-interventions	Ascertainment of mortality	Data & analysis
**MRR (unadjusted) = 0.34 (0.23, 0.51) Computed from cumulative frequencies in Table 3, with test-based confidence interval from log rank test**	Observational comparison. 16,665 births were registered during the study period of which 3531 were excluded as they left the study area soon after birth to return to their father's home. Selected for inclusion in review: Children who received MCV between 6-8 months of age and survived to 12 months. After 1100 excluded who had vaccine after 12 months: N=12034.	SES: No land, maternal education, paternal education, hut/kutcha house. Child's health: NR. Other: NR. Age: Yes*. Gender: Yes*. BCG: Yes (collected but not reported). DTP: Yes (collected but not reported).	Vaccines more likely among: Families with land and parents with some education. Mortality more likely among: No information.	Frequency: Weekly visits to give information, child register updated periodically. Method: Collected at weekly visits. Vaccinated: Recorded measles vaccination. Unvaccinated: No information. Dead children: No information. Approach: Vaccination status prior to start of follow-up used to categorize children.	DTP: Coverage for DTP was over 90% at 12 months of age. No other information provided (censored at time of subsequent vaccinations). Other: No information.	Weekly visits, deaths are reported by the part-time community health worker to the health aide. Simple verbal autopsy.	Unimmunized children who subsequently received immunization were censored at the time of immunization. Children who received vaccination after 12 months were excluded from further follow-up. Inclusion in the analyses was conditional on surviving the first year of life.

High risk of bias in selection of participants into the study (vaccine given from 6 months, had to survive to 12 months)
High risk of bias due to confounding (unadjusted analysis)
Moderate risk of bias in classification of vaccination status
Insufficient information to assess risk of bias due to deviations from intended interventions
Low risk of bias in measurement of outcomes
Moderate risk of bias due to missing outcome data
Moderate risk of bias in selection of the reported result
Overall: High risk of bias (children had to survive to 12 months; unadjusted analysis)

India 1987-1989[19]

Result selected	Sample	Confounders measured (*adjusted for)	Comparability of groups	Ascertainment of vaccine status	Co-interventions	Ascertainment of mortality	Data & analysis
**MRR (unadjusted) = 0.31 (0.12, 0.8) Text of paper (p. 7305)**	Observational comparison. Live births between December 1987 and November 1989 in 45 contiguous villages. Analysis files from a previous study available for 4138 live births (286 deaths). Of these, 255 records were missing sex, birthday or exit day, which left 3883 children (282 deaths).<br>Selected for inclusion in review: Unvaccinated children or children with MCV.	SES: NR.<br>Child's health: Birth weight, current weight, length/height, common childhood morbidities.<br>Other: NR.<br>Age: Yes.<br>Gender: Yes.<br>DTP: Yes.<br>BCG: Yes.	Vaccines were likely among: No information.<br>Mortality was likely among: No information.	Frequency: 3 monthly visits.<br>Method: Vaccination card at home or when the child was bought to the clinic for immunization.<br>Vaccinated: Children with a recorded MCV.<br>Unvaccinated: Children without a recorded vaccine at 12 months, children whose vaccination card wasn't seen.<br>Dead children: Data were available for 282/286 dead children.<br>Approach: Children categorized by known vaccination status at 2-12 months.	DTP: Moderate probability of DTP co-intervention (some children had DTP out of sequence or simultaneous with MCV).<br>Other: No information.	All-cause mortality likely collected at 3 monthly visits at home.	Unadjusted analysis based on mortality rates in first 12 months.<br>Follow-up from date of known MCV.

Moderate risk of bias in selection of participants into the study
High risk of bias due to confounding (unadjusted comparison)
High risk of bias in classification of vaccination status (unvaccinated group included those children whose card wasn't seen)
High risk of bias due to deviations from intended interventions (some risk of DTP co-intervention)
Low risk of bias in measurement of outcomes
Moderate risk of bias due to missing outcome data
Moderate risk of bias in selection of the reported result
Overall: High risk of bias (unadjusted comparison; assumptions about non-vaccination)

Also from this paper

DTP with MCV vs. MCV after DTP: HR=4.77 (0.33, 70.18) Table 6
DTP after MCV vs. MCV after DTP: HR=15.89 (2.21, 118.99) Table 6
DTP with or after MCV vs MCV after DTP: HR=9.14 (1.24, 67.3) Text p. 7306

India 1991-1998[65]

Result selected	Sample	Confounders measured (*adjusted for)	Comparability of groups	Ascertainment of vaccine status	Co-interventions	Ascertainment of mortality	Data & analysis
**OR (adjusted) = 0.36 (0.23, 0.56) Text of paper (after stratification, p. 246 (inverted))**	Case-control study.<br>Children aged 12-59 months born from 1991-1998 (MCV from 10 months). The controls were chosen from a cohort of 15,578 born during the same period and alive at the time of the study. They were matched for age, sex, family size and area of residence. Overall 318 cases and controls formed matched pairs.<br>Selected for inclusion in review: All.	SES: Area* (matching), mother's literacy, fathers education, father's occupation, caste*.<br>Child's health: NR.<br>Other: Mother's age, father's age, mother employed, family size* (matching).<br>Age: Yes* (matching).<br>Gender: Yes* (matching).<br>BCG: Yes (collected but not reported).<br>DTP: Yes (collected but not reported).	Vaccines more likely among: No information.<br>Mortality more likely among: No information.	Frequency: NR ("routine visits").<br>Method: Project database examined to identify children who had died between 12-59 months.<br>Vaccinated: No details, likely children recorded as receiving MCV.<br>Unvaccinated: No details, likely children with no recorded MCV.<br>Dead children: N/A.<br>Approach: Exposure defined as vaccinated before 12 months old.	DTP: No information.<br>Other: No information.	Collected as part of the Comprehensive Rural Health Services Project at Ballabgarh, no specific details provided.	Adjusted odds ratio.

Moderate risk of bias in selection of participants into the study
High risk of bias due to confounding (matching and adjustment unlikely to address all confounders)
Moderate risk of bias in classification of vaccination status
Insufficient information to assess risk of bias due to deviations from intended interventions
Low risk of bias in measurement of outcomes
Moderate risk of bias due to missing outcome data
Moderate risk of bias in selection of the reported result
Overall: High risk of bias (matching and adjustment unlikely to address all confounders)

India 2006-2011[21]

Result selected	Sample	Confounders measured (*adjusted for)	Comparability of groups	Ascertainment of vaccine status	Co-interventions	Ascertainment of mortality	Data & analysis
**MRR (unadjusted) = 0.41 (0.24, 0.7) Computed from rates in Table 3 (9-15 months), BCG, DTP + MCV v BCG + DTP**	Observational comparison. Live study births from 1 January 2006 to 31 December 2011 in 28 villages in Ballabgarh. A total of 12,142 births with immunization details were available. A total of 11,390 had complete information on confounders and were included in analyses.<br>Selected for inclusion in review: Children whose most recent vaccine was DTP or MCV between 9-15 months.	SES: Mother's education, fathers education, caste, wealth index.<br>Child's health: Access to health care, presence of a health facility in the village.<br>Other: Birth order.<br>Age: Yes.<br>Gender: Yes.<br>BCG: Yes.<br>DTP: Yes.	Vaccines more likely among: No information.<br>Mortality more likely among: No information.	Frequency: Monthly work plan.<br>Method: Fortnightly visits added to computer database.<br>Vaccinated: Recorded as having received MCV as last vaccine based on computerized system (approx. 98% had received BCG and DTP).<br>Unvaccinated: Recorded as having received DTP as last vaccine based on based on computerized system.<br>Dead children: N/A.<br>Approach: Used date of vaccination, although likely to be quite accurate.	DTP: Low probability of differential co-intervention of DTP (98% had received DTP prior to MCV).<br>Other: No information is provided about any of the co-interventions.	Visits/ computerized system.	Unadjusted mortality ratios. Children included in each group from receipt of vaccine until receipt of another vaccine or any other exit criteria. Exit criteria were 3 years of age, end of study or migration. Sensitivity analyses conducted using 2 week lag period.

Moderate risk of bias in selection of participants into the study
Very high risk of bias due to confounding (unadjusted comparison of time after BCG vaccine with time after DTP vaccine)
Moderate risk of bias in classification of vaccination status
Moderate risk of bias due to deviations from intended interventions
Low risk of bias in measurement of outcomes
Moderate risk of bias due to missing outcome data
Moderate risk of bias in selection of the reported result
Overall: Very high risk of bias (unadjusted comparison of time after BCG vaccine with time after DTP vaccine)

Also from this paper

Boys vs. girls: Boys RR=0.42 (0.18, 0.98); Girls RR=0.40 (0.20, 0.82); Table 3 (computed from rates)

Malawi 1995-1997[22]

Result selected	Sample	Confounders measured (*adjusted for)	Comparability of groups	Ascertainment of vaccine status	Co-interventions	Ascertainment of mortality	Data & analysis
**Hazard ratio (adjusted) = 0.42 (0.16, 1.14) Table 2B (children present) MCV v No MCV**	Observational comparison. Live births between July 1995 and February 1997. The cohort includes approximately 95% of newborn children (N=803). Of these, 36 were stillborn and 16 died during first week (no vaccines).<br>Selected for inclusion in review: Children present at monthly anthropometric examinations. 669 children in period 9 months to 18 months of age,	SES: Maternal schooling, district.<br>Child's health: Weight for age, weight for height, twinning.<br>Other: HIV status of mother*, birth order, season of birth, religion, maternal age.<br>Age: Yes*.<br>Gender: Yes.<br>BCG: Yes.<br>DTP: Yes.	Vaccines more likely among: No information.<br>Mortality more likely among: No information<br>(but *associated* with HIV infection, twinning, religion, district, travelling and weight for age).	Frequency: Monthly visits up until 18 months, quarterly from 18 to 60 months.<br>Method: Vaccination card, information verified from health centre records.<br>Vaccinated: Vaccine recorded on card / health centre records.<br>Unvaccinated: Assumed children without evidence of vaccination were unvaccinated.<br>Dead children: No information but used both methods.<br>Approach: Landmark.<br>Comment: "Almost all children received the vaccines in the planned sequence."	DTP: Low probability of differential co-intervention of DTP (almost all children received vaccines in the planned sequence).<br>Other: No information provided for any pre-defined co-interventions.	No information, likely collected at monthly visits.	Cox proportional hazards model; no information about censoring. Absent children censored in analysis until they were again examined. No information about amount of missing data for dead children in retrospective approach.

Low risk of bias in selection of participants into the study
High risk of bias due to confounding (no adjustment for SES or child's health)
High risk of bias in classification of vaccination status (assumptions about non-vaccination)
Moderate risk of bias due to deviations from intended interventions
Low risk of bias in measurement of outcomes
Moderate risk of bias due to missing outcome data
Moderate risk of bias in selection of the reported result
Overall: High risk of bias (no adjustment for SES or child's health; assumptions about non-vaccination)

Also from this paper

DTP with MCV vs. MCV after DTP: HR=5.27 (1.11, 25) page 724

Boys vs. girls: Boys RR=0.62 (0.22, 1.80); Girls RR=0.23 (0.04, 1.27); Table 2A

Papua New Guinea 1989-1994[23]

Result selected	Sample	Confounders measured (*adjusted for)	Comparability of groups	Ascertainment of vaccine status	Co-interventions	Ascertainment of mortality	Data & analysis
**Hazard ratio (adjusted) = 0.48 (0.18, 1.26) Table 6 (6-11 months)**	Observational comparison. Children born under demographic surveillance, registered within 60 days of birth and who survived more than 28 days were included. 6665 children born between 1989 and 1994. 2617 were excluded for a number of reasons. Thus 4048 were included in survival analysis. Selected for inclusion in review: 2618 who had received BCG before 6 months.	SES: Region. Child's health: Twin. Other: Hep B*, pneumococcal vaccine*, birth order, birth year*, death of older sibling, birth interval from previous sibling, multiple births, mother's age*, propensity score. Age: Yes*. Gender: Yes. BCG: All children had received BCG. DTP: Yes* (number of doses).	Vaccines more likely among: Children born to mothers less than 23 and older than 35. Mortality more likely among: No information.	Frequency: Monthly clinics but frequent disruptions. Clinic cards bought to office monthly for entry of dates. Method: Clinic cards held by nurses and child's health books kept by mothers. Vaccinated: Vaccination recorded on clinic records. Unvaccinated: No vaccine recorded on clinic cards. Dead children: Cards kept by nurses for 1 year after last attendance, likely to have information for dead children. Approach: Unclear; vaccination information appears to be prospectively recorded for living and dead children.	DTP: Coverage for DTP2 increased by 21% between 6 and 12 months of age and by 37% for DTP3. Other: No information. Large increase in pneumococcal polysaccharide (PncPS) vaccine during follow-up, may be associated with MCV.	Reported during monthly demographic surveillance. Determined by verbal autopsy.	Cox proportional hazards model. Censoring at death, migration, 6 months or the end of the study period.

Moderate risk of bias in selection of participants into the study
High risk of bias due to confounding (no adjustment for SES or child's health)
Moderate risk of bias in classification of vaccination status
High risk of bias due to deviations from intended interventions (risk of co-intervention with DTP and PncPS)
Low risk of bias in measurement of outcomes
Moderate risk of bias due to missing outcome data
Moderate risk of bias in selection of the reported result
Overall: High risk of bias (no adjustment for SES or child's health; risk of co-intervention with DTP and PncPS)

Senegal 1985-1987[67]

Result selected	Sample	Confounders measured (*adjusted for)	Comparability of groups	Ascertainment of vaccine status	Co-interventions	Ascertainment of mortality	Data & analysis
**MRR (unadjusted) = 0.99 (0.72, 1.37) Computed from rates in Table 2 (1985-1987, 9-23 months)**	Observational comparison. Children born to a resident mother between February 1985 and January 1987. Of 2,417 children born during this period 2,093 were still under surveillance at 9 months of age. No routine MCV until late 1986. Age of vaccination was much higher and more children were vaccinated after 12 months of age. Selected for inclusion in review: 2030 (assumed) with known vaccination status.	SES: NR Child's health: Measles infection. Other: Season, year of birth, death of mother. Age: Yes. Gender: Yes. BCG: No. DTP: No.	Vaccines more likely among: No information. Mortality more likely among: Rainy season.	Frequency: Annual census. Method: Vaccination cards for annual census visit. Vaccinated: Vaccine recorded on card. Unvaccinated: No vaccine recorded on card. Dead children: No information. Approach: Landmark.	DTP: No information. Other: No information.	Cause of death obtained through parental post-mortem interviews reviewed by 2 physicians who were blind to vaccine group.	Only included children who received the vaccine from 6 months. Children vaccinated after having had measles infection were considered unvaccinated. A few children who received HT MCV after STD MCV were censored at the day of revaccination.

Moderate risk of bias in selection of participants into the study
High risk of bias due to confounding (age-unadjusted analysis of groups)
High risk of bias in classification of vaccination status (annual data collection)
Insufficient information to assess risk of bias due to deviations from intended interventions
Low risk of bias in measurement of outcomes
Moderate risk of bias due to missing outcome data
Moderate risk of bias in selection of the reported result
Overall: High risk of bias (age-unadjusted analysis of groups; annual data collection)
Also from this paper
Boys vs. girls: Boys RR=1.28 (0.85, 1.94); Girls RR=0.69 (0.41, 1.17); Table 2 (computed from rates)

Senegal 1987-1989[67]

Result selected	Sample	Confounders measured (*adjusted for)	Comparability of groups	Ascertainment of vaccine status	Co-interventions	Ascertainment of mortality	Data & analysis
**MRR (unadjusted) = 0.68 (0.42, 1.1) Computed from rates in Table 2 (1987-1989, 9-23 months)**	Observational comparison. Children born to a resident mother between February 1987 and January 1989. 2,467 children born during this time. Of these 2,118 lived in the study area after the age of 9 months. MCV trial during this time period, placebo group and those who did not receive their vaccine were offered MCV at 10 months. Selected for inclusion in review: 1159 (assumed) with known vaccination status.	SES: NR. Child's health: Measles infection. Other: Season, year of birth, death of mother. Age: Yes. Gender: Yes. BCG: No. DTP: No.	Vaccines more likely among: No information. Mortality more likely among: Rainy season.	Frequency: Annual census which was supplemented with weekly surveillance visits to all compounds in the study area. Method: Most MCV has been provided by the project. Vaccinated: Vaccine provided by project. Unvaccinated: No vaccine provided by project. Dead children: No information. Approach: Date of vaccination known.	DTP: No information. Other: No information.	Cause of death obtained through parental post-mortem interviews reviewed by 2 physicians who were blind to vaccine group.	Compared from 9 months as this has been the official age for receiving MCV. Only included children who received the vaccine from 6 months. Children vaccinated after having had measles infection were considered unvaccinated. A few children who received HT MCV after STD MCV were censored at the day of revaccination.

Moderate risk of bias in selection of participants into the study
High risk of bias due to confounding (age unadjusted analysis of groups)
Moderate risk of bias in classification of vaccination status
Insufficient information to assess risk of bias due to deviations from intended interventions
Low risk of bias in measurement of outcomes
Moderate risk of bias due to missing outcome data
Moderate risk of bias in selection of the reported result
Overall: High risk of bias (age unadjusted analysis of groups)

Also from this paper

Boys vs. girls: Boys RR=0.86 (0.44, 1.67); Girls RR=0.51 (0.24, 1.05); Table 2 (computed from rates)

Senegal 1989-1996[25]

Result selected	Sample	Confounders measured (*adjusted for)	Comparability of groups	Ascertainment of vaccine status	Co-interventions	Ascertainment of mortality	Data & analysis
**Hazard ratio (unadjusted) = 0.98 (0.75, 1.27) Table 3 (first cohort)**	Observational comparison. 8277 children born between Sept 1989 and August 1996. Children were excluded if they received BCG and DTP on different occasions. This resulted with 7796 children.<br>Selected for inclusion in review: All.	SES: Maternal education.<br>Child's health: NR.<br>Other: Number of siblings who died before 2, number of siblings, mother's age, birth rank, BCG/DTP.<br>Age: Yes.<br>Gender: Yes.<br>BCG: Yes.<br>DTP: Yes.<br>MCV was too strongly associated to assess the association between MCV and mortality separately among recipients and non-recipients of BCG/DTP.	Vaccines more likely among: Children who had received BCG, DTP.<br>Mortality more likely among: No information.	Frequency: Weekly.<br>Method: Provided by project team.<br>Vaccinated: Children vaccinated by the project team.<br>Unvaccinated: Children not vaccinated by project team.<br>Dead children: No information.<br>Approach: Categorization.<br>Comments: "The project vaccinated all infants born in or migrated into the study area; hence vaccination status was completely accurate."	DTP: Ignored further doses of DTP.<br>Other: Also ignored further doses of yellow fever vaccine, polio and meningitis vaccine.	During weekly visits.	Proportional hazards model with time varying covariates. Children were included at birth and followed up to 2 years of age (or death or outmigration). Children receiving vaccines after inclusion were censored.

Moderate risk of bias in selection of participants into the study
Very high risk of bias due to confounding (unadjusted analyses of groups, very high level of confounding by BCG/DTP)
Low risk of bias in classification of vaccination status
Insufficient information to assess risk of bias due to deviations from intended interventions
Low risk of bias in measurement of outcomes
Moderate risk of bias due to missing outcome data
Moderate risk of bias in selection of the reported result
Overall: Very high risk of bias (unadjusted analyses of groups, very high level of confounding by BCG/DTP)

Also from this paper

Boys vs. girls: Boys RR=0.43 (0.22, 0.85); Girls RR=0.61 (0.23, 1.61); Table 2 (computed from rates)

Senegal 1996-1999[24]

Result selected	Sample	Confounders measured (*adjusted for)	Comparability of groups	Ascertainment of vaccine status	Co-interventions	Ascertainment of mortality	Data & analysis
**Hazard ratio (adjusted) = 0.55 (0.31, 0.98) Table 1 (MCV with no known concurrent or subsequent DTP)**	Observational comparison. 4133 children born and registered in study area between Sept 1996 and Dec 1999. 4102 included in the analyses. Selected for inclusion in review: 832 children who received MCV according to the WHO strategy.	SES: Health centre area*. Child's health: NR. Other: Year of vaccination*, season of vaccination*. Age: Yes*. Gender: Yes*. BCG: All children had received BCG. DTP: All children had received DTP.	Vaccines more likely among: No information. Mortality more likely among: No information.	Frequency: Every 3 months after mid 1997. Method: Project records and then vaccination card. Vaccinated: All vaccines are provided and recorded by project team until mid 1997. Following this children with a noted vaccine and date were considered vaccinated. Unvaccinated: Those with no recorded vaccine. After mid 1997 this also includes children with no information available. Dead children: Generally no information provided. Approach: Landmark.	DTP: Low probability of differential DTP co-intervention (analysis of children who received MCV with no known concurrent or subsequent DTP). Other: No information.	No information.	Cox proportional hazards model. Censored at 24 months of age, registration of next vaccine, death or migration.

Moderate risk of bias in selection of participants into the study
High risk of bias due to confounding (no adjustment for child's health)
High risk of bias in classification of vaccination status (children with no information included as unvaccinated, and further bias towards null from landmark approach)
Moderate risk of bias due to deviations from intended interventions
Low risk of bias in measurement of outcomes
Moderate risk of bias due to missing outcome data
Moderate risk of bias in selection of the reported result
Overall: High risk of bias (no adjustment for child's health; assumptions about vaccination)

Also from this paper

DTP with MCV vs. MCV after DTP: HR=1.96 (0.95, 4.04) Table 1 (computed from HRs)
DTP after MCV vs. MCV after DTP: HR=2.40 (1.00, 5.75) Table 1 (computed from HRs)
DTP with or after MCV vs. MCV after DTP: HR=2.05 (1.16, 3.62) Table 1 (computed from HRs)
Boys vs. girls: Boys RR=0.85 (0.32, 2.25); Girls RR=0.35 (0.12, 1.03); Table 1 (computed from rates)

Appendix 6: Sensitivity analyses

Restriction to hazard ratios and mortality rate ratios

Since odds ratios (OR) and risk ratios (RR) do not account for differences in follow-up between groups, we repeated analyses restricted to hazard ratios (HR) and mortality rate ratios (MRR).

- For BCG, the overall random-effects estimate from clinical trials is 0.70 (0.49 to 1.01) when including all estimates, and 0.60 (0.39 to 0.91) after removing two RRs. Across the observational studies, the overall random-effects estimate is 0.47 (0.32 to 0.69) when including all estimates, and 0.44 (0.29 to 0.68) after removing one OR.
- For DTP, the overall random-effects estimate from observational studies is 1.38 (0.92 to 2.08) when including all estimates, and 1.32 (0.86 to 2.04) after removing one OR.
- For MV, the overall random-effects estimate from clinical trials is 0.74 (0.51 to 1.07) when including all estimates, and 0.79 (0.53 to 1.19) after removing two risk ratios. Regarding observational studies, the overall random-effects estimate is 0.51 (0.42 to 0.63) when including all estimates, and 0.56 (0.45 to 0.70) after removing two risk ratios and three odds ratios.

Alternative statistical methods

	BCG (clinical trials)	BCG (observational studies)	DTP (observational studies)	MCV (clinical trials)	MCV (observational studies)
DerSimonian-Laird[a] variance estimate, Z-based confidence intervals	0.70 (0.49 to 1.01)	0.47 (0.32 to 0.69)	1.38 (0.92 to 2.08)	0.74 (0.51 to 1.07)	0.54 (0.44 to 0.67)
REML variance estimate, Z-based confidence intervals	0.70 (0.48 to 1.01)	0.47 (0.32 to 0.70)	1.39 (0.91 to 2.10)	0.74 (0.51 to 1.07)	0.54 (0.43 to 0.68)
Paule-Mandel[b] variance estimate, Z-based confidence intervals	0.71 (0.50 to 1.00)	0.46 (0.29 to 0.73)	1.39 (0.91 to 2.12)	0.74 (0.51 to 1.07)	0.54 (0.43 to 0.67)
DerSimonian-Laird variance estimate, Hartung-Knapp[c] confidence intervals	0.70 (0.43 to 1.15)	0.47 (0.28 to 0.79)	1.38 (0.85 to 2.25)	0.74 (0.45 to 1.22)	0.54 (0.43 to 0.68)
REML variance estimate, Hartung-Knapp confidence intervals	0.70 (0.42 to 1.15)	0.47 (0.28 to 0.79)	1.39 (0.85 to 2.26)	0.74 (0.45 to 1.22)	0.54 (0.43 to 0.67)
Paule-Mandel variance estimate, Hartung-Knapp confidence intervals	0.71 (0.44 to 1.15)	0.46 (0.27 to 0.80)	1.39 (0.85 to 2.26)	0.74 (0.45 to 1.22)	0.54 (0.42 to 0.68)

[a]DerSimonian R, Laird N. Meta-analysis in clinical trials. *Controlled Clinical Trials* 1986; **7**: 177-88
[b]Paule RC, Mandel J. *Consensus values and weighting factors*. National Institute of Standards and Technology, 1982.
[c]Hartung J, Knapp G. A refined method for the meta-analysis of controlled clinical trials with binary outcome. *Statistics in Medicine*. 2001; **20**: 3875-89.
